# Mechanism of Antiradical Activity of Newly Synthesized 4,7-Dihydroxycoumarin Derivatives-Experimental and Kinetic DFT Study

**DOI:** 10.3390/ijms222413273

**Published:** 2021-12-09

**Authors:** Žiko Milanović, Dušan Dimić, Milan Žižić, Dejan Milenković, Zoran Marković, Edina Avdović

**Affiliations:** 1 Department of Science, Institute for Information Technologies, University of Kragujevac, Jovana Cvijića bb, 34000 Kragujevac, Serbia; ziko.milanovic@uni.kg.ac.rs (Ž.M.); deki82@kg.ac.rs (D.M.); 2 Department of Chemistry, Faculty of Science, University of Kragujevac, Radoja Domanovića 12, 34000 Kragujevac, Serbia; 3 Faculty of Physical Chemistry, University of Belgrade, 12-16 Studentski trg, 11000 Belgrade, Serbia; ddimic@ffh.bg.ac.rs; 4 Life Sciences Department, Institute for Multidisciplinary Research, University of Belgrade, Kneza Višeslava 1, 11030 Belgrade, Serbia; mzizic@imsi.bg.ac.rs

**Keywords:** 4,7-dihydroxycoumarin, antiradical activity, DFT, EPR, radical scavenging, hydroxy radical, QM-ORSA

## Abstract

Coumarin derivatives have proven beneficial biological activities, but the mechanism of their radical scavenging potency is not fully understood. In this study, the antiradical capacity of two newly synthesized 4,7-dihydroxycoumarin derivatives: (*E*)-3-(1-((3-hydroxy-4-methoxyphenyl)amino)-ethylidene)-2,4-dioxochroman-7-yl acetate (**A-3OH**) and (*E*)-3-(1-((4-hydroxy-3-methoxyphenyl)amino)ethylidene)-2,4-dioxochroman-7-yl acetate (**A-4OH**) towards HO^•^ were examined by Electron Paramagnetic Resonance (EPR) Spectroscopy and Density Functional Theory (DFT). The compounds were fully characterized by the elemental microanalysis, IR, and NMR spectroscopies. The effect of pH on the acid–base equilibria is separately discussed and the predominant species at the physiological pH were determined. Several common mechanisms (Hydrogen Atom Transfer (HAT), Single-Electron Transfer followed by Proton Transfer (SET-PT), Sequential Proton Loss followed by Electron Transfer (SPLET), Radical Adduct Formation (RAF), and Intramolecular Hydrogen Atom Abstraction (*i*HAA)) of radical scavenging were investigated based on thermodynamic and kinetic parameters. EPR results indicated that both compounds significantly reduce the amount of present HO^•^. The results of the kinetic DFT study demonstrated that both compounds predominantly exhibit antiradical capacity through HAT and SPLET mechanisms. The estimated overall rate constants (*k*_overall_) proved that **A-4OH** shows better antioxidant capacity than **A-3OH** which is well-correlated with the results obtained by EPR measurement.

## 1. Introduction

Reactive oxygen species (ROS) are present in small concentrations in the aqueous medium [1]. Most often, they are short-lived and highly reactive species that are capable of oxidizing molecules, including biologically important macromolecules [2,3]. ROS, in very low concentration, participate in the transformation of harmful substances in the aquatic environment in the Advanced Oxidation Processes [4,5]. In this way, the concentration of harmful substances is reduced along with the neutralization of free radicals, as described in the previous research [6,7]. On the other hand, ROS are formed by various metabolic and oxidative processes in the organism. The most common ROSs in the human organism are the superoxide and hydroxide radicals. The entire course of formation of a highly reactive HO^•^ species is known as the Haber–Weiss reaction [8]. In addition, radicals present in low concentrations contribute to the normal homeostasis of the organism. Excessive production of radical species caused by various external factors contributes to the occurrence of oxidative stress [9]. An imbalance in the process of production/neutralization of ROS results in the appearance of various pathophysiological conditions in the organism [10,11,12]. The high reactivity of the formed radical species causes oxidative damage to biomolecules, changes in cell permeability, signaling, protein replication, etc., which contributes to the development of the pathophysiological response of the organism [13]. 

Available literature data indicate that hydrophilic coumarin derivatives show a positive effect in preventing, compensating, and removing ROS in the aqueous (cytosolic) compartment of the cell [14,15]. The reaction mechanism between coumarin and HO^•^ can be complex, as several products can be formed [16]. Various polyphenolic-type coumarin derivatives have shown the effect of removing HO^•^ radical [17,18,19] that was higher than for the standard radical scavengers [20]. The antioxidant, antiinflammatory, antidyslipidemic, and anti-HIV activities of coumarin derivatives were proven [21,22,23]. Density functional theory gave useful results for the elucidation of the structural parameters important for the radical scavenging activity [24,25]. The determination of these structural parameters could potentially lead to the application of artificial intelligence for the prediction of the course of chemical reactions and the antiradical potency of known and new compounds [26,27,28,29]. 

Within this work, the antioxidant potency of newly synthesized 4,7-dihydroxycoumarin derivatives with amino-methoxyphenol was investigated. These compounds were structurally characterized by the NMR, IR, and elemental microanalysis. Using sophisticated experimental (Electron Paramagnetic Resonance-EPR) and computational ((Density Functional Theory-DFT)/QM-ORSA (Quantum Mechanics-based test for Overall free Radical Scavenging Activity) protocol)) methods, the reactivity, and mechanism of radical scavenging activity of these compounds against HO^•^ were analyzed in detail. The applied QM-ORSA protocol and the chosen theoretical model have proven to reproduce well the results of experimental studies of antioxidant capacity in the following references [30,31,32]. The acid–base equilibria of the obtained substances were included in the discussion, as the ability to remove reactive radial species depends on the pH of the reaction medium and the degree of deprotonation. The investigation of the proportion of the different acid–base forms to react with ROS, at a particular pH, makes the mechanisms of antiradical action comprehensive and more complex. For this reason, the investigation was performed at a physiologically relevant pH value (7.4).

## 2. Materials and Methods

### 2.1. Chemicals and Instrumentations

Chemicals used in the experiments were obtained from Merck (Darmstadt, Germany) except for spin-trap DEPMPO which was purchased from Enzo Life Sciences (Farmingdale, NY, USA). All of the chemicals were of the analytical grade. The IR vibrational spectra were recorded on the Perkin-Elmer Spectrum (Bruker, Germany. Model: 197). One FT-IR spectrometer using the KBr pellet technique. The NMR spectra were obtained on the Varian Gemini spectrometer (Varian, Palo Alto, CA, 200 MHz for ^1^H and 50 MHz for ^13^C) in DMSO-*d6*. The elemental microanalysis (C, H, N) was performed on the Elemental analysis system VARIO EL III CHNOS, model—Elementar Analysensysteme (GmbH, VarioEL, Germany), 2003 in the Centre for Instrumental Analysis, at the Faculty of Chemistry, University of Belgrade. 

### 2.2. General Synthesis Procedure

**Scheme 1:**
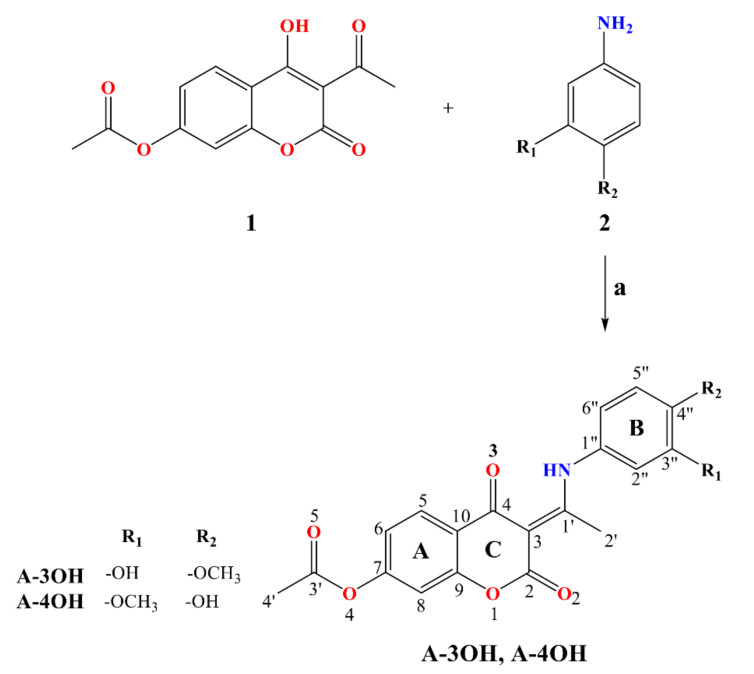
General procedure for the synthesis of coumarin derivatives with atomic numbering. Reagents and conditions: EtOH, reflux, 3 h.

Compound 3-acetyl-4-hydroxy-2-oxo-2H-chromen-7-yl acetate (**1**) was prepared as described in reference [33]. New coumarin derivatives (**A-3OH** and **A-4OH**) were synthesized in the reaction of the previously synthesized compound **1** and aniline derivatives (**2**) (0.002 mol): 4-amino-2-methoxyphenol and 5-amino-2-methoxyphenol, at 3h reflux in ethanol (50 mL) (Scheme 1). The progress of the reaction was monitored by thin-layer chromatography (TLC) (toluene:acetone = 7:3). When the reaction was complete, the resulting mixture was cooled to room temperature and the precipitate was collected by filtration. The obtained products were later purified from ethanol. All newly synthesized compounds were characterized by elemental analysis, ^1^H NMR, ^13^C NMR, and IR spectroscopy.

(*E*)-3-(1-((3-hydroxy-4-methoxyphenyl)amino)ethylidene)-2,4-dioxochroman-7-yl acetate (**A-3OH**), Yield, 0.395 g (79.16%). **^1^H NMR** (DMSO-*d6*, 200 MHz) (δ ppm): 2.31 (3H, s, C4–’H), 2.58 (3H, s, C2’–H), 3.82 (3H, s, OCH_3_), 6.81 (2H, m, C2’’–H, C6’’–H), 7.10 (3H, m, C6–H, C8–H, C5’’–H), 8.01 (1H, d, ^3^*J*_H-5,H-6_ = 8.4 Hz, C5–H), 9.55 (1H, s, OH), 15.24 (1H, s, NH). **^13^C NMR** (DMSO-*d6*, 50 MHz) (δ ppm): 20.54 (C2’), 21.06 (C4’), 55.97 (OCH3), 96.76 (C3), 109.85 (C2’’), 112.52 (C8), 112.78 (C5’’), 116.34 (C10), 117.68 (C6’’), 118.05 (C6), 127.11 (C5), 128.63 (C1’’), 147.21 (C4’’), 147.71 (C3’’), 153.82 (C7), 154.85 (C9), 161.44 (C2), 168.65 (C1’), 175.67 (C3’), 179.54 (C4). **IR (KBr)** ν cm^−1^: 3267 (O-H), 3072 (N-H), 1772, 1697 (C=O), 1614, 1578, 1518, 1468 (C=C), 1213 (C-O).

(*E*)-3-(1-((4-hydroxy-3-methoxyphenyl)amino)ethylidene)-2,4-dioxochroman-7-yl acetate (**A-4OH**), Yield, 0.389 g (77.42%). **^1^H NMR** (DMSO-*d6*, 200 MHz) (δ ppm): 2.31 (3H, s, C4’-H), 2.60 (3H, s, C2’-H), 3.36 (3H, s, OCH3), 6.86 (1H, m, C5’’–H), 7.03(2H, m, C2’’–H, C6’’–H), 7.13(2H, m, C6–H, C8–H), 8.01(1H, d, ^3^*J*_H-5,H-6_ = 8.5 Hz, C5–H), 9.49 (1H, s, OH), 15.25(1H, s, NH). **^13^C NMR** (DMSO-*d6*, 50 MHz) (δ ppm): 20.62 (C2’), 21.06 (C4’), 56.05 (OCH3), 96.70 (C3), 109.87 (C2’’), 110.10 (C8), 115.62 (C10), 117.71 (C5’’), 118.02 (C6’’), 118.07 (C6), 127.10 (C5), 127.14 (C1’’), 146.21 (C4’’), 148.21 (C3’’), 153.83 (C7), 154.85 (C9), 161.48 (C2), 168.66 (C1’), 175.82 (C3’), 179.51 (C4). **IR (KBr)** ν cm^−1^: 3327 (O-H), 3064 (N-H), 1767, 1693 (C=O), 1622, 1572, 1512, 1464 (C=C), 1207 (C-O).

### 2.3. EPR Measurement with HO^•^ Radical

EPR experiments were carried out at room temperature (293 K) on Bruker EMX Nano X-band (9.65 GHz) spectrometer, using the following operating conditions: power attenuation, 10 dB; modulation amplitude, 2 mT; modulation frequency, 100 kHz; sweep time, 120 s. Hydroxyl radical was produced by employing the standard Fenton mechanism (1 mM H_2_O_2_ and 0.33 mM FeSO_4_) with the addition of 0.1 M DEPMPO as a spin trapping agent in 100 mM phosphate buffer (pH 7.4). Spectra were collected 180 s after the addition of the appropriate iron catalyst. The spin trap was purified according to the procedure proposed by Jackson [34]. The stock solutions (15 mM) of both compounds were prepared in DMSO and diluted with water to 10 μM. The blank sample contained the same amount of DMSO as samples with **A-3OH** and **A-4OH**. The final concentration of investigated substances was 0.75 μM. The reactivity of compounds towards HO^•^ was determined as the relative decrease in the average intensity of the two most intense peaks of DEPMPO- HO^•^ adduct at the low-field part of the spectrum. The result is expressed as the % of radical reduction = 100 × (I_0_ − I_a_)/I_0_. In the previous equation, I_a_ and I_0_ are the intensities of peaks of DEPMPO- HO^•^ adduct with and without **A-3OH** and **A-4OH**. 

### 2.4. Computational Methodology

All calculations were performed by employing the *Gaussian09* program package [35]. The structures of investigated molecules were optimized using quantum chemical calculations within the frame of the Density Functional Theory, M06-2X/6-311++G(d,p) theoretical model (with polarization and diffuse functions included) [36]. Available literature and data from several independent authors indicate that the applied theoretical model is suitable for thermodynamic and kinetic investigations [37,38,39,40]. The conformational search was performed to verify that the global minima were obtained. These results were partially based on the rotation of the C7-O4 bond for a structurally similar molecule as given in [33] and the fact that intramolecular hydrogen bond is formed when the OH group points towards –OCH3 group. The solvent effect was approximated by the Conductor-like Polarizable Continuum Model (CPCM) using methanol (ε = 32.61) and water (ε = 78.36) as solvents to mimic experimental environments [41]. IRC calculations were performed to confirm that the geometry of transition states connects the pre-reaction and post-reaction complexes along the reaction path. The theoretical NMR spectra were obtained by the Gauge Independent Atomic Orbital (GIAO) approach after the reoptimization of structures in DMSO. All of the chemical shifts were calculated relative to TMS. The population analysis was done by the Natural Bond Orbital (NBO) method [42], as implemented in the Gaussian 09 package. NBO analysis highlights the role of the intermolecular interactions and atomic net charges for the investigated species that are important for defining their reactivity [43,44]. 

The assessment of the mechanisms of antiradical action presented in this research was based on thermodynamic and kinetic considerations and is in line with the QM-ORSA (Quantum Mechanics-based test for Overall free Radical Scavenging Activity) methodology [45]. After estimating the Gibbs free energy of the reaction, all exergonic (Δ_r_*G* < 0) and isoergonic (Δ_r_*G* < 0) reaction pathways were subjected to kinetic calculations. The rate constants (*k*) were calculated using the conventional transition state theory (TST) or Eyring equation:

(1) $$k_{TST}=\frac{k_{B}T}{h}\exp\left(\frac{-\Delta G_{a}^{{}^{\neq }}}{RT}\right)$$

where *k*_B_ and *h* are the Boltzmann and Planck constants; Δ*G_a_*^≠^ is the Gibbs free energy of activation. 

The previous equation is based on the laws of classical mechanics and does not include quantum effects such as tunneling. In general, the tunneling effect is important for reactions involving light particles (for example protons or hydrogen atoms) that penetrate through the barrier along with the reaction coordinate. Ignoring quantum effects would cause major errors in estimating the rate constants. Eckart’s method, which represents the special case of the zero-curvature tunneling approach (ZCT_0), includes the necessary quantum effects in the expression for the rate constant of a chemical reaction:

(2) $$k_{ZCT\_0}=\sigma \gamma (T)\frac{k_{B}T}{h}\exp\left(\frac{-\Delta G^{\neq }}{RT}\right)$$

where *σ* represents the reaction path degeneracy, accounting for the number of equivalent reaction paths; and *γ(T)* accounts for tunneling corrections [45]. For these calculations, the *TheRate* program was used [46]. 

For reactions involving electron transfer, the transition state cannot be located by electronic structural methods. Therefore, important kinetic parameters were estimated using the Marcus theory, as described in previous research [47,48]. 

Estimation of the overall rate constant (*k*_overall_), in an aquatic medium, offers a more accurate picture of the reactivity of the investigated compounds than other possible indexes. The *k*_overall_ is the sum of the product of the molar fractions of acid–base species and the total rate constant (*k*_tot_). The *k*_tot_ includes the sum of all thermodynamically favored reaction pathways [41]. A detailed description of the estimate of the *k*_overall_, as well as the process of the quantification molar fraction of each acid–base species, in the aqueous medium at physiological pH, is given in the Supplementary Material (SM, eq. 1s-8s). Additionally, the relative antioxidant capacity (r^T^) relative to Trolox (**Tx**) as well relative amounts of products (%), i.e., branching ratios (Γ*_i_*) were calculated following the equation given in SM (eq. 9s and 10s).

## 3. Results

### 3.1. Chemistry

The structural characterization of the newly obtained 4,7-dihydroxycoumarin derivatives includes the IR and NMR spectroscopies. Due to the similarity in structure, except for the relative positions of –OH and –OCH_3_ groups, the spectra show a great resemblance. In the IR spectrum, there is a wide peak belonging to the stretching O-H vibration at 3267 (**A-3OH**) and 3327 (**A-4OH**) cm^−1^. A very wide peak below 3100 cm^−1^ is due to the presence of the N–H group that is part of the quasi six-membered ring enclosed by a strong hydrogen bond. A similar fragment has been observed in structurally similar molecules [49,50]. In the IR spectrum, bands assigned to the C=O, C=C, and C-O vibrations were detected. The broad singlets in the ^1^H NMR spectrum at 15.24 ppm confirmed the presence of N-H protons, while the peaks at 9.4 were assigned to O–H protons. The resonant maxima of the aromatic protons of the 4,7-dihydroxycoumarin part were positioned between 7 and 8 ppm. The protons belonging to the aromatic core of the substituent are located between 6 and 8 ppm. The methoxy group protons give an intense signal at 2.31 ppm for both molecules. The presence of *sp*^2^ hybridized carbon atoms is shown in ^13^C NMR spectra in the range between 96.8 and 179 ppm. The resonant maxima corresponding to carbon atoms at the C4 position of both compounds are positioned close to 179.0 ppm. The lowest chemical shifts were obtained for C2′ (20 ppm) and C4′ (21 ppm), as expected since these carbon atoms are *sp^3^* hybridized. The resonant maxima of the carbon atom of the methoxy group are positioned at 56 ppm both in **A-3OH** and **A-4OH**. In addition, the corresponding chemical NMR shifts in DMSO were estimated using the same theoretical model and GIAO approach. Experimental ^1^H and ^13^C (ppm), as well as simulated chemical shifts for the synthesized compounds, are given in Tables S1 and S2 (atom numbering follows that in the scheme). Based on the comparison, it can be concluded that the experimental values for newly synthesized compounds were well reproduced with correlation coefficients of 0.999. Values for MAE are 0.18, 0.06 for ^1^H NMR and 1.79, 2.03 for ^13^C NMR. These results prove the applicability of the chosen level of theory for the description of the experimental parameters. 

### 3.2. The Experimental HO^•^ Scavenging Activity

**Figure 1:**
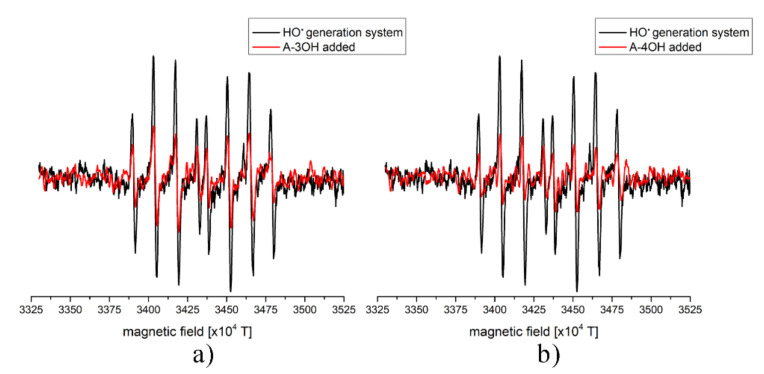
The experimental EPR spectra of DEPMPO-HO^•^ with and without compounds (**a**) **A-3OH** and (**b**) **A-4OH**.

The experimental reactivity of the obtained compounds towards HO^•^ was monitored by the EPR spectroscopy. The spectra were recorded 180 s after the reaction start. The radical was generated in the Fenton system and DEPMPO was used as a spin trap. The EPR spectra with and without compounds **A-3OH** and **A-4OH** are given in Figure 1. The addition of coumarin derivatives leads to a decrease in peak intensity. The scavenging activities, calculated as given in the Methodology sections, are 56 and 70% for **A-3OH** and **A-4OH**, respectively. The higher reactivity of **A-4OH** can be explained by the presence of the OH group in *para*-position and the extended delocalization upon the radical formation. A detailed quantum chemical analysis of the mechanism is considered in the following sections, including the discussion on the role of acid–base equilibria for this process which might influence the overall radical-scavenging activity through the introduction of novel reaction pathways. Except for the direct exchange of protons, there are several other reaction routes, including the radical adduct formation and the electron transfer that are possible for these compounds. These mechanisms are important for the removal of the radical species from the wastewaters and the formation of less toxic products [51,52,53]. 

### 3.3. Acid–Base Equilibria

**Figure 2:**
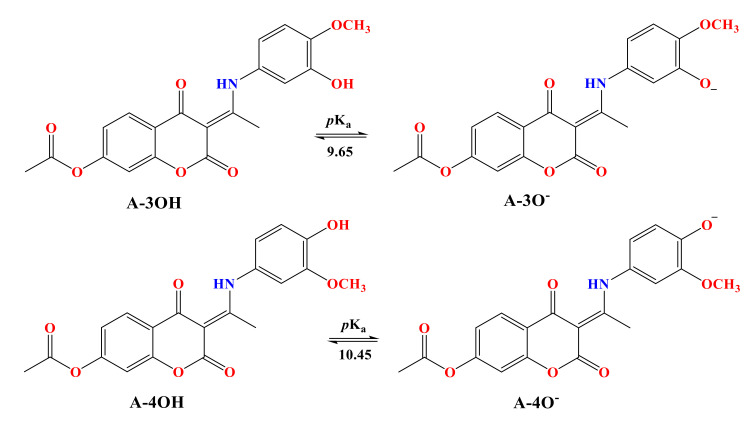
Possible deprotonation pathways and estimated *p*Ka values of the investigated compounds.

**Figure 3:**
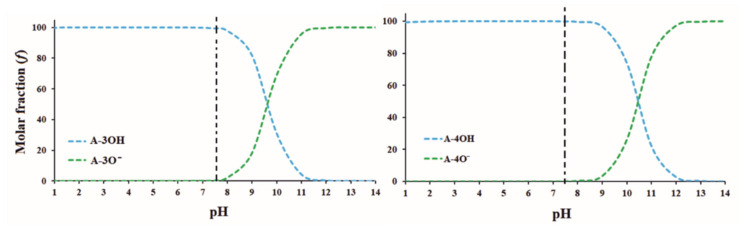
Graph of distribution of molar fraction (f) of acid–base forms of the investigated compounds at different pH values. Blackline represents physiological pH (7.4).

Acid–base equilibria have a strong effect on the antioxidant properties of investigated compounds. Therefore, it is desirable to determine the *p*Ka values before testing the antioxidant capacity. For these reasons, *p*Ka values for both compounds were determined using the ACD/*p*Ka software package [54]. The deprotonation pathways of the investigated compounds, as well as the estimated *p*Ka values, are shown in Figure 2, while the corresponding values for the molar fraction (*f*) at a given pH (eq. 1s-5s) are given graphically and quantitatively in Figure 3 and Table S3. 

As shown in Figure 2, the possible deprotonation route happens through the –OH group. The *p*Ka values differ for almost one unit between **A-3OH** (9.65) and **A-4OH** (10.45). It is important to notice that the position and presence of the –OH group were discussed as the main factors influencing the radical scavenging activity towards HO^•^. The proportion of neutral and deprotonated acid–base species varies depending on pH values. In the pH range values below 8, both compounds almost exclusively exist in neutral form. In the range of 9 < pH < 10 neutral and deprotonated acid–base forms are represented in the different percentages. In a much more alkaline medium of pH > 11, deprotonated forms are dominant in the largest percentage. This means that if the –OH group is deprotonated, mechanisms that don’t include direct hydrogen atom transfer from the electronegative group are the dominant ones. At the physiological pH, the neutral acid–base species is represented in the largest molar fraction (99.4% for **A-3OH** and 99.9% for **A-4OH**). Due to this fact, further theoretical investigations are performed on the neutral species **A-3OH** and **A-4OH**. 

### 3.4. Thermodynamic Approach between Neutral Species and HO^•^ Radical

To assess the antiradical activity of **A-3OH** and **A-4OH** against HO^•^ better, the following mechanistic pathways were examined: Hydrogen Atom Transfer (HAT), Single-Electron Transfer followed by Proton Transfer (SET-PT), Sequential Proton Loss followed by Electron Transfer (SPLET), Radical Adduct Formation (RAF), and Intramolecular Hydrogen Atom Abstraction (*i*HAA) (3–9):

HAT: A-OH + HO^•^ → A-O^•^ + H_2_O (3)

RAF: A-OH + HO^•^ → [HO-A-OH]^•^ (4)

*i*HAA: [HO-A-OH]^•^ → A-O^•^ + H_2_O (5)

SET: A-OH + HO^•^ → A-OH^•+^ + HO^−^ (6)

PT: A-OH^•+^ + HO^−^ → A-O^•^ + H_2_O (7)

SPL: A-OH + HO^−^ → A-O^−^ + H_2_O (8)

ET: A-O^−^ + HO^•^ → A-O^•^ + HO^−^ (9)

**Figure 4:**
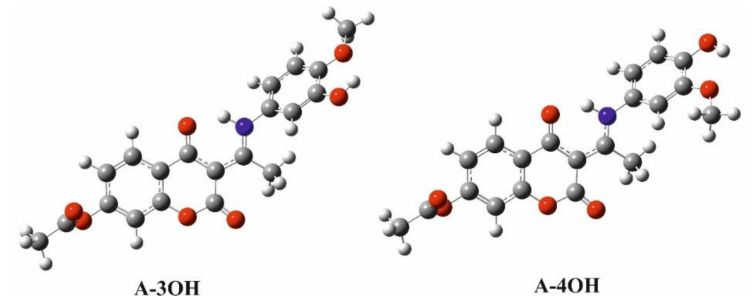
Optimized geometry of investigated compounds at M06-2X/6-311++G(d,p) level of theory.

**Figure 5:**
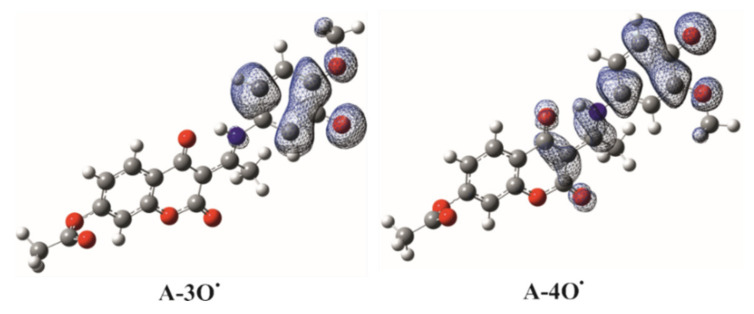
Spin density distribution maps (0.002 electron/bohr3) for formed radical species. The blue color represents the positive spin density.

The optimized geometries of the investigated compounds are shown in Figure 4. The changes in the Gibbs reaction energies (Δ_r_*G*) for the given mechanisms are listed in Table 1. The possible reaction centers for HAT, SPLET, and SET-PT mechanisms are the –OH groups, while for RAF all of the carbon atoms of 4,7-dihydroxycoumarin and aromatic substituent are included. Concerning the HAT mechanism (Table 1), it is clear that **A-4OH** (−141 kJmol^−1^) exhibits a slightly better ability to neutralize HO^•^ than **A-3OH** (−135 kJmol-1), which is supported by the experimental results. The explanation for the reactivity towards radical species lies in the stability of the newly formed radical species (Figure 5 and Figure S1) and extended delocalization of the unpaired electron within the structure of **A-4O^•^**. Lower spin density is located on the O-4 atom of **A-4O^•^** (0.321*e*) when compared to the same atom of **A-3O^•^** (0.330*e*) which is a consequence of better delocalization of the unpaired electron (Figure S4).

The radical adduct formation usually occurs when *sp*^2^ hybridized carbon atoms are present in the structure. It is obvious from Table 1 that the formation of radical adducts in the reaction of the tested compounds and HO^•^ is thermodynamically favored, which is reflected in the exergonic values of Δ_r_*G*_RAF_. The presented values are very similar between **A-3OH** and **A-4OH**, although the values for Δ_r_*G*_RAF_ of **A-4OH** are lower for several kJmol^−1^. Both aromatic rings are possible reaction sites along with one active position in the aliphatic chain (C1′). The most reactive positions for the formation of a radical adduct are C-3″ (**A-3OH**, −50 kJmol^−1^) and C-4″ (**A-4OH**, −57 kJ mol^−1^) positions, namely the carbon atoms with OH group attached. In the optimized geometries of adducts (Figures S5 and S6), the planarity and aromaticity of the system are disturbed by the rehybridization of a carbon atom, from *sp*^2^ to *sp*^3^, to which the –OH group is attached.

A very interesting mechanism, previously postulated, was observed after the addition of HO^•^ [43]. Namely, the attached HO^•^ in adjacent positions of the aromatic –OH group of **A-3OH** (C-2**″** and C-4**″**) and **A-4OH** (C-3**″** and C-5**″**) causes intramolecular separation of water molecules by Intramolecular Hydrogen Atom Abstraction (*i*HAA) mechanism. The Δ_r_*G*_iHAA_ values are between −86 and −115 kJmol^−1^, which is much more spontaneous than for the RAF mechanism. From Table 1, it is clear that the intramolecular separation of water molecules is most thermodynamically favored in the C-5**″** position of the A-4OH (−115 kJmol^−1^). This joint mechanism, RAF-iHAA, can also be used for the explanation of the radical scavenging activity of compounds that don’t contain good hydrogen atom donating groups. Further kinetic studies are undertaken to exploit additional reasons for the higher reactivity of **A-4OH**.

The first step of the SPLET mechanism is characterized by the proton loss (Δ_r_*G*_SPL_) and formation of the corresponding phenoxy anions while the second step denotes the electron transfer from the obtained phenoxy anions (Δ_r_*G*_ET_). The similarity of the obtained value for Δ_r_G_SPL_ **A-3OH** (−93 kJmol^−1^) and **A-4OH** (−92 kJmol^−1^) can be explained by the distribution of NBO charges in the corresponding anions (Figure S7). The reactivity of formed anionic species is reflected in the more exergonic Δ_r_*G*_ET_ value of **A-4O^−^** (−50 kJmol^−1^) compared to **A-3O^–^** (−42 kJmol^−1^) for the second step of the SPLET mechanism which includes electron transfer. As both of the mentioned steps influence the thermodynamic spontaneity of the process the sum of Δ_r_*G*_SPL_ and Δ_r_*G*_ET_ has been taken as a comparison parameter. The sum of the values for **A-4OH** is about 6 kJ mol^−1^ more favorable than the same parameter for **A-3OH** (Table 1). Along with the previously discussed mechanism, this result is well-correlated with the experimental observations. 

Electron transfer (SET, Equation (6)) between neutral forms of investigated compounds and HO^•^ is not thermodynamically favorable, which is reflected in the distinctly endergonic Δ_r_*G*_SET_ values for **A-3OH** (Δ_r_G_IP_ = 105 kJmol^−1^) and **A-4OH** (Δ_r_G_IP_ = 100 kJmol^−1^) (Table 1). Therefore, this mechanism is not thermodynamically plausible and is excluded from the kinetic studies.

### 3.5. Reactions A-3OH and A-4OH with HO^•^ Radical-Kinetic Approach

Thermodynamically favored reaction pathways (Δ_r_*G* ≤ 0), between **A-3OH**, **A-4OH**, and HO^•^, were subjected to kinetic investigation. Unfortunately, a geometry of transition states for the reactions of the investigated compounds with HO^•^ via the HAT mechanism was not found. To obtain a possible explanation, the dependence of total energy (a.u) on HO–H (Å) distance as scan coordinate was monitored and the results are presented in Figure S8. These reactions take place very quickly, without an energy barrier, i.e., that their rate constants are controlled by diffusion (1.91 × 10^9^ M^−1^ s^−1^, Table 2) [43]. Therefore, this result cannot be taken for the comparison between **A-3OH** and **A-4OH** reactivity towards HO^•^. 

**Figure 6:**
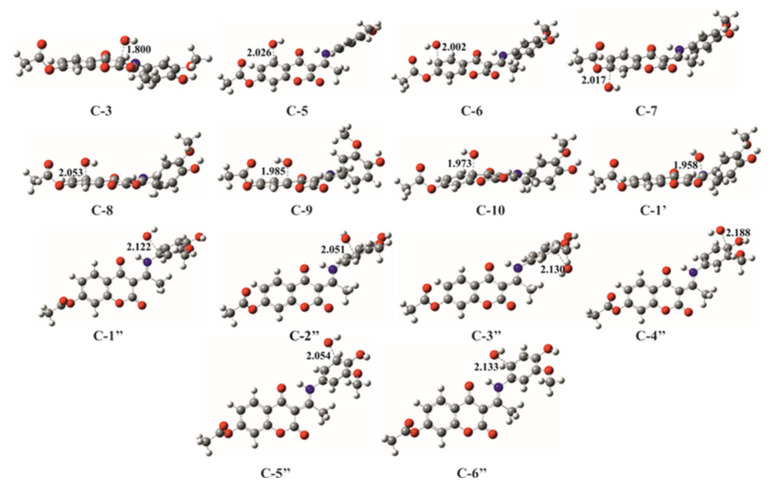
Optimized transition state geometries for the formation of radical adducts of **A-4OH** compounds in water at M06-2X/6-311++G(d,p) level of theory.

Another, less probable, reaction pathway for the reaction of HO^•^ with investigated compounds is RAF, with the Δ*G*_a_ values between 34 and 57 kJmol^−1^. All positions on the coumarin part of the molecules, as well as on the aromatic ring B, were examined. The obtained values for rate constants are given in Table 2–while the optimized geometries of the corresponding transition states are presented in Figure 6 and Figure 6 and Figure S6. As can be seen by comparing the values of thermodynamic and kinetic parameters, the values of the rate constant correlate well with the thermodynamic parameters. It means, that the thermodynamically most favorable positions are in good accordance with kinetically most favorable positions. It is obvious that the aromatic ring significantly contributes to the improvement of antioxidant properties of these compounds because the rate constants of chemical reactions with the investigated radical are in some cases up to 1000 times faster than for the rest of carbon atoms, Table 2. The kinetic results also follow the experimentally determined higher reactivity of **A-4OH**, as the Δ*G*_a_ values are lower for several kJmol^−1^.

Large intra-atomic distances in transition states for **A-3OH** and **A-4OH** between 2.130 to 2.190 Å, are accompanied by low values of activation energies and large values of rate constants (Table 2), which is shows that these are early transition states. The dependence of ln*k*_TST_ and ln*k*_ZCT_0_ on reciprocal temperature for RAF was examined. The obtained results are presented in Figure S10. From the presented results it can be concluded that the values of the rate constants deviate significantly at all temperatures. This phenomenon is probably a consequence of low activation energy, i.e., a flat surface of potential energy. The conventional TST method, in this case, overestimates the values of the rate constants and is not suitable for estimating the rate constants at room temperature.

**Figure 7:**
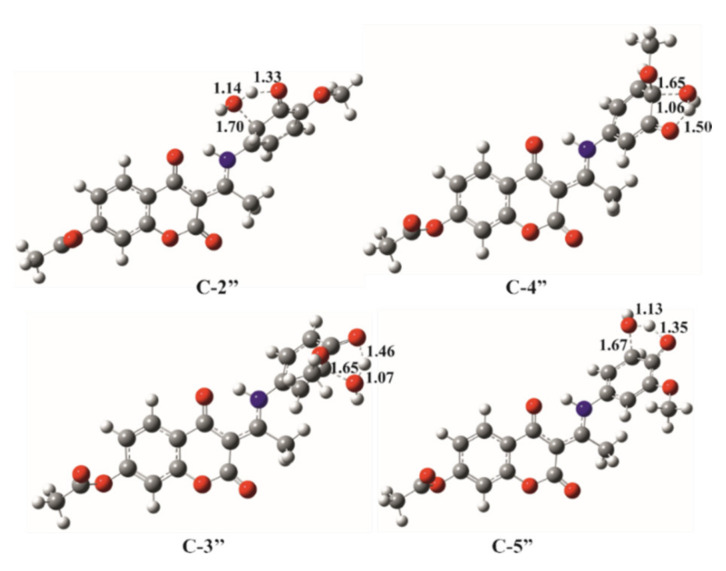
Optimized transition state geometries of the *i*HAA mechanism at a different position for **[HO-A-3OH]^•^** (up) and **[HO-A-4OH]^•^** (down).

On the other hand, the geometry search led to interesting structures that were assumed to correspond to the geometry of the transition states in the intermediate process of the intramolecular separation of water molecules from formed radical adducts (Figure 7). This process of Intramolecular Hydrogen Atom Abstraction (*i*HAA) takes place after the RAF mechanism, so the overall reaction is characterized as the RAF-*i*HAA mechanism.

**Scheme 2:**
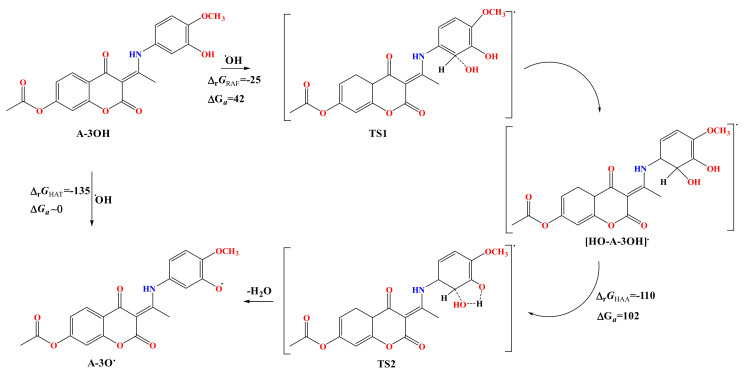
Mechanisms of radical adduct formation (RAF) and intramolecular separation of a water molecule (*i*HAA) at C2**″** position of **A-3OH**.

As shown in Scheme 2, the first step RAF-*i*HAA involves the formation of adducts in adjacent positions of the –OH group (C2”, C4” positions of **A-3OH** and C3”, C5” positions of **A-4OH**). This reaction flows through the transition state geometries (Figure 6), thus forming a radical adduct (Figures S5 and S6) that is at a lower energy level than the reactants. These radicals can be stabilized by delocalization of the spin density via the parent molecule (Figure S11) or by an intramolecular reaction followed by the separation of one molecule of water. Intramolecular separation of water goes through the geometry of the transition state (Figure 7), which leads to the formation of a radical that is obtained by hydrogen atom abstraction. The geometries of transition states for all reactions which take place via *i*HAA were confirmed by IRC calculation (Figures S12–S15).

**Figure 8:**
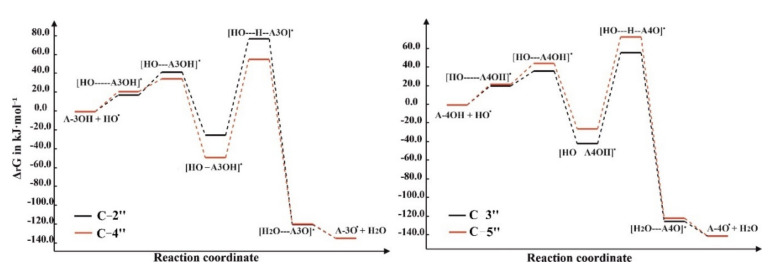
Energy profiles of reactions of **A-3OH** (**left**) and **A-4OH** (**right**) with HO^•^ that take place according to RAF followed by the *i*HAA reaction mechanism.

Corresponding energy profiles of reactions of **A-3OH** (C2″ and C4″) and **A-4OH** (C3″ and C5″) with HO^•^ are presented in Figure 8, Analysis of the data from Table 2 as well as the energy profile for the *i*HAA reaction (Figure 8) shows that this reaction step is much slower than the first step, and this step determines the rate of a chemical reaction. Nominal values of activation energies for positions C-2” and C-4” at **A-3OH** and C-3″ and C-5″ at **A-4OH** are slightly more than 100 kJ mol^−1^ (Table 2). Such relatively high values of activation energy indicate the fact that the second step, the separation of water molecules from radical adducts, is very slow at room temperature. The values of the reaction rate constants, which are of the order of 10^−5^ s^−1^, also support this.

**Figure 9:**
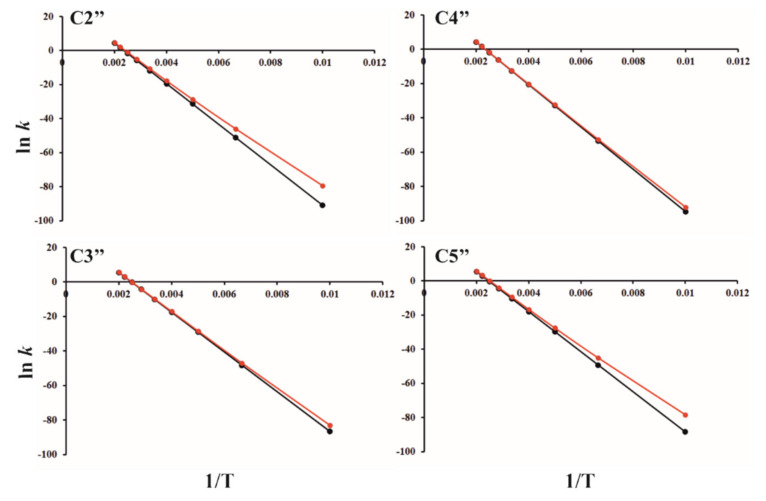
Dependence of ln*k*_TST_ (black) and ln*k*_ZCT_0_ (red) on reciprocal temperature for intramolecular reaction (*i*HAA mechanism): **[HO-A-3OH]^•^** (up), **[HO-A-4OH]^•^** (down).

Analysis of *k*_TST_ and *k*_ZCT_0_ values shows that rate constants at 298 K for the two intermediates in both positions in the *i*HAA mechanism practically coincide (Table 2), indicating that both TST and Eckart methods are suitable for estimating the rate constants for these reactions, at room temperature. Differences between the values of the rate constants are noticeable at lower temperatures (Figure 9) for the reaction in position C2” for **[HO-A-3OH]^•^** and C5” for **[HO-A-4OH]^•^**. These differences between *k*_TST_ and *k*_ZCT_0_ values can be attributed to the tunneling effect, especially since the *i*HAA mechanism involves hydrogen atoms that can penetrate through activation barriers. As seen in Figure 9, the tunneling effect decreases rapidly with the temperature increase. This is in line with the well-known fact that the Eckart method at low temperatures overestimates this effect.

The first step of the SPLET mechanism does not go through a transition state. The rate constants of proton transfer from **A-3OH** and **A-4OH** on HO^–^ are played by the diffusion rate (1.91 × 10^9^ M^−1^s^−1^) (Figure S16). The second step of this reaction is expressed via the reaction rate constant for electron transfer between **A-3O^–^** and **A-4O^–^** and the HO^•^ radical. The activation energy was estimated according to Marcus’ theory (Table 2). The rate constants for both steps indicate that it also takes place at a rate controlled by diffusion. Based on all that has been said, it is clear that the SPLET mechanism is an important reaction pathway for the antiradical activity of the investigated compounds.

### 3.6. Relative Antiradical Capacity (r^T^) and Branching Ratios (Γ_i_)

The overall rate constants (*k*_overall_) were estimated according to the QM-ORSA methodology given in the Supplementary material (Eq. 6s-8s, Table S5). The *k*_overall_ for **A-3OH** (5.06 × 10^9^ M^−1^s^−1^) and **A-4OH** (9.49 × 10^9^ M^−1^s^−1^) in aqueous solution and physiological pH, indicate a potentially good capacity in scavenging HO^•^. The *k*_overall_ value of the Trolox, a common standard for the radical scavenging activity, is 1.94 × 10^9^ M^−1^s^−1^. This value is estimated under the same conditions and from the available literature data [17]. The r^T^ values indicate that both compounds show better scavenging capacity against the HO^•^ radical than **Tx** (Table S5).

Additionally, the capacity of **A-4OH** is about 1.85 times greater than that of **A-3OH**, which correlates with the experimental values (1.25 times). This comparison proves the applicability of the QM-ORSA methodology for predicting the radical scavenging capacity and a better understanding of the underlying mechanisms of activity. To estimate the relative amounts of products as well as the impact of the individual reaction pathways on the antioxidant capacity the branching ratios (*Γ_i_*) were calculated (Table S5). Radicals in the HAT mechanism were formed with the branching ratios of 35.39 (**A-3OH**) and 19.34% (**A-4OH**). On the other hand, anions were formed with the branching ratios of 25.57 (**A-3OH**) and 59.12% (**A-4OH**) while the radicals formed during electron transfer are represented by 35.39 (**A-3OH**) and 19.34% (**A-4OH**). Our results in Table S5 unequivocally indicate that HAT and SPLET are the dominant mechanisms against the HO^•^. As shown in previous chapters, the higher activity of **A-4OH** is a consequence of the increased thermodynamic stability of the radical/anion which is later well-reflected in the kinetic parameters.

## 4. Conclusions

Two 4,7-dihydroxycoumarin derivatives with methoxy-aminophenols, (*E*)-3-(1-((3-hydroxy-4-methoxyphenyl)amino)-ethylidene)-2,4-dioxochroman-7-yl acetate (**A-3OH**) and (*E*)-3-(1-((4-hydroxy-3-methoxyphenyl)amino)ethylidene)-2,4-dioxochroman-7-yl acetate (**A-4OH**) were obtained under mild conditions. The bands in the IR spectrum showed the presence of the N-H group that is part of the quasi six-membered ring enclosed by a strong hydrogen bond and O-H group. The chemical shifts in the NMR spectrum proved the suggested structure. The EPR measurements showed significant antiradical activity of the obtained compounds towards HO^•^. The percentages of reduction were 56% for **A-3OH** and 70% for **A-4OH**. The predicted *p*Ka values were 9.65 and 10.45 for **A-3OH** and **A-4OH**, respectively. At the physiological pH value, the dominant form is the neutral one. The thermodynamically plausible mechanisms were Hydrogen Atom Transfer (HAT), Sequential Proton Loss followed by Electron Transfer (SPLET), and Radical Adduct Formation (RAF). Almost all of the carbon atom positions were the active centers for the RAF mechanism. Neighboring atoms to the carbon atom with –OH group attached underwent a second reaction, Intramolecular Hydrogen Atom Abstraction (*i*HAA). The reaction rates for the HAT mechanism were of the order of 10^9^ M^−1^s^−1^ and the kinetic curves proved that this reaction occurred without the transition state. The electron-transfer reaction rates, calculated according to Marcus’ theory, were of the same order of magnitude. What differed between the two obtained compounds were the activation energy and reaction rate constants for the RAF mechanism. These reaction rates were between 10^4^ and 10^7^ M^−1^s^−1^. The overall rate constants were 5.06 × 10^9^ M^−1^s^−1^ (**A-3OH**) and 9.49 × 10^9^ M^−1^s^−1^ (**A-4OH**), both of which were higher than the calculated one for Trolox, standard antioxidant. The ratio between the reaction rates coincided well with the ratio between the reduction percentages obtained by EPR. This proved the applicability of the predicted mechanisms and pave the path for the future analysis of the antiradical mechanisms of coumarin derivatives.

## Figures and Tables

**Table 1:** Estimated values of Gibbs reaction energies (Δ_r_*G*) in kJ mol^−1^ for reactions between newly coumarin derivatives and HO^•^.

Species	HO^•^				
	HAT	SET-PT		SPLET	
	Δ_r_*G*_HAT_	Δ_r_*G*_SET_	Δ_r_*G*_PT_	Δ_r_*G*_SPL_	Δ_r_*G*_ET_
**A-3OH**	−135	105	−240	−93	−42
**A-4OH**	−141	100	−241	−92	−50
Positions	**RAF/HAA** Δ_r_*G*_RAF_/Δ_r_*G*_HAA_				
	**A-3OH**			**A-4OH**	
**C-3**	−6			−10	
**C-5**	−40			−41	
**C-6**	−17			−21	
**C-7**	−41			−43	
**C-8**	−33			−34	
**C-9**	−31			−30	
**C-10**	0			0	
**C-1′**	−25			−25	
**C-1″**	−36			−42	
**C-2″**	−27/−108			−21	
**C-3″**	−50			−42/−99	
**C-4″**	−49/−86			−57	
**C-5″**	−19			−26/−115	
**C-6″**	−44			−41	

**Table 2:** Activation energies (Δ*G*_a_) reaction and rate constants for the various mechanistic pathways estimated by Eckart’s method (*k*_ZCT_0_).

Species	HAT		SPLET			
			I Step		II Step	
	***ΔG_a_***, **kJ mol^−1^**	$k_{HAT}$ **M^−1^s^−1^**	***ΔG_a_*** **kJ mol^−1^**	$k_{SPL}$ **M^−1^s^−1^**	***ΔG_a_*** **kJ mol^−1^**	$k_{ET}$ **M^−1^s^−1^**
**A-3OH**	~0	1.91 × 10^9^	~0	1.91 × 10^9^	20	1.38×10^9^
**A-4OH**	~0	1.91 × 10^9^	~0	1.91 × 10^9^	15	5.84×10^9^
Position	**RAF/HAA**					
	**A-3OH**			**A-4OH**		
	*ΔG_a_* kJ mol^−1^	$k_{\mathrm{RAF}}^{\mathrm{ZCT}\_0}$$/k_{\mathrm{HAA}}^{\mathrm{ZCT}\_0}$ M^−1^s^−1^/ s^−1^		*ΔG_a_* kJ mol^−1^	$k_{\mathrm{RAF}}^{\mathrm{ZCT}\_0\;}$$/k_{\mathrm{HAA}}^{\mathrm{ZCT}\_0}$ M^−1^s^−1^/ s^−1^	
**C-3**	44	4.11 × 10^6^		40	2.12 × 10^7^	
**C-5**	55	5.77 × 10^4^		55	5.00 × 10^4^	
**C-6**	57	2.61 × 10^4^		51	2.46 × 10^5^	
**C-7**	55	4.45 × 10^4^		57	2.05 × 10^4^	
**C-8**	49	4.64 × 10^5^		46	1.54 × 10^6^	
**C-9**	57	2.68 × 10^4^		54	7.97 × 10^4^	
**C-10**	49	4.43 × 10^5^		45	2.24 × 10^6^	
**C-1′**	51	2.19 × 10^5^		52	1.57 × 10^5^	
**C-1″**	39	2.84 × 10^7^		36	4.37 × 10^7^	
**C-2″**	42/ 104	9.14 × 10^6^/ 1.05 × 10^−5^		39	2.69 × 10^7^	
**C-3″**	34	7.86 × 10^7^		36/ 108	1.49 × 10^7^/ 5.45 × 10^−5^	
**C-4″**	34/104	1.40 × 10^7^/ 4.86 × 10^−6^		33	4.58 × 10^7^	
**C-5″**	42	7.59 × 10^6^		44/99	3.28 × 10^6^/ 7.75 × 10^−5^	
**C-6″**	32	5.34 × 10^7^		33	3.30 × 10^7^	

## Data Availability

Data is contained within the article and Supplementary Material.

## Supplementary Materials

The following are available online at https://www.mdpi.com/article/10.3390/ijms222413273/s1.
