# Supplementary material for: Mechanism of Antiradical Activity of Newly Synthesized 4,7-Dihydroxycoumarin Derivatives-Experimental and Kinetic DFT Study
Source: Int J Mol Sci. 2021 Dec 9;22(24):13273. doi: 10.3390/ijms222413273 (PMC8709309; doi:10.3390/ijms222413273)
Supplement: Supplementary file 1 [file ijms-22-13273-s001.zip › SuppMaterialIJMSRew.pdf]

Supplementary Material

Mechanism of antiradical activity of newly synthesized 4,7-dihydroxycoumarin derivatives-experimental and kinetic DFT study

Žiko Milanović[1,2], Dušan Dimić[3], Milan Žižić[4], Dejan Milenković[1], Edina Avdović[1*], Zoran S. Marković[1*]

[a] *University of Kragujevac, Institute for Information Technologies, Department of Science, Jovana Cvijića bb, 34000 Kragujevac, Serbia*

[b]*University of Kragujevac, Faculty of Science, Department of Chemistry, Radoja Domanovića 12, 34000 Kragujevac, Serbia*

[c] *Faculty of Physical Chemistry, University of Belgrade, 12-16 Studentski trg, 11000 Belgrade, Serbia*

[d] *University of Belgrade, Institute for Multidisciplinary Research, Life Sciences Department, Kneza Višeslava 1, 11030 Belgrade, Serbia*

***Corresponding author's e-mail address:** edina.avadovic@pmf.kg.ac.rs, zmarkovic@uni.kg.ac.rs

Corresponding author. Tel.: +381-34-610-01-95.

To quantify the molar ratios of acid-base species, the relationship between the acid constants (K_a) and the pK$_a$ values (eq.1s) as well as the expression for the equilibrium constant of the deprotonation process (eq.2s) is was used:

$$K_a = 10^{-pKa} \tag{1s}$$

$$K_a = \frac{\left[A-O^-\right]\left[H^+\right]}{\left[A-OH\right]} \tag{2s}$$

Molar fractions (*f*) of the represented acid-base species, **A-3OH**, **A-3O$^-$**, **A-4OH**, **A-4O$^-$** were calculated using the following equations:

$$f(A-O^-) = \frac{1}{1+\beta\left[H^+\right]} \tag{3s}$$

$$f(A-OH) = \beta\left[H^+\right] f(A-O^-) \tag{4s}$$

where [H$^+$] represents the concentration of hydrogen ions at physiological pH in this case ([H$^+$]=3.98×10^{-8} M, while β represents global formation equilibrium constants:

$$\beta = 10^{pKa} \tag{5s}$$

The overall rate constants for the mechanism between investigated compounds and HO$^\bullet$ was calculated following equation:

$$k_{overall} = f(A-OH)\times k_{TOT}^{A-OH} + f(A-O^-)\times k_{TOT}^{A-O^-} \tag{6s}$$

Due to the very small molar fraction of the anion at physiological pH, eq. 6s can be safely approximated to:

$$k_{overall} = f(A\!-\!OH) \times k_{tot}^{A-OH} \tag{7s}$$

where k_{tot}^{A-OH} represent the sum of the favorable reaction pathways for the neutral species and $HO^{\bullet}$:

$$k_{tot}^{A-OH} = k_{HAT} + k_{RAF} + k_{SPL} + k_{ET} \tag{8s}$$

while *f*(A–OH) represents the corresponding molar fractions of **A-3OH** and **A-4OH** species.

Antioxidative capacity r^T of the examined antioxidant relative to trolox (**Tx**) was calculated using the formula:

$$r^T = \frac{k_{overall}}{k_{overall}^{Trolox}} \tag{9s}$$

The relative amounts of products (%), i.e. branching ratios (Γi) were determined:

$$\Gamma_i = \frac{k_i}{k_{overall}} \tag{10s}$$

to evaluate which of the mechanistic pathways, i, is dominant.

Table S1. The experimental and calculated chemical shifts (ppm) in the ^1H NMR septrum of investigated compounds

^1H NMR Compound	Experimental		Theoretical	
	**A-3OH**	**A-4OH**	**A-3OH**	**A-4OH**
**C4'(CH$_3$)-3H**	2.31	2.31	2.36	2.33
**C2'(CH$_3$)-3H**	2.58	2.60	2.70	2.65
**-OCH$_3$-3H**	3.82	3.36	4.11	4.09
**C6"-H**	6.81	7.03	7.09	7.06
**C8-H**	7.10	7.13	7.14	7.16
**C2"-H**	6.81	7.03	7.00	6.83
**C6-H**	7.10	7.13	7.25	7.34
**C4"-H**	/	/	/	/
**C3"-H**	/	/	/	/

**C5”-H**	7.10	6.86	7.19	7.24
**C5-H**	8.01	8.01	8.42	8.45
**N1-H**	15.24	15.25	15.45	15.38
**AAE**	/	/	**0.18**	**0.06**
**R**	/	/	**0.999**	**0.999**

Table S2. The experimental and calculated chemical shifts (ppm) in the ^{13}C NMR spectrum of investigated compounds

^{13}C NMR Compound	Experimental		Theoretical	
	**A-3OH**	**A-4OH**	**A-3OH**	**A-4OH**
**C4’ (CH_3)**	20.54	20.62	16.96	16.90
**C2’(CH_3)**	21.06	21.06	17.47	18.28
**-O<u>C</u>H_3**	55.97	56.05	52.07	50.95
**C3**	96.76	96.70	97.80	97.88
**C2”**	109.85	109.87	108.50	107.92
**C8**	112.52	110.10	109.11	108.49
**C5”**	112.78	117.71	111.34	113.42
**C10**	116.34	115.62	116.53	117.21
**C6”**	117.68	118.02	117.28	117.84
**C6**	118.05	118.07	117.52	117.98
**C5**	127.11	127.10	126.51	126.76
**C1”**	128.63	127.14	130.18	129.43
**C4”**	147.21	146.21	146.91	147.08
**C3”**	147.71	148.21	147.21	147.31
**C7**	153.82	153.83	157.19	156.90
**C9**	154.85	154.85	158.00	157.82
**C2**	161.44	161.48	161.98	161.93
**C1’**	168.65	168.66	172.36	172.56
**C3’**	175.67	175.82	176.91	177.60
**C4**	179.54	179.51	181.11	181.04
**AAE**	/	/	**1.79**	**2.03**
**R**	/	/	**0.999**	**0.999**

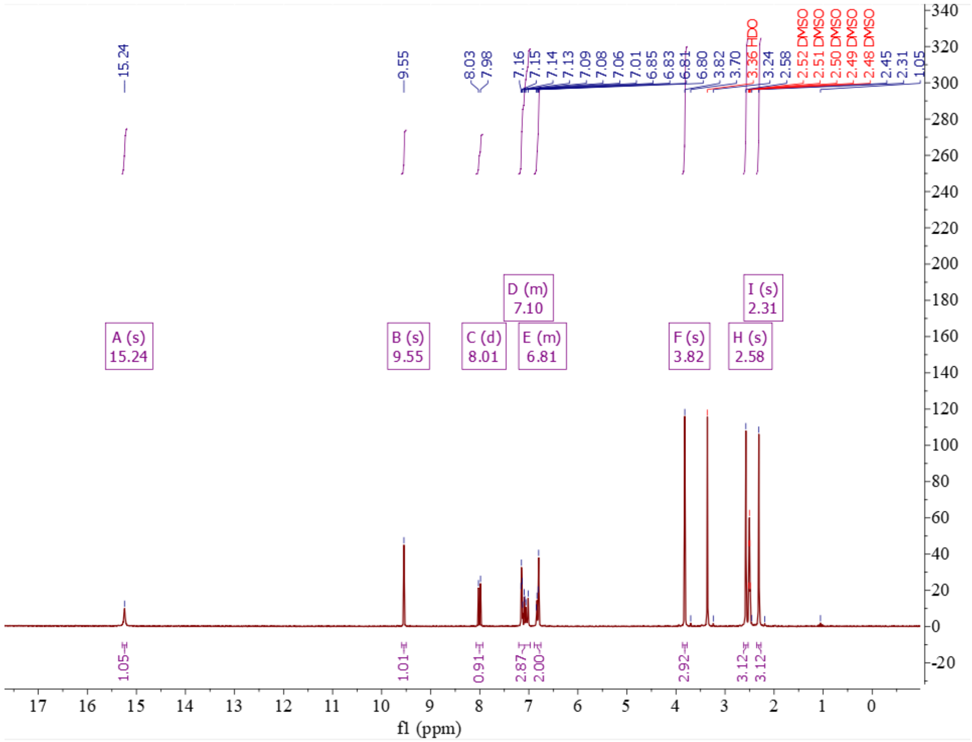

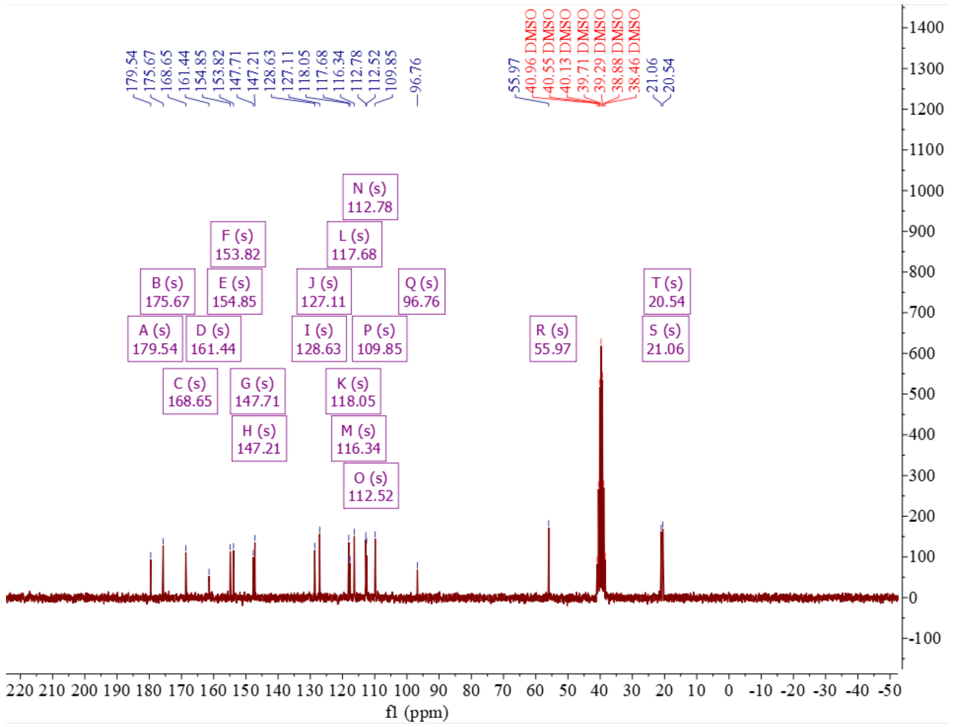

Figure S1. ^{1}H NMR spectrum of **A-3OH** (up) and **A-4OH** (down) in DMSO-d*6* at 25 °C

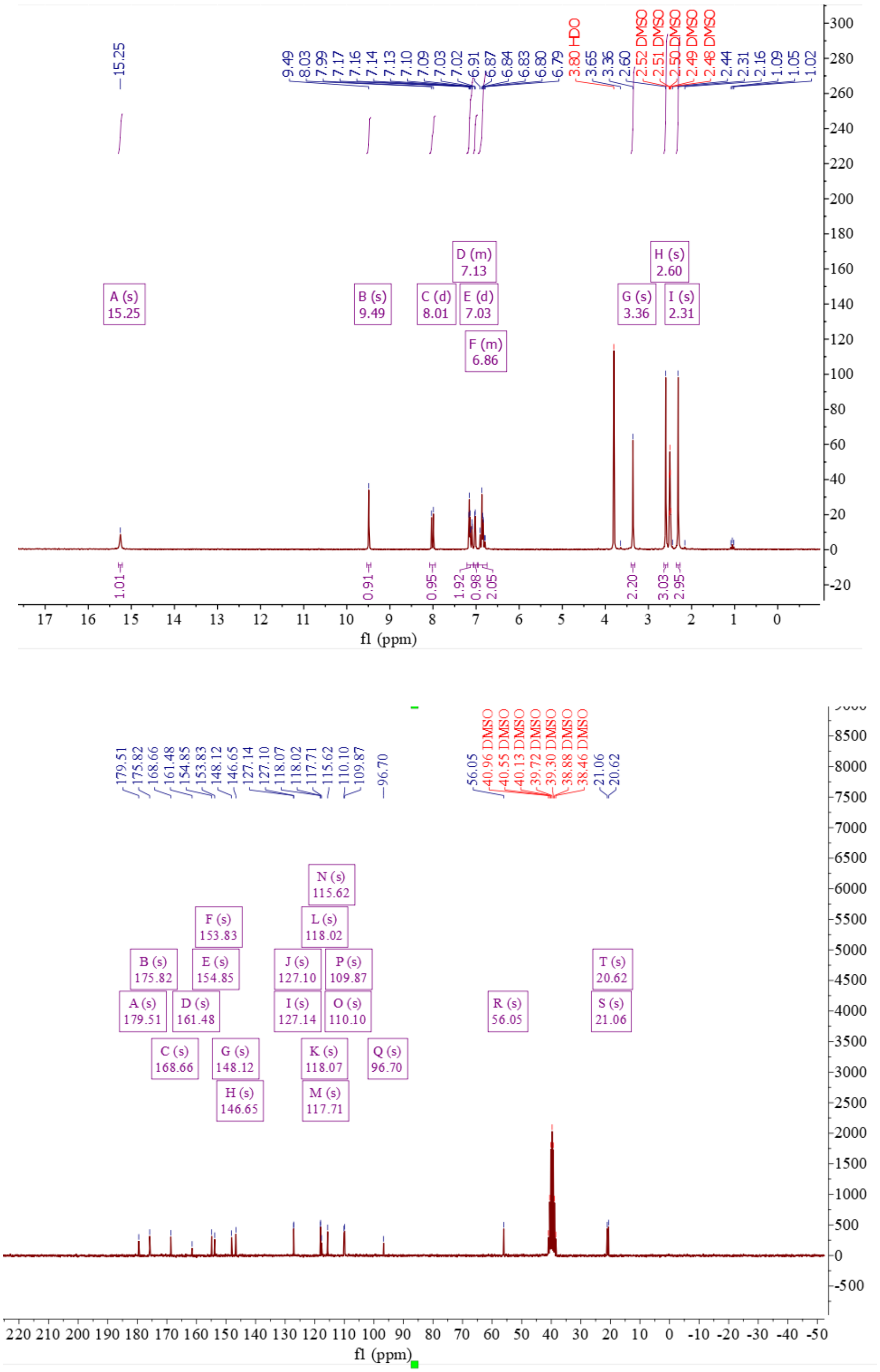

Figure S2. ^{13}C NMR spectrum of **A-3OH** (up) and **A-4OH** (down) in DMSO-d*6* at 25 °C

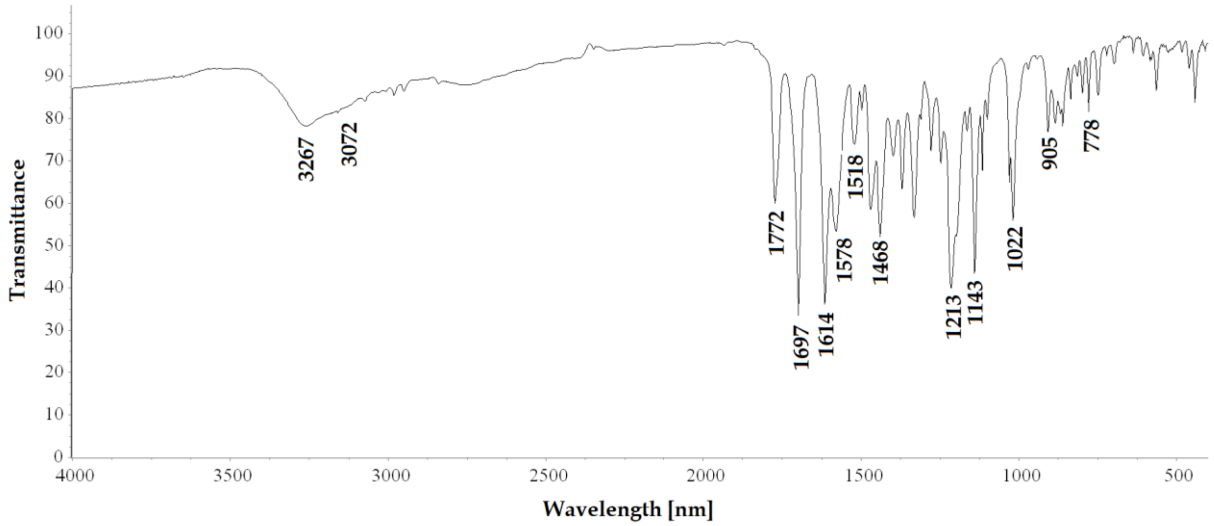

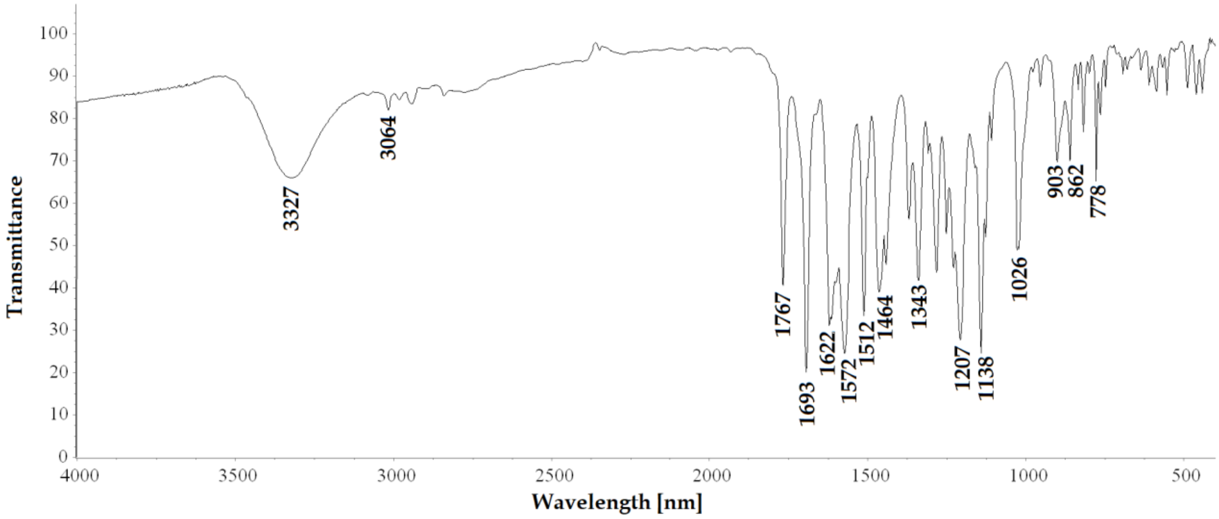

Figure S3. FT-IR spectrum of **A-3OH** (up) and **A-4OH** (down)

Table S3. Estimated molar fraction (%) of acid-base forms of the investigated compounds at different pH values

pH	A-3OH	A-3O⁻	A-4OH	A-4O⁻
1.0	100.0	0.0	100.0	0.0
2.0	100.0	0.0	100.0	0.0
3.0	100.0	0.0	100.0	0.0
4.0	100.0	0.0	100.0	0.0

5.0	100.0	0.0	100.0	0.0
6.0	100.0	0.0	100.0	0.0
7.0	99.8	0.2	100.0	0.0
**7.4**	**99.4**	**0.3**	**99.9**	**0.1**
8.0	97.8	2.2	99.6	0.4
9.0	81.7	18.3	96.6	3.4
10.0	30.9	69.1	73.8	26.2
11.0	4.3	95.7	22.0	78.0
12.0	0.4	99.6	2.7	97.3
13.0	0.0	100.0	0.3	99.7
14.0	0.0	100.0	0.0	100.0

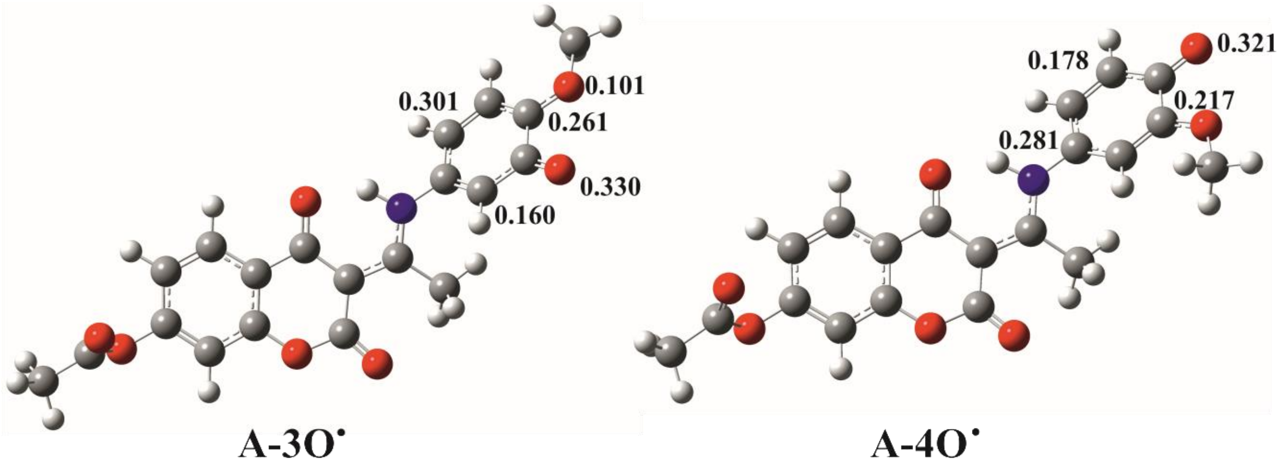

A-3O• **A-4O•**

Figure S4. NBO spin distribution for formed radical species

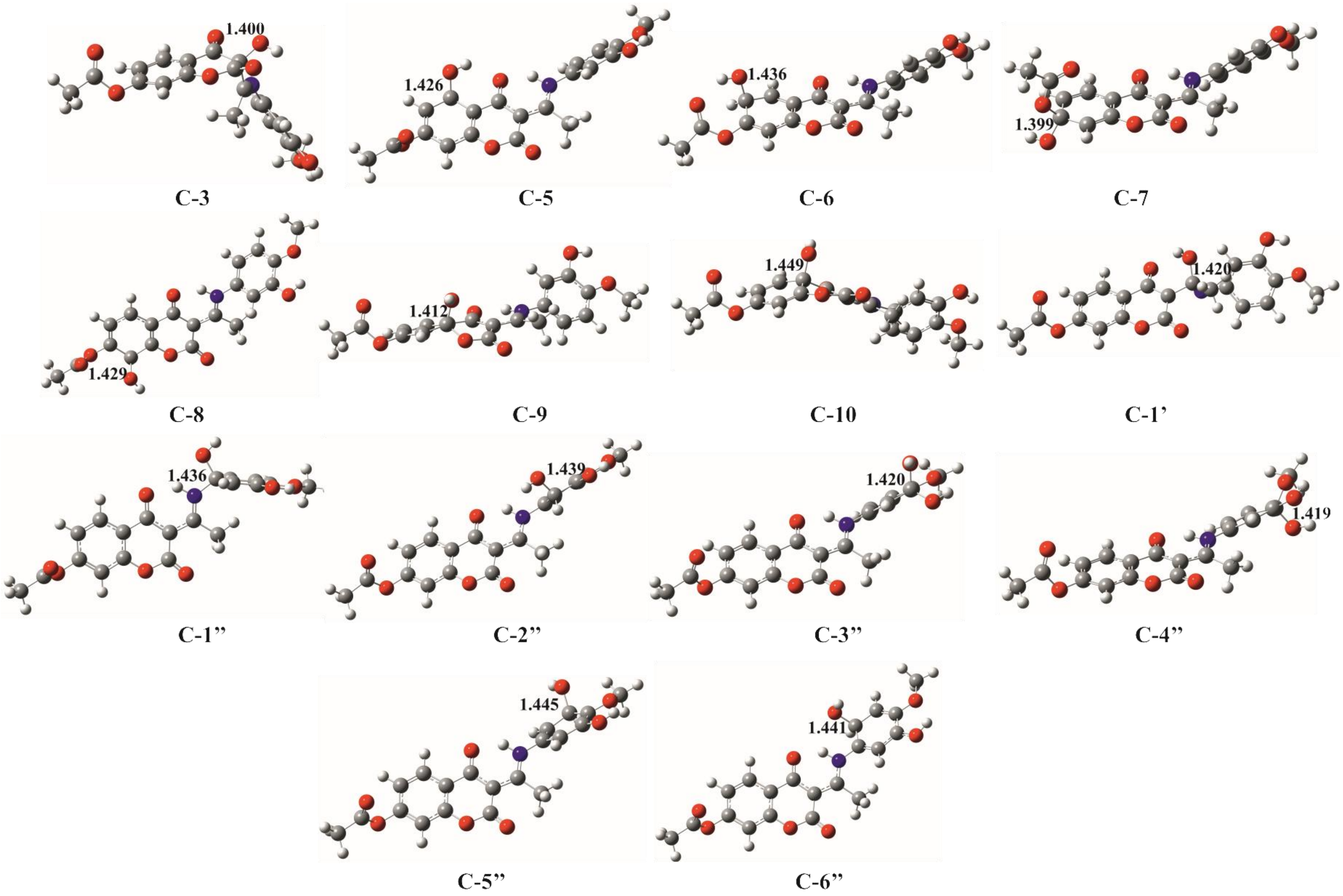

Figure S5. Optimized geometries of formed radical adducts between **A-3OH** and HO$^{\bullet}$ at M06-2X/6-311++G(d,p) level of theory with characteristic bond distances

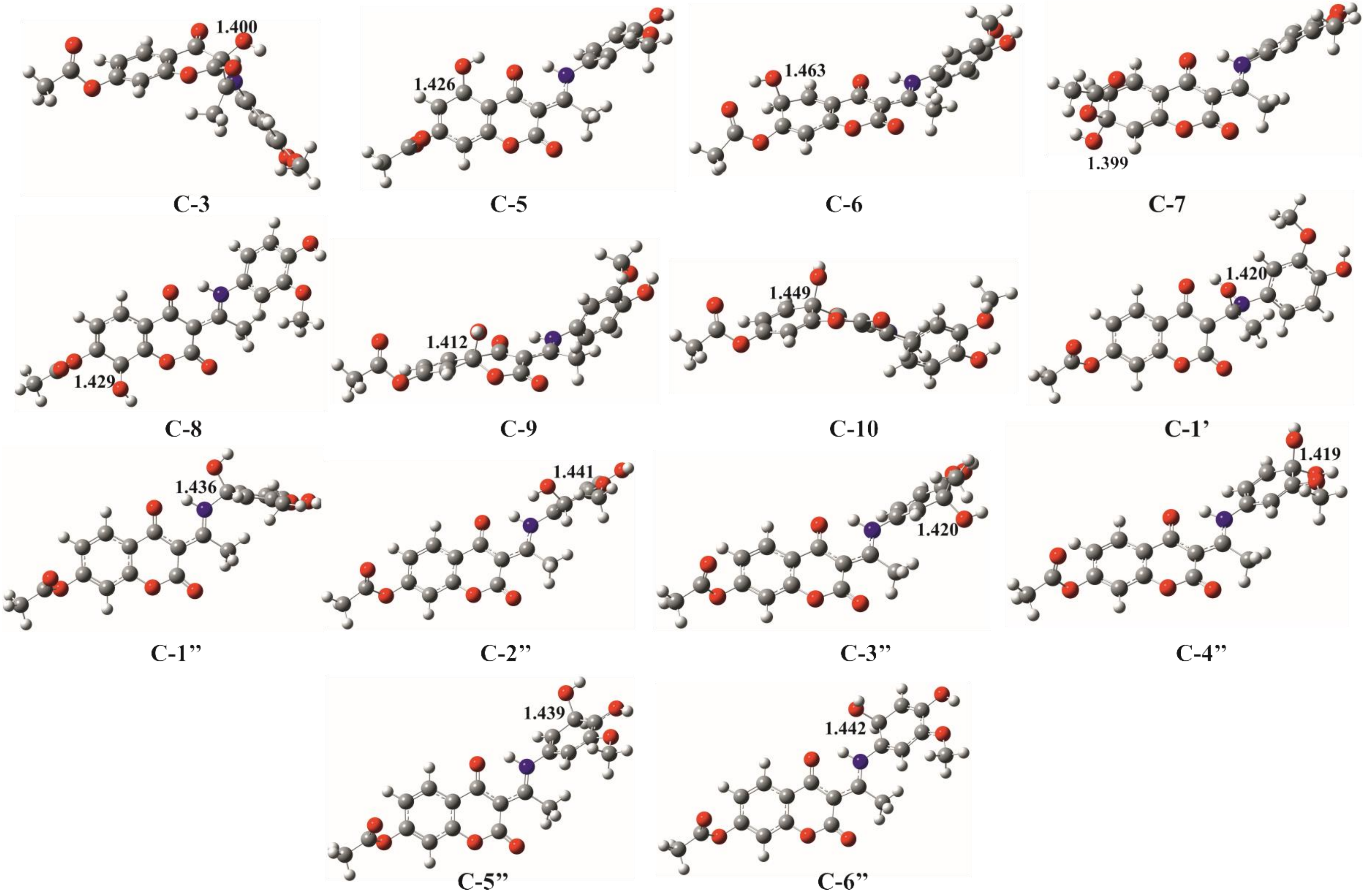

Figure S6. Optimized geometries of formed radical adducts between **A-4OH** and HO• at M06-2X/6-311++G(d,p) level of theory with characteristic bond distance

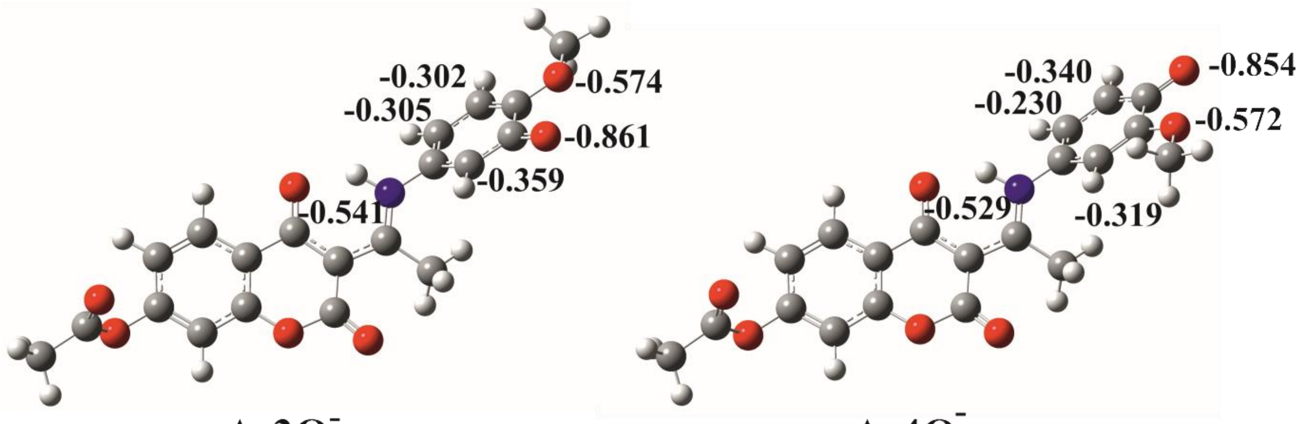

A-3O$^-$ A-4O$^-$

Figure S7. NBO charge distribution for formed anion specie

Table S4. Estimated kinetic parameters: activation energies (ΔG_a, kJ mol^{-1})**,** reaction rate constants (M^{-1}s^{-1} for all reaction patways and s^{-1} for HAA) estimated by the conventional transition state theory (k_{TST})

Position	RAF/HAA			
	**A-3OH**		**A-4OH**	
	ΔG_a	$k_{RAF}^{TST}/k_{HAA}^{TST}$	ΔG_a	$k_{RAF}^{TST}/k_{HAA}^{TST}$
**C-3**	44	3.19×10^{6}	40	1.67×10^{7}
**C-5**	55	3.67×10^{4}	55	3.16×10^{4}
**C-6**	57	1.76×10^{4}	51	1.65×10^{5}
**C-7**	55	2.93×10^{4}	57	1.35×10^{4}
**C-8**	49	3.52×10^{5}	46	1.17×10^{6}
**C-9**	57	1.72×10^{4}	54	5.13×10^{4}
**C-10**	49	3.36×10^{5}	45	1.71×10^{6}
**C-1'**	51	1.74×10^{5}	52	1.25×10^{5}
**C-1''**	39	2.52×10^{7}	36	6.17×10^{7}
**C-2''**	42/ 104	7.50×10^{6}/ 3.30×10^{-6}	39	2.32×10^{7}
**C-3''**	34	1.51×10^{8}	36/ 108	1.25×10^{8}/ 4.11×10^{-5}
**C-4''**	34/ 104	1.41×10^{8}/ 3.59×10^{-6}	33	2.44×10^{8}
**C-5''**	42	6.05×10^{6}	44/ 99	2.56×10^{6}/ 3.15×10^{-5}
**C-6''**	32	3.48×10^{8}	33	2.47×10^{8}

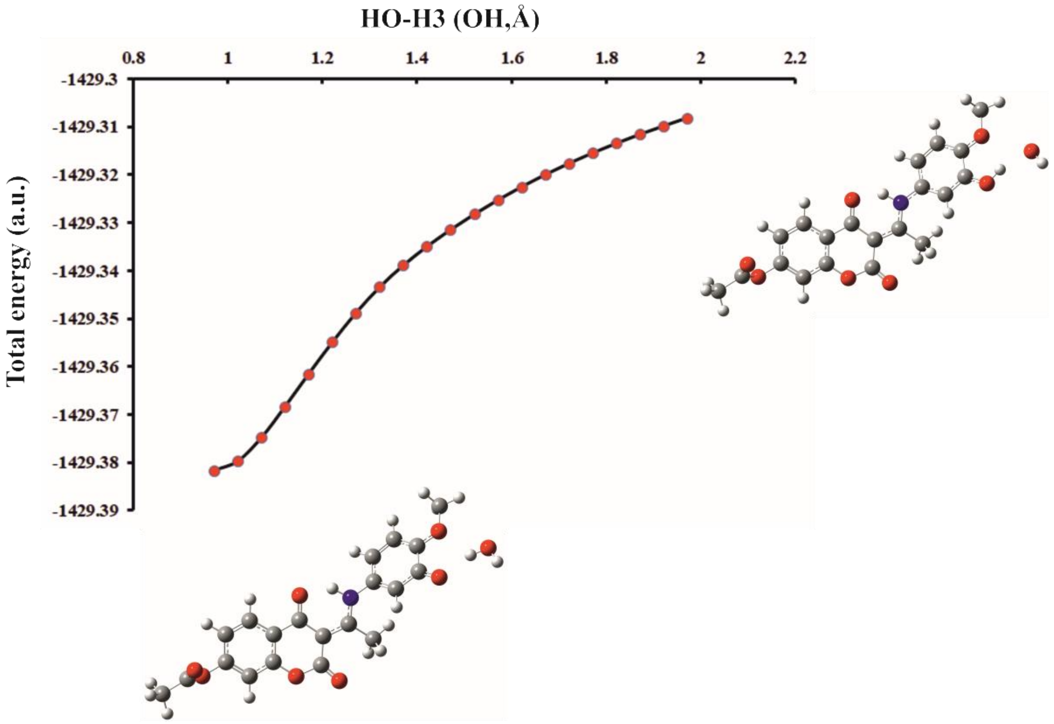

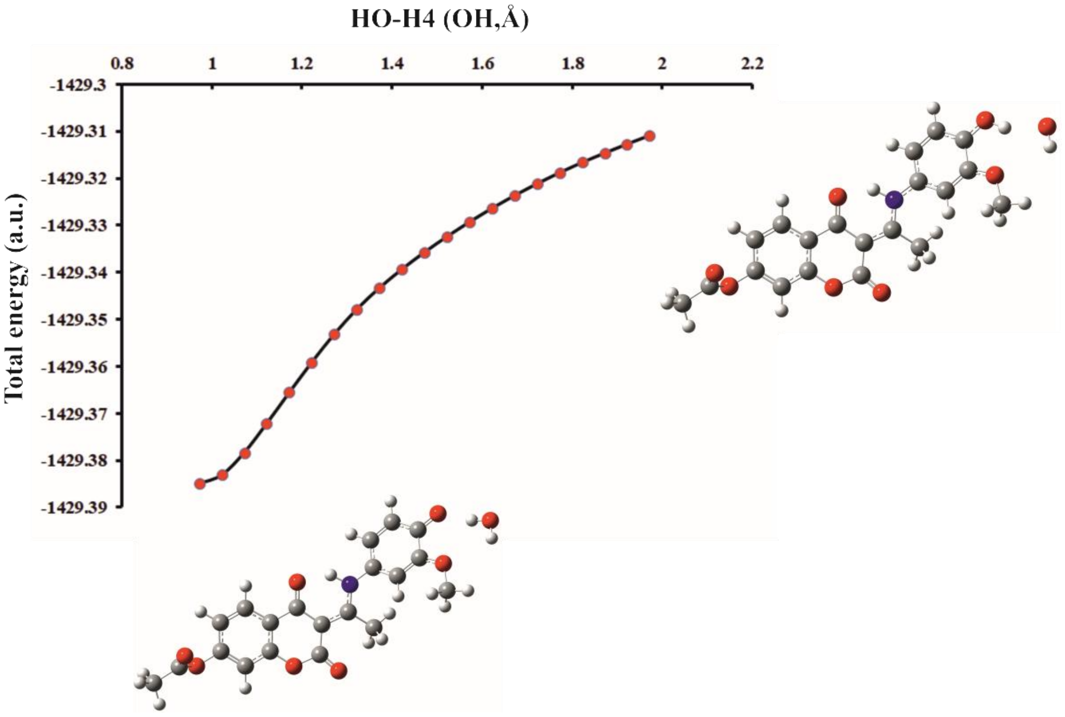

Figure S8. Dependence of total energy (au) on the characteristic HO–H3 (**A-3OH**,up) and HO–H4 (**A-4OH**, down) distance (Å) for HAT mechanisam

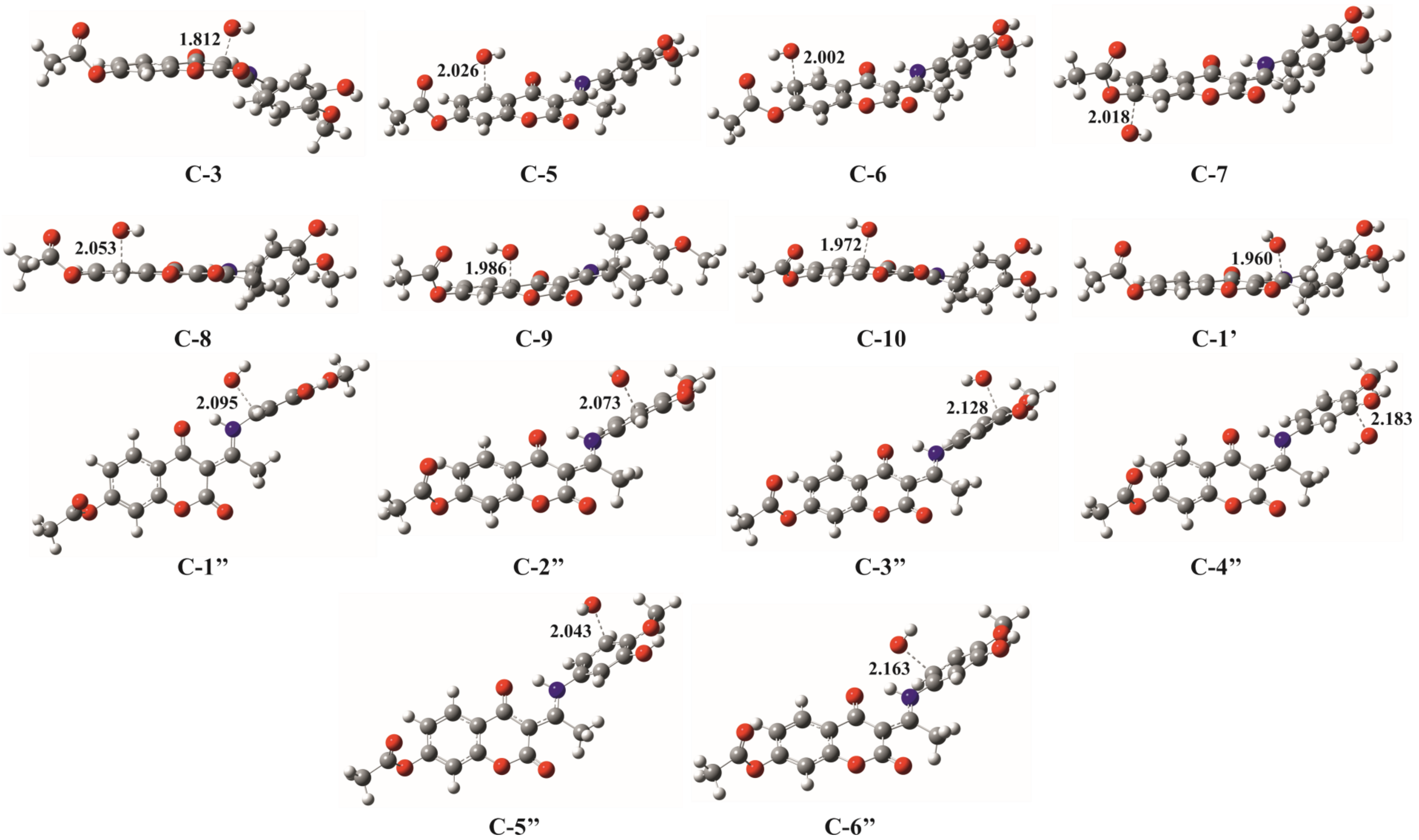

Figure S9. Optimized transition state geometries for the formation of radical adducts of **A-3OH** compounds in water at M06-2X/6-311++G(d,p) level of theory

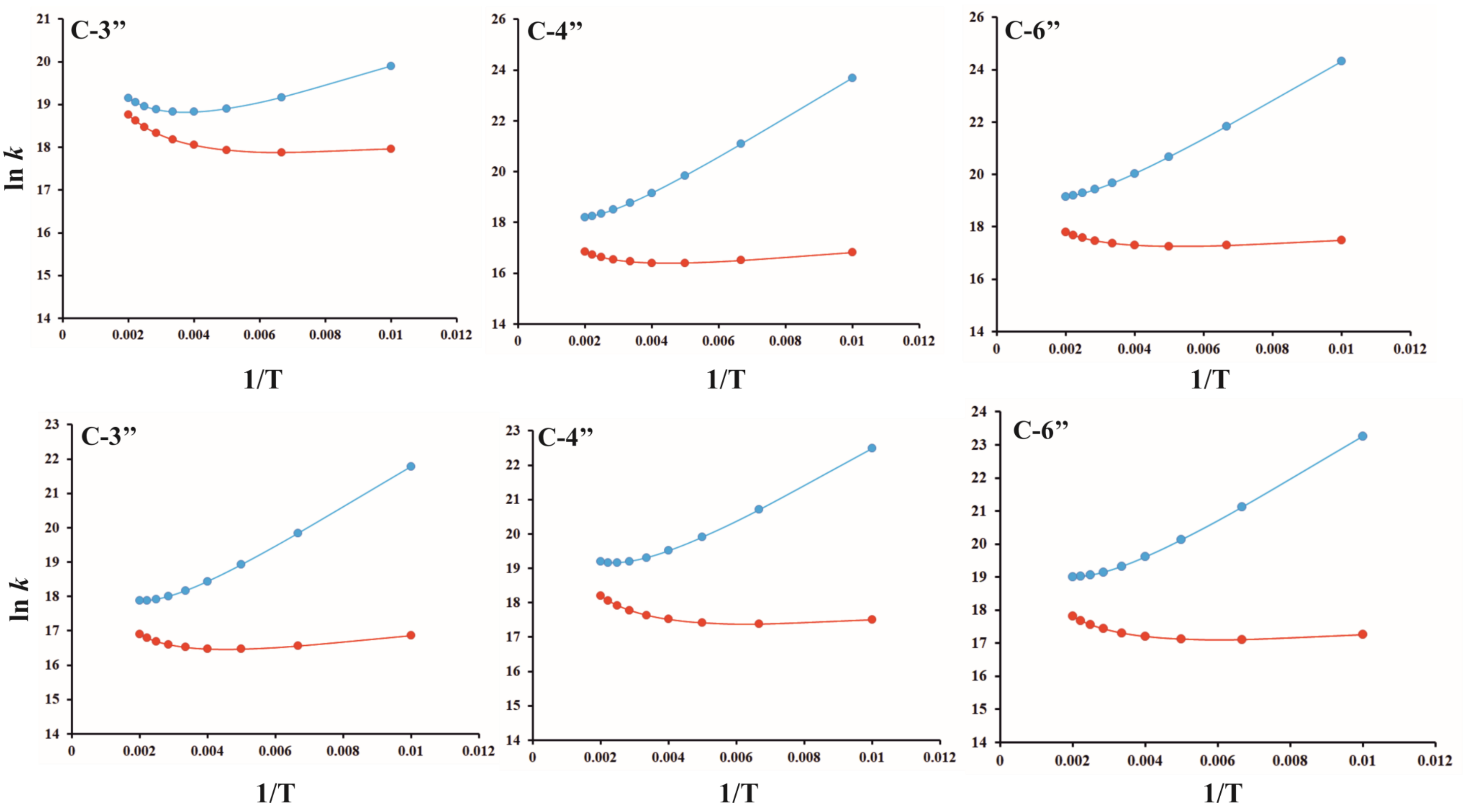

Figure S10. Graphs of dependence of lnk_{TST} (blue line) and lnk_{ZCT_0} (red line) on reciprocal temperature for RAF mechanism (**A-3OH** (up), **A-4OH** (down))

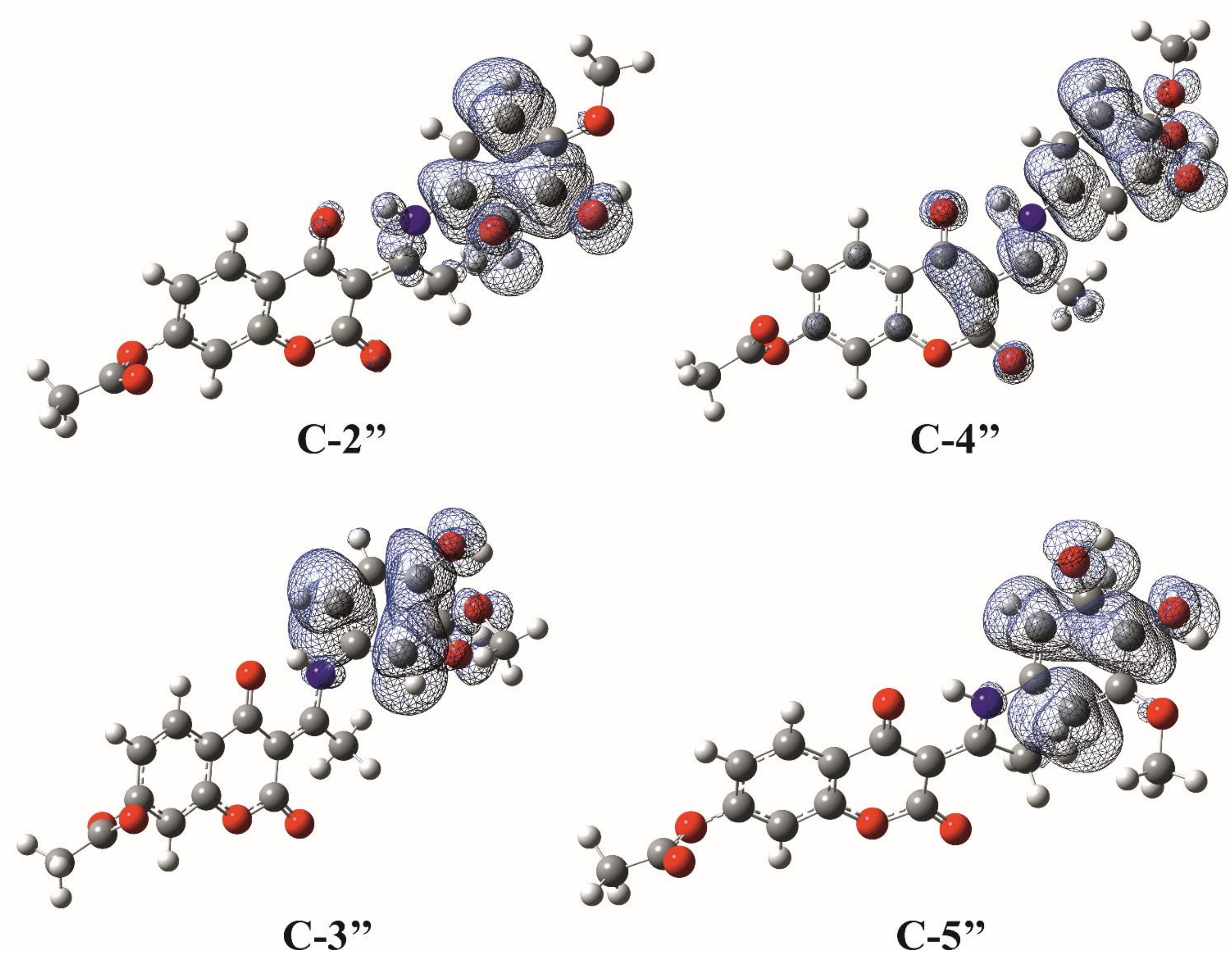

Figure S11. Spin density distribution maps (0.002 electron/bohr3) for formed radical adducts [**HO-A3OH**]• (up) and [**HO-A4OH**]• (down). The blue color represents the positive spin density

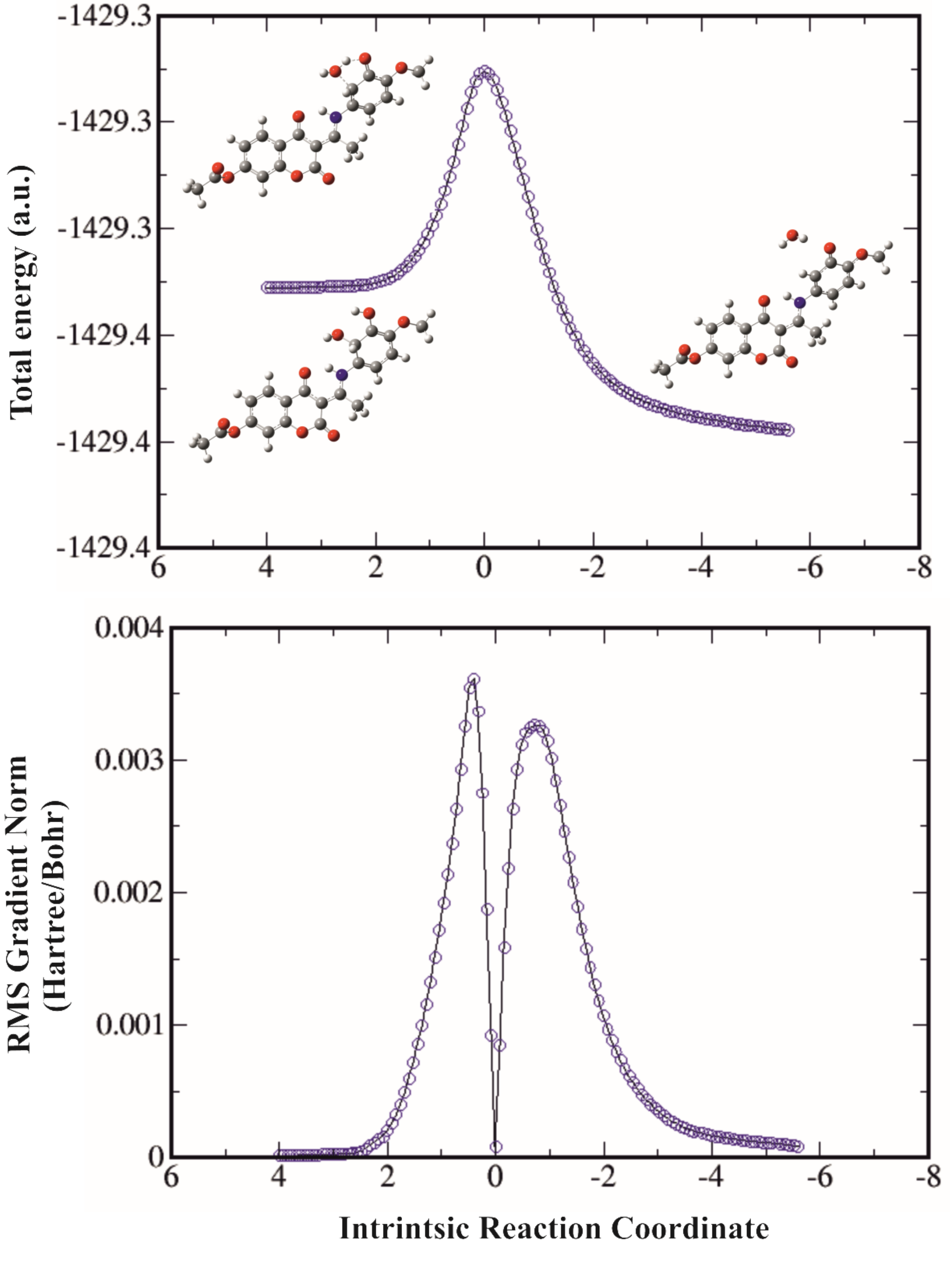

Figure S12. Results of the IRC calculation with reaction participants and RMS Gradient Norm for transition state at C2'' position (**A-3OH**) involved in the *i*HAA mechanism

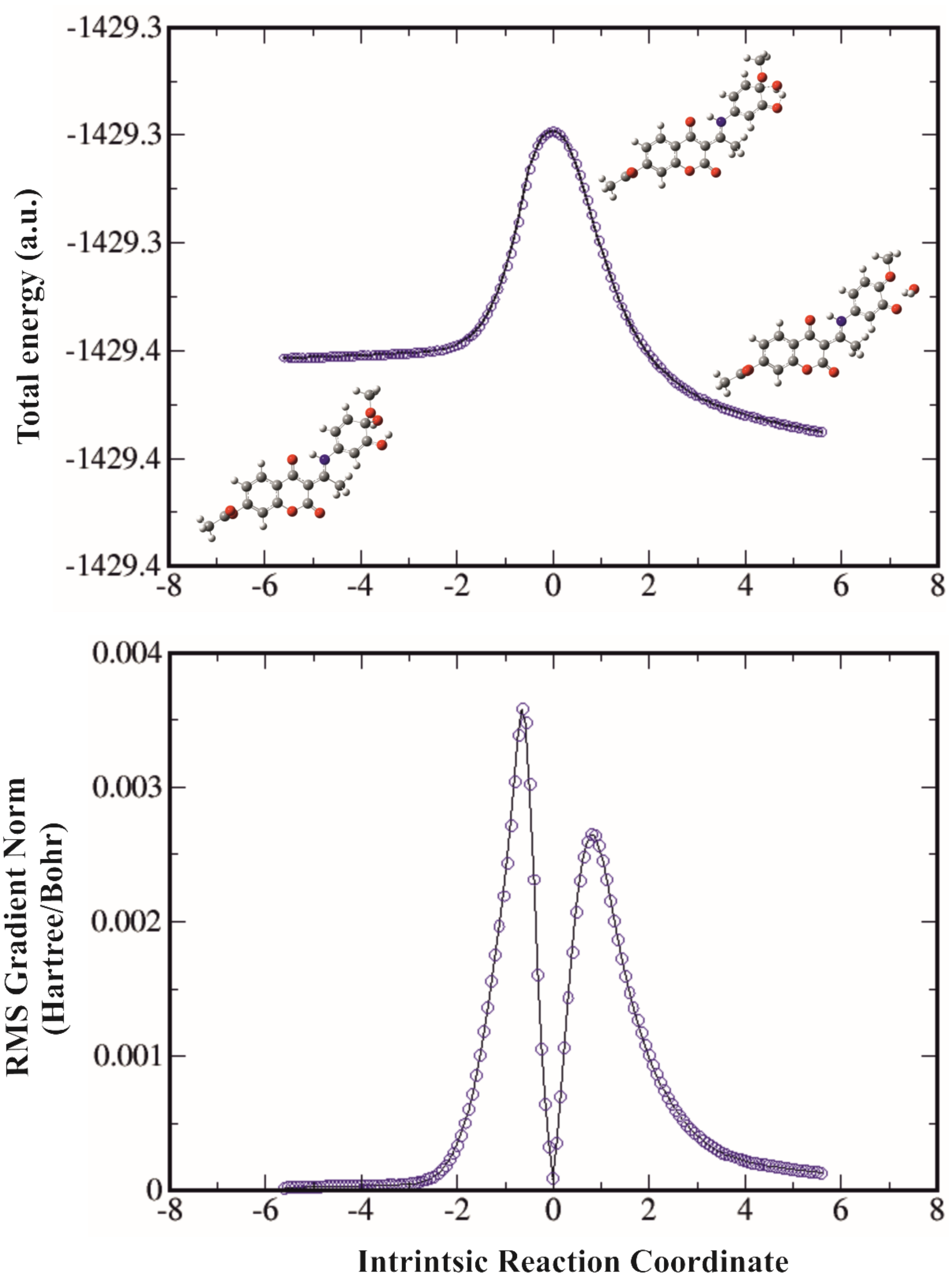

Figure S13. Results of the IRC calculation with reaction participants and RMS Gradient Norm for transition state at C4’’ position (**A-3OH**) involved in the *i*HAA mechanism

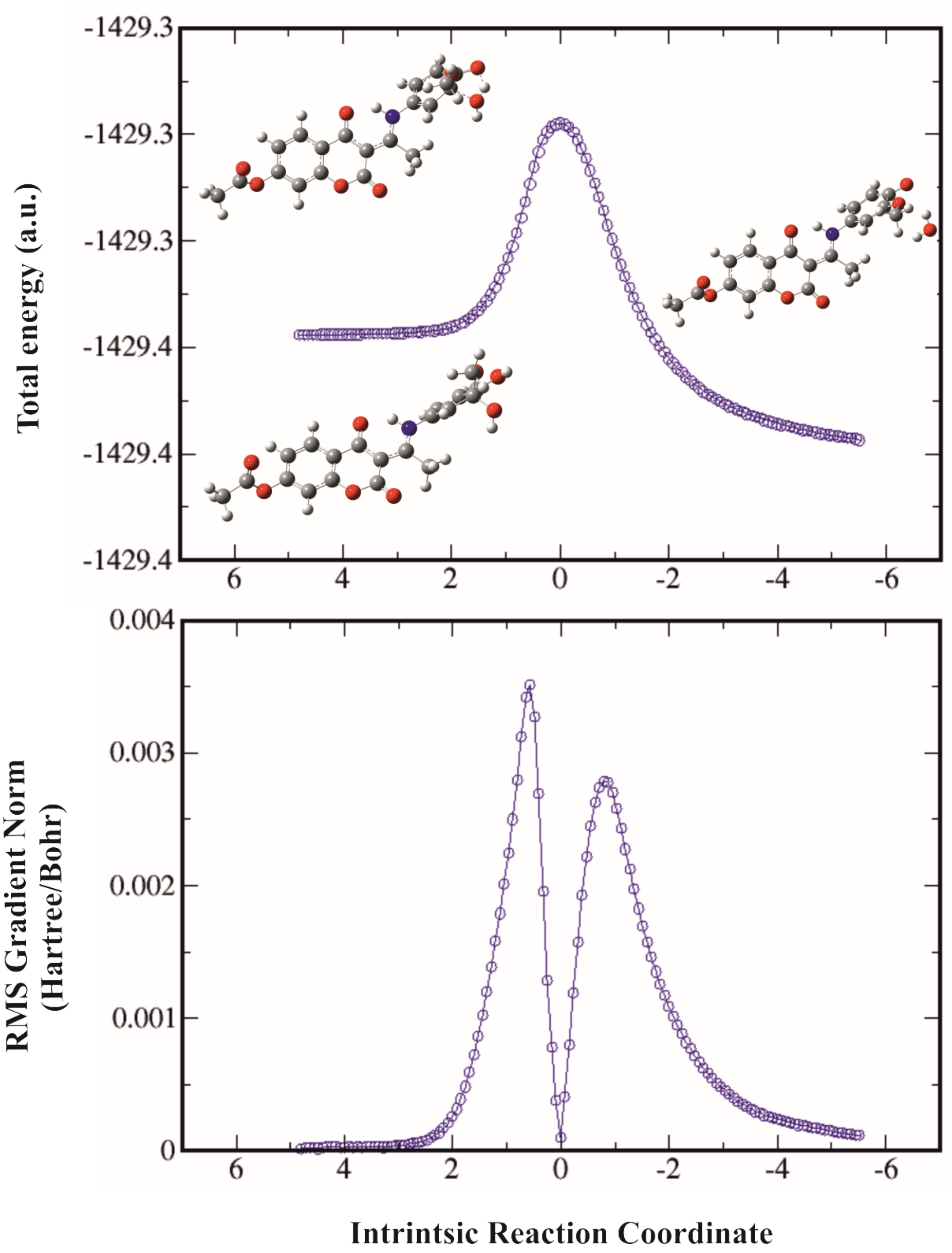

Figure S14. Results of the IRC calculation with reaction participants and RMS Gradient Norm for transition state at C3'' position (**A-4OH**) involved in the *i*HAA mechanism

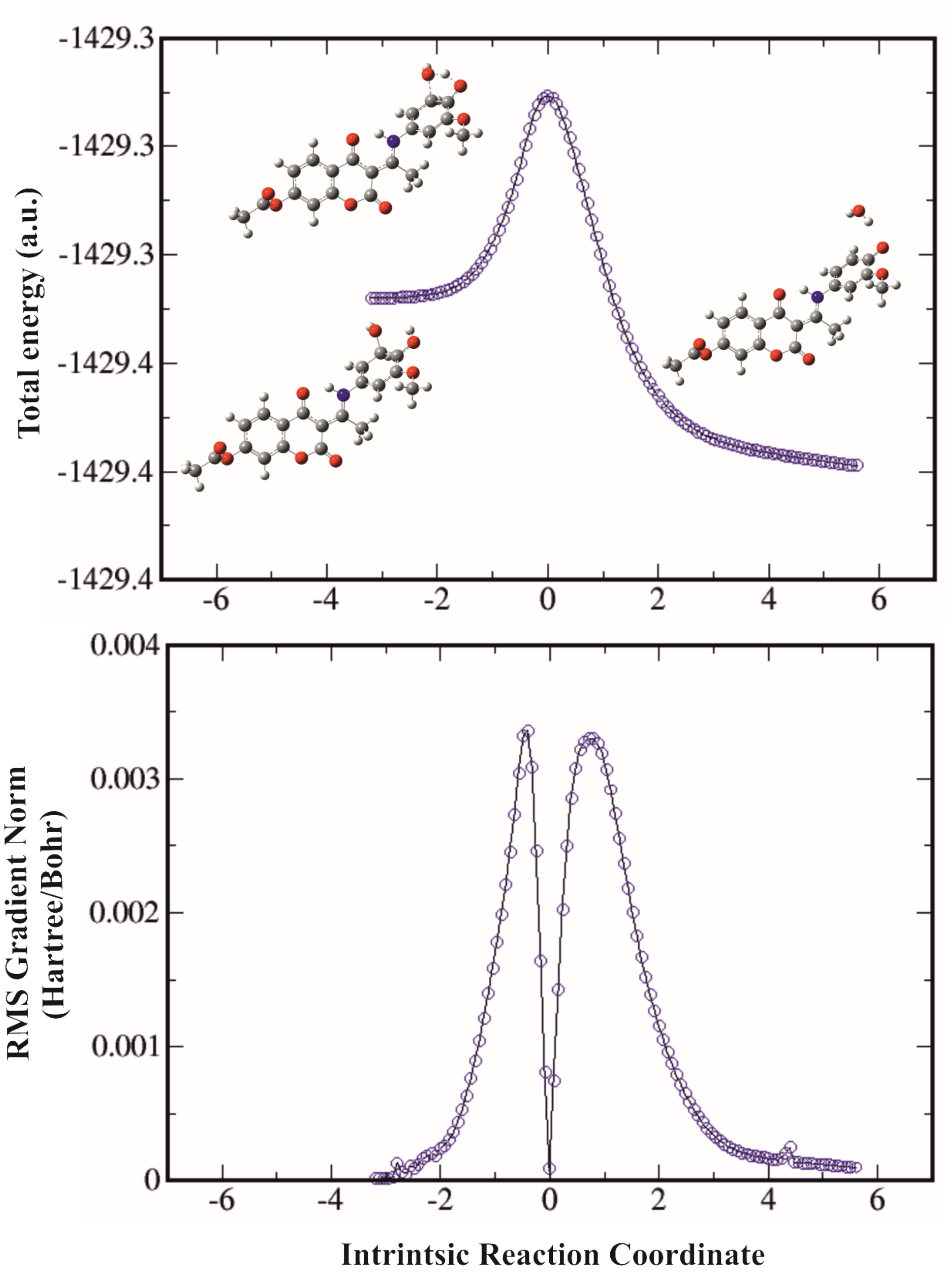

Figure S15. Results of the IRC calculation with reaction participants and RMS Gradient Norm for transition state at C5'' position (**A-4OH**) involved in the *i*HAA mechanism

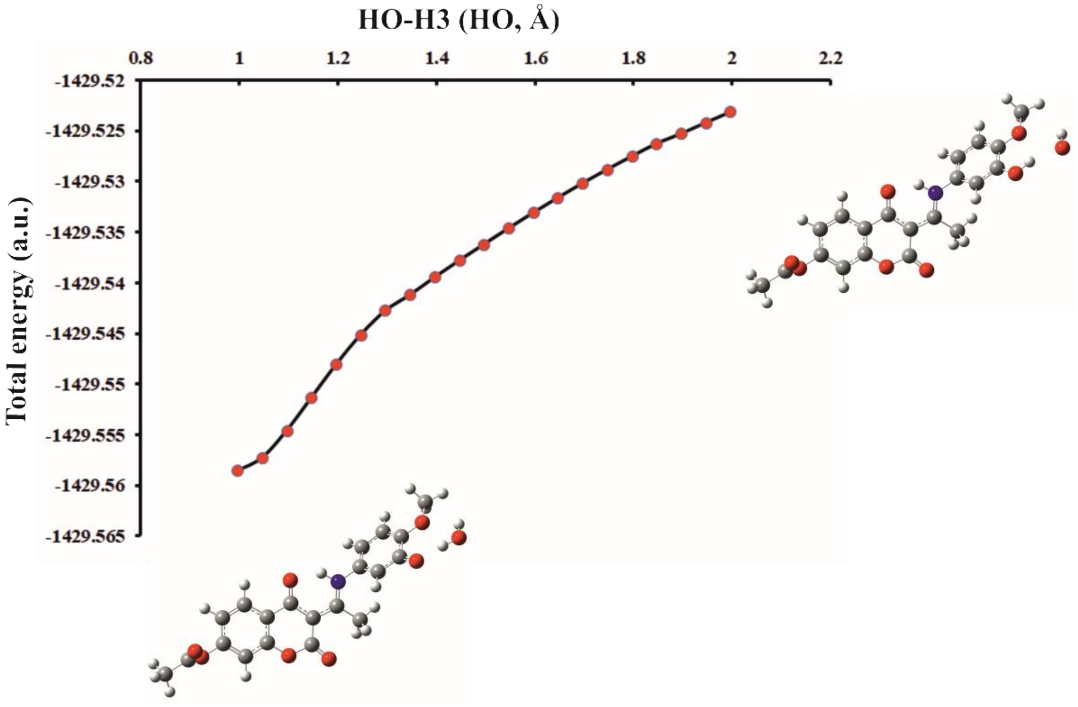

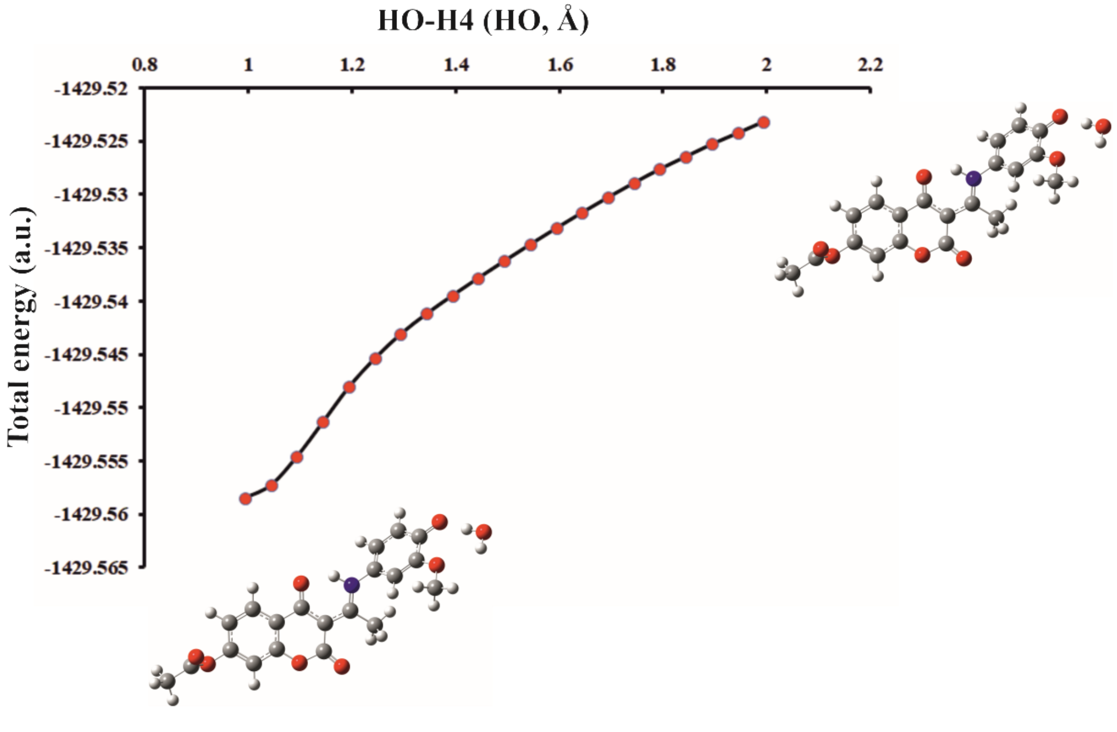

Figure S16. Dependence of total energy (au) on the characteristic HO–H3 (**A-3OH**,up) and HO–H4 (**A-4OH**, down) distance (Å) for SPL mechanisam.

Table S5 . Relative amount of products (%), i.e. branching ratios (Γ_i) and overall rate constants ($k_{overall}$) estimated at pH=7.4

Mechanisam	Position	$\Gamma_{A\text{-}3OH}$ (%)	$\Gamma_{A\text{-}4OH}$ (%)
HAT	**3-OH/4-OH**	35.39	19.34
RAF	**C-3**	0.08	0.21
	**C-5**	0.00	0.00
	**C-6**	0.00	0.00
	**C-7**	0.00	0.00
	**C-8**	0.01	0.02
	**C-9**	0.00	0.00
	**C-10**	0.01	0.02
	**C-1'**	0.00	0.00
	**C-1''**	0.53	0.44
	**C-2''**	0.17	0.27
	**C-3''**	1.46	0.40
	**C-4''**	0.28	0.46
	**C-5''**	0.14	0.03
	**C-6''**	0.99	0.33
	**C2''/C3''**	0.00	0.00
	**C4''/C5''**	0.00	0.00
SPLET	**3-OH/4-OH**	25.57	59.12
	**3-O⁻/4-O⁻**	35.39	19.34
$k_{overall}^{A-3OH}/k_{overall}^{A-4OH}$		**5.06×10⁹**	**9.49×10⁹**
r^T		**2.61**	**4.90**

Cartesian coordinates:

A-3OH

G= -1353.305040 au

0 1	x	y	z
C	1.00051600	2.03881400	-0.35108300
C	3.02032100	0.72825000	-0.33833100
C	2.33364500	-0.47105500	-0.20873400
C	0.86007000	-0.46454200	-0.16175800
C	0.21138500	0.82118800	-0.29341100
H	4.93478500	1.70776200	-0.50586300
C	4.41125500	0.76621600	-0.40304600
C	3.06020900	-1.66545800	-0.14116100
C	4.43954900	-1.65685600	-0.20541700
C	5.09377500	-0.43129000	-0.33671200
H	2.51334200	-2.59481100	-0.04286100
H	5.01572700	-2.57221700	-0.16092000
O	2.37558200	1.92216700	-0.39955400
O	0.59370500	3.17696500	-0.34965300
O	0.25451100	-1.53994200	-0.03163800
C	-1.21251600	0.91990300	-0.33375500
C	-1.93139800	2.19909400	-0.63808000
H	-1.91579600	2.86032200	0.23033400
H	-1.41775700	2.72257200	-1.44074200
H	-2.96168200	1.99745600	-0.92011600
O	6.47469600	-0.40086500	-0.46636200
C	7.22529500	-0.61304200	0.65228900
C	8.68367600	-0.57441500	0.32227100
H	8.93789100	0.41418000	-0.06292300
H	9.26400700	-0.78983300	1.21490200
H	8.89967900	-1.30418200	-0.45859600
O	6.73676900	-0.79164300	1.73236000
N	-1.93134500	-0.17487400	-0.14768700
H	-1.37596100	-1.03433200	-0.03409000
C	-3.35235100	-0.28109700	-0.07259500
C	-4.00167400	-1.19326600	-0.88829100
C	-4.06303800	0.47478300	0.86253400
C	-5.38581800	-1.34936400	-0.79039700
H	-3.43680000	-1.77766400	-1.60361200
C	-5.43363900	0.32817600	0.95134900
C	-6.10308400	-0.58945500	0.12086200
H	-5.88755300	-2.06140400	-1.43173100
H	-3.55838600	1.16746300	1.52532700
O	-6.13411600	1.06102500	1.85049400

H	-7.06731700	0.81855000	1.77908600
O	-7.44455100	-0.63459600	0.31966300
C	-8.20042500	-1.54717900	-0.46769400
H	-9.23410400	-1.43026700	-0.15410200
H	-8.10590900	-1.30748100	-1.52936400
H	-7.87203400	-2.57332100	-0.28644000

A-4OH

G= -1353.303999 au

0 1	x	y	z
C	0.79567600	1.87798500	-0.67758500
C	2.88637000	0.70535000	-0.44199400
C	2.26504200	-0.51083600	-0.19419800
C	0.79341200	-0.59104000	-0.20177300
C	0.07687400	0.62826200	-0.50404400
H	4.74766200	1.77570400	-0.65329600
C	4.27420000	0.82206100	-0.45958600
C	3.05574400	-1.64126800	0.04262400
C	4.43351700	-1.55476900	0.02766600
C	5.02145200	-0.31434600	-0.22524600
H	2.55915700	-2.58474400	0.23145600
H	5.05756900	-2.42135500	0.20420000
O	2.17649900	1.84093200	-0.66901700
O	0.32486900	2.98199900	-0.82114900
O	0.24585800	-1.68045800	0.02993100
C	-1.34794000	0.63299200	-0.60078600
C	-2.12626600	1.82372300	-1.07240000
H	-2.17580700	2.58177000	-0.28827800
H	-1.61648200	2.28022200	-1.91722000
H	-3.13356800	1.53161800	-1.35751700
O	6.40141600	-0.20785100	-0.31193900
C	7.13005900	-0.33716500	0.83381500
C	8.59373800	-0.24868700	0.53881300
H	8.80528200	0.68357400	0.01448600
H	9.15490600	-0.29743300	1.46761200
H	8.87735700	-1.07394000	-0.11602900
O	6.62182900	-0.49486500	1.90794100
N	-2.00927600	-0.47596800	-0.31165600
H	-1.40949900	-1.28068200	-0.08228700
C	-3.42390200	-0.66060500	-0.27021100
C	-3.98821400	-1.69995900	-0.99211800
C	-4.21021000	0.15887200	0.54625200
C	-5.36286900	-1.92192100	-0.91881800
H	-3.36315100	-2.32884700	-1.61403400

C	-5.57688300	-0.05858500	0.60436300
C	-6.15738700	-1.10679800	-0.13344700
H	-5.82822400	-2.72543200	-1.47630500
H	-3.74333600	0.94330100	1.12712200
O	-6.46130900	0.65896100	1.34083800
O	-7.49464500	-1.31796200	-0.06654500
H	-7.88213200	-0.65022600	0.51530800
C	-5.95823200	1.75548600	2.09515000
H	-5.24265700	1.40928400	2.84458900
H	-5.48536300	2.48782300	1.43617900
H	-6.81800900	2.20259100	2.58627900

A-3O$^{\bullet}$

G= -1352.671563 au

0 2	x	y	z
C	0.99596900	2.08194700	-0.31392300
C	2.98834200	0.73081400	-0.32917500
C	2.28046800	-0.45536400	-0.19104400
C	0.80936500	-0.42091100	-0.12640400
C	0.18200000	0.87819600	-0.25195000
H	4.91862800	1.67326000	-0.52092200
C	4.37844700	0.74188100	-0.41221400
C	2.98415400	-1.66394700	-0.13336100
C	4.36229700	-1.68169100	-0.21551900
C	5.03826700	-0.46886900	-0.35504000
H	2.42102100	-2.58279600	-0.02858700
H	4.92134200	-2.60790100	-0.17859300
O	2.36607600	1.93775700	-0.38041100
O	0.60971400	3.22623200	-0.29973500
O	0.18325200	-1.48340100	0.00907700
C	-1.23550300	1.00387200	-0.28406200
C	-1.93080500	2.29364000	-0.59500100
H	-1.92151300	2.95205300	0.27580600
H	-1.39538700	2.81144200	-1.38651700
H	-2.95854000	2.11024200	-0.89725800
O	6.41764300	-0.46613900	-0.50070400
C	7.17620800	-0.67020600	0.61449300
C	8.63150300	-0.66189100	0.26955700
H	8.89909500	0.31706200	-0.13097500
H	9.21690200	-0.87568700	1.15927300
H	8.82689200	-1.40539900	-0.50370100
O	6.69524900	-0.82083400	1.70213900
N	-1.96902000	-0.08657600	-0.09356800
H	-1.41359300	-0.95236200	0.00317200

C	-3.37991200	-0.19482000	0.00577200
C	-3.99788200	-1.25040000	-0.71674400
C	-4.11889100	0.62465400	0.80858400
C	-5.36246200	-1.46338900	-0.64851600
H	-3.38160400	-1.89114000	-1.33584900
C	-5.55146000	0.46888700	0.91214000
C	-6.15581700	-0.62824700	0.13782100
H	-5.79918700	-2.27535800	-1.21324600
H	-3.66410400	1.40697200	1.40375800
O	-6.24876200	1.20589400	1.62552400
O	-7.46614900	-0.72977700	0.27105200
C	-8.15097900	-1.76399800	-0.43987400
H	-9.20047300	-1.64826200	-0.18712200
H	-8.00890200	-1.64141900	-1.51492400
H	-7.79347700	-2.74348000	-0.11810900

A-4O˙

G= -1352.672948 au

0 2	x	y	z
C	0.74315500	1.96025800	-0.50947000
C	2.82768300	0.76489500	-0.37273600
C	2.20601200	-0.46648700	-0.21648600
C	0.73724800	-0.54569200	-0.22554100
C	0.01655100	0.69874000	-0.43028400
H	4.68760100	1.84737600	-0.51047400
C	4.21470600	0.88185100	-0.38714000
C	2.99689300	-1.61290800	-0.07149100
C	4.37400100	-1.52622800	-0.08565300
C	4.96247800	-0.26952900	-0.24357800
H	2.50119000	-2.56839100	0.04579500
H	4.99710300	-2.40493100	0.01804800
O	2.11966300	1.91734000	-0.50970400
O	0.26969800	3.06900600	-0.55958600
O	0.18591300	-1.64688200	-0.08498800
C	-1.39653300	0.71711300	-0.53033100
C	-2.16953500	1.93231100	-0.94398100
H	-2.26586300	2.63601000	-0.11455300
H	-1.62584400	2.45064400	-1.72982500
H	-3.15667200	1.65271400	-1.30311100
O	6.33983600	-0.15213900	-0.32946300
C	7.08573400	-0.43614400	0.77790400
C	8.54347400	-0.28613000	0.47959100
H	8.74680100	0.74961500	0.20299700
H	9.12391500	-0.56015700	1.35597500

H	8.81002400	-0.91763700	-0.36836800
O	6.59363100	-0.74518100	1.82568200
N	-2.05453500	-0.42932600	-0.33026200
H	-1.42677900	-1.24513900	-0.21610800
C	-3.43108500	-0.66632800	-0.23238800
C	-3.90316300	-1.90622400	-0.73737200
C	-4.29068400	0.22949000	0.41090800
C	-5.22375600	-2.22335800	-0.64449200
H	-3.19867800	-2.57981900	-1.21098200
C	-5.63786600	-0.07062300	0.51185300
C	-6.17960600	-1.32891900	-0.02862500
H	-5.60918800	-3.15688000	-1.03615200
H	-3.89024800	1.12802100	0.85614300
O	-6.54155300	0.70342500	1.09712200
O	-7.38737300	-1.59621900	0.05781600
C	-6.11839200	1.95202000	1.64483500
H	-5.39742100	1.79098900	2.44877600
H	-5.68129500	2.58006700	0.86540300
H	-7.01531300	2.41934000	2.04010900

A-3O^{-}

G= -1352.840565 au

-1 1	x	y	z
C	0.96904000	2.03635500	-0.34727600
C	2.98992100	0.72611500	-0.34000100
C	2.30021700	-0.47361600	-0.23328200
C	0.82505800	-0.46501000	-0.19270400
C	0.17993200	0.82090900	-0.31022200
H	4.90764400	1.70489200	-0.47729800
C	4.38172900	0.76274200	-0.39318200
C	3.02462000	-1.66977500	-0.17904800
C	4.40474200	-1.66278100	-0.23310100
C	5.06183300	-0.43666400	-0.33985100
H	2.47527700	-2.59942700	-0.09874500
H	4.97931100	-2.57959900	-0.19837000
O	2.34669800	1.92041400	-0.38870500
O	0.56643100	3.17708600	-0.33321300
O	0.21989500	-1.54349800	-0.07765800
C	-1.25065000	0.92414200	-0.34646000
C	-1.96085800	2.20791600	-0.65398200
H	-1.94707600	2.86934800	0.21398800
H	-1.44113700	2.72742200	-1.45555200
H	-2.99167600	2.00528600	-0.93288900
O	6.44462900	-0.40746600	-0.45694300

C	7.18375200	-0.62027900	0.66855900
C	8.64561300	-0.57826800	0.35404900
H	8.90247400	0.41282100	-0.02293700
H	9.21690700	-0.79751400	1.25156300
H	8.87104700	-1.30334400	-0.42847900
O	6.68493900	-0.80188700	1.74358100
N	-1.97351300	-0.16152600	-0.16009500
H	-1.42551100	-1.02339800	-0.05145400
C	-3.40170400	-0.26454700	-0.06778800
C	-4.05038400	-1.18331800	-0.87388200
C	-4.09083400	0.50056800	0.87195400
C	-5.44235000	-1.32032300	-0.73160400
H	-3.50332400	-1.77406900	-1.59733700
C	-5.49603100	0.40822300	1.04306800
C	-6.14713200	-0.56114500	0.18454500
H	-5.95518400	-2.03718500	-1.36028200
H	-3.54955300	1.19280300	1.50838800
O	-6.14709700	1.10679000	1.88739800
O	-7.49946800	-0.65429600	0.37094900
C	-8.20212400	-1.58245800	-0.43015100
H	-9.24717400	-1.51378500	-0.13480500
H	-8.10842900	-1.34027200	-1.49372200
H	-7.84354500	-2.60315500	-0.26251100

A-4O⁻

G= -1352.839240 au

-1 1	x	y	z
C	0.74502800	1.85203900	-0.65773900
C	2.84741400	0.69700900	-0.43779400
C	2.23364600	-0.52488100	-0.19872800
C	0.76119400	-0.61512100	-0.20961600
C	0.03912400	0.59668800	-0.50647600
H	4.70212800	1.78037300	-0.64474500
C	4.23493300	0.82231800	-0.45721600
C	3.03214600	-1.65148900	0.02858100
C	4.40978300	-1.55651900	0.01216600
C	4.98957000	-0.31105800	-0.23303800
H	2.54150200	-2.59942100	0.21073400
H	5.03978300	-2.42045200	0.18090700
O	2.12901800	1.82835900	-0.65282100
O	0.26713900	2.95748700	-0.78019200
O	0.22308100	-1.71292200	0.01556400
C	-1.39450900	0.59362400	-0.60834100
C	-2.16920700	1.76858300	-1.12488300

H	-2.28023600	2.53365400	-0.35408600
H	-1.62890800	2.22719500	-1.94929000
H	-3.15493700	1.44387000	-1.45059400
O	6.36995300	-0.19752100	-0.32147600
C	7.09721200	-0.29924900	0.82685500
C	8.56093000	-0.20560700	0.53262800
H	8.76703900	0.71934400	-0.00664500
H	9.12124000	-0.23509500	1.46275600
H	8.85125500	-1.03952000	-0.10816400
O	6.58913200	-0.44060900	1.90360100
N	-2.05514100	-0.50335200	-0.29524100
H	-1.45377200	-1.30037800	-0.05410200
C	-3.46863700	-0.69505400	-0.23834100
C	-4.03206600	-1.80831800	-0.84284100
C	-4.28328100	0.18786200	0.49217900
C	-5.40227300	-2.03184400	-0.75174600
H	-3.40005600	-2.49631800	-1.39530600
C	-5.64390000	-0.02924800	0.57879500
C	-6.28739100	-1.16596600	-0.05203500
H	-5.84017700	-2.90058800	-1.23193600
H	-3.82146300	1.02837800	0.99443000
O	-6.49388400	0.78867800	1.26916600
O	-7.54328500	-1.35824900	0.03547500
C	-5.93265700	1.91421600	1.91378800
H	-5.20091500	1.61381000	2.67075700
H	-5.45067900	2.58432700	1.19402300
H	-6.75741300	2.43458400	2.39622300

A-3O$^{\cdot+}$

G= -1353.080007 au

1 2	x	y	z
C	1.02300700	2.21828000	-0.10461000
C	2.99058400	0.84344800	-0.21333700
C	2.27191400	-0.34527300	-0.19510200
C	0.80728100	-0.31199700	-0.16359600
C	0.17979900	1.01557100	-0.18270000
H	4.93022700	1.78058600	-0.28091000
C	4.37983300	0.84925400	-0.26680100
C	2.96687900	-1.56160500	-0.23146800
C	4.34412400	-1.58420900	-0.28654000
C	5.03222700	-0.36796500	-0.30222600
H	2.39555400	-2.48121700	-0.22258400
H	4.89179900	-2.51646800	-0.32099900
O	2.38137600	2.06136500	-0.16756800

O	0.63210600	3.34695700	0.03266000
O	0.16362700	-1.36506100	-0.16028200
C	-1.20380900	1.17160400	-0.27994100
C	-1.88313600	2.47900200	-0.53992900
H	-2.00135100	3.05031100	0.38393200
H	-1.26738400	3.07968200	-1.20330300
H	-2.85502300	2.31609800	-0.99936000
O	6.40784400	-0.34196100	-0.42828300
C	7.17057000	-0.92203400	0.54758200
C	8.62041800	-0.84159100	0.19215000
H	8.90929500	0.20570300	0.09385900
H	9.21072600	-1.32358700	0.96613300
H	8.78515200	-1.32644500	-0.77075400
O	6.69537500	-1.40251800	1.53609400
N	-1.95761600	0.03824700	-0.24406100
H	-1.37290700	-0.82172100	-0.28181800
C	-3.30678300	-0.14074300	-0.10386900
C	-3.79493100	-1.42318100	-0.53007500
C	-4.17897600	0.79209800	0.46859400
C	-5.11796800	-1.74269000	-0.44297000
H	-3.09084400	-2.13207200	-0.94867400
C	-5.51482700	0.47805300	0.57126000
C	-6.00914800	-0.79657100	0.09908600
H	-5.47479100	-2.70378600	-0.78528000
H	-3.84089800	1.73095300	0.87903800
O	-6.35773900	1.33440700	1.12299100
H	-7.25648600	0.97061700	1.13758100
O	-7.29669900	-0.94979800	0.25363200
C	-7.93745700	-2.17607100	-0.15509100
H	-8.98450400	-2.04664400	0.09508200
H	-7.81163900	-2.30739100	-1.22930600
H	-7.50833800	-3.00975800	0.39935600

A-4O$^{\cdot+}$

G= -1353.080923 au

1 2	x	y	z
C	0.79693100	2.04333800	-0.32166900
C	2.86065700	0.81409600	-0.29205000
C	2.23156800	-0.42217100	-0.21885100
C	0.76814100	-0.49717300	-0.22718800
C	0.04560400	0.77749100	-0.34400000
H	4.72526700	1.89094100	-0.36972700
C	4.24643800	0.92250700	-0.31059400
C	3.01610100	-1.58251800	-0.16258700

C	4.39229000	-1.50324200	-0.18150300
C	4.98808800	-0.24143200	-0.25507100
H	2.51500000	-2.54092300	-0.11035500
H	5.00906100	-2.39156800	-0.14625500
O	2.16364700	1.98357300	-0.33886600
O	0.32070500	3.14570600	-0.26653800
O	0.20292500	-1.59251800	-0.17585100
C	-1.34109400	0.82505500	-0.48084700
C	-2.10597400	2.05967500	-0.84276200
H	-2.29295800	2.68163400	0.03587000
H	-1.51553900	2.65935700	-1.52964000
H	-3.04910500	1.79463100	-1.31500200
O	6.36187600	-0.12196300	-0.34460900
C	7.12359100	-0.53140600	0.71456400
C	8.57702100	-0.36844800	0.40526200
H	8.77949500	0.67354900	0.15465200
H	9.16761300	-0.67217300	1.26485600
H	8.83085900	-0.97737300	-0.46354500
O	6.64331500	-0.94683100	1.72990500
N	-2.01425700	-0.35704000	-0.38723600
H	-1.37152300	-1.17502000	-0.36617700
C	-3.34702200	-0.61882700	-0.24161700
C	-3.75128300	-1.95029800	-0.59236300
C	-4.27691500	0.29730600	0.28115000
C	-5.05189000	-2.33918600	-0.48178500
H	-3.00406700	-2.63531200	-0.97409400
C	-5.58998500	-0.09385000	0.40342300
C	-5.99981100	-1.42077500	0.00669200
H	-5.38060300	-3.33148700	-0.75991100
H	-3.96548000	1.26682600	0.63390100
O	-6.58818900	0.62608700	0.88536600
O	-7.25325500	-1.77382200	0.12067600
H	-7.78452900	-1.04128900	0.47928300
C	-6.33328100	1.97203700	1.31426700
H	-5.61954500	1.96636600	2.13880000
H	-5.95612900	2.55890300	0.47556600
H	-7.29126400	2.35729000	1.64695700

Cartesian coordinates (A-3OH-adducts)

C-3

G= -1429.047429 au

0 2	x	y	z
C	1.05574200	2.64129400	-0.58394400

C	2.58228200	0.82123600	-0.34758000
C	2.04825900	0.39982300	0.86788300
C	0.91947900	1.12568600	1.46804000
C	0.16593900	2.04471500	0.50652000
H	4.05281900	0.50316500	-1.88590900
C	3.64617600	0.15999700	-0.94365600
C	2.61224000	-0.71659800	1.49795300
C	3.66717700	-1.39716900	0.92389500
C	4.17079600	-0.94437000	-0.29608600
H	2.19170200	-1.03913600	2.44278400
H	4.09996900	-2.26781700	1.39849300
O	2.12268900	1.93400300	-1.01802100
O	0.80503600	3.69123400	-1.09364500
O	0.49414200	0.91458800	2.58676000
C	-0.84227300	1.11846500	-0.14278200
C	-0.56417100	0.45143600	-1.45474600
H	-0.42232100	1.18375100	-2.25424300
H	0.34361900	-0.16707400	-1.40594400
H	-1.38135400	-0.20772700	-1.73934900
O	5.17906500	-1.63597900	-0.93951000
C	6.39314200	-1.75466400	-0.32130200
C	7.31398400	-2.60197500	-1.13932500
H	7.43619600	-2.15541400	-2.12686200
H	8.27499300	-2.68145000	-0.63928000
H	6.87186100	-3.59047200	-1.27098500
O	6.63723100	-1.22667700	0.72537300
N	-1.76118600	0.61771500	0.73818800
H	-1.63237800	0.87005000	1.71364400
C	-2.98937300	0.00933200	0.41772500
C	-3.54551400	-0.89987200	1.31311100
C	-3.67559900	0.34080600	-0.75745400
C	-4.78070200	-1.48735700	1.04168600
H	-3.01284200	-1.15420200	2.22141100
C	-4.88908800	-0.26125000	-1.03360300
C	-5.45139500	-1.18170200	-0.13334000
H	-5.20109400	-2.18950400	1.74970200
H	-3.28833400	1.08364900	-1.44443400
O	-5.55179400	0.05906200	-2.17390200
O	-6.65244700	-1.68623000	-0.53102200
O	-0.43670000	3.06710200	1.25012200
H	-0.99371500	3.57696100	0.64739000
H	-6.38750400	-0.42667800	-2.18504100
C	-7.31997500	-2.57050300	0.35835500
H	-7.52189300	-2.07621400	1.31201700

H	-8.25674900	-2.83490900	-0.12506400
H	-6.72520100	-3.47177600	0.52700600

C-5

G= -1429.060700 au

0 2	x	y	z
C	0.94007000	2.03607800	-0.41879600
C	2.90911400	0.65144200	-0.39158500
C	2.19194200	-0.50053200	-0.25830700
C	0.74412200	-0.45314900	-0.22284500
C	0.11768400	0.84392300	-0.35149400
H	4.85353500	1.59452300	-0.57585300
C	4.31902700	0.66181100	-0.45870000
C	2.86622300	-1.84403100	-0.20072100
C	4.35709900	-1.75590300	-0.20484000
C	4.99709100	-0.57192300	-0.34801200
H	4.91951800	-2.67642400	-0.10801800
O	2.31369600	1.86782000	-0.46310800
O	0.57476900	3.18794800	-0.42897800
O	0.11033500	-1.52760900	-0.09623900
C	-1.30284800	0.97583300	-0.37929700
C	-1.99136700	2.27577200	-0.66237300
H	-1.94435900	2.92928500	0.21094600
H	-1.47524400	2.79178700	-1.46838800
H	-3.03086400	2.10527900	-0.93039900
O	6.38387400	-0.56984800	-0.44459300
C	7.08717000	-0.00871900	0.57928200
C	8.55592800	-0.06174500	0.30234300
H	8.76605000	0.44666600	-0.63927200
H	9.09617400	0.41150800	1.11736000
H	8.86573900	-1.10240800	0.19792300
O	6.55512500	0.44702300	1.55237800
N	-2.04375600	-0.10514900	-0.19730600
H	-1.50611600	-0.97807500	-0.09898700
C	-3.46503700	-0.18400600	-0.10592700
C	-4.13739600	-1.09275100	-0.90716700
C	-4.15408300	0.59089400	0.82980900
C	-5.52270000	-1.22545500	-0.79458900
H	-3.58917600	-1.69231300	-1.62288300
C	-5.52593600	0.46753100	0.93281900
C	-6.21851000	-0.44600600	0.11682500
H	-6.04229200	-1.93492800	-1.42443400
H	-3.63171000	1.27989500	1.48258900
O	-6.20535500	1.21894100	1.83272100

O	-7.55834700	-0.46709200	0.32935200
H	2.55796700	-2.40823900	-1.09706100
O	2.47043400	-2.58790600	0.94974800
H	1.50876400	-2.67425300	0.88019900
H	-7.14309800	0.99127900	1.77254800
C	-8.33699000	-1.37665500	-0.43907700
H	-8.02314400	-2.40570500	-0.24883700
H	-9.36529300	-1.23936200	-0.11615700
H	-8.25001100	-1.15113700	-1.50447000

C-6

G= -1429.051863 au

0 2	x	y	z
C	0.69385900	2.21158200	-0.08909500
C	2.75752600	0.97459600	-0.09693100
C	2.10945900	-0.26597200	0.00480500
C	0.62953100	-0.30253400	-0.02503300
C	-0.05492400	0.96779000	-0.11621500
H	4.61109100	2.00883000	-0.52873500
C	4.15943800	1.05981500	-0.26772000
C	2.84956100	-1.41650000	0.07303700
C	4.33515700	-1.39311900	0.25916500
C	4.90282600	-0.06862600	-0.18886700
H	2.34825500	-2.37504600	0.11151700
O	2.07605500	2.14107900	-0.12259600
O	0.25320500	3.33476300	-0.03397200
O	0.05502500	-1.39988800	0.01953100
C	-1.47967800	1.02456800	-0.20194300
C	-2.23052100	2.29797000	-0.44654300
H	-2.24447900	2.90963000	0.45740400
H	-1.71981400	2.87924800	-1.21072600
H	-3.25029000	2.08528700	-0.75657500
O	6.20447000	0.02756500	-0.65040900
C	7.24309500	-0.63791900	-0.09494300
C	8.49757100	-0.40224800	-0.87238200
H	8.67793900	0.66949800	-0.95908200
H	9.32996400	-0.89076300	-0.37421000
H	8.37316100	-0.80397600	-1.87926800
O	7.14277500	-1.31722900	0.89418000
N	-2.16763200	-0.10141900	-0.11350300
H	-1.58931200	-0.94889200	-0.03043100
C	-3.58687600	-0.25883900	-0.11670600
C	-4.16439800	-1.10130300	-1.05241300
C	-4.36533700	0.37312700	0.85528100

C	-5.54427400	-1.31434000	-1.03620700
H	-3.54654000	-1.58827700	-1.79629400
C	-5.73183600	0.16903300	0.86558100
C	-6.32841300	-0.68067700	-0.08464200
H	-5.99004900	-1.97183900	-1.77029100
H	-3.91661900	1.01335300	1.60535800
O	-6.49831900	0.77842000	1.80230500
O	-7.67394100	-0.79569600	0.04868300
H	4.80574300	-2.20202300	-0.31165900
O	4.54416300	-1.62353100	1.66127200
H	5.50335200	-1.65977400	1.78523900
H	-7.41683400	0.50916300	1.66627700
C	-8.35798400	-1.65277100	-0.85766800
H	-8.23720600	-1.29932000	-1.88427000
H	-7.98857000	-2.67711100	-0.76955700
H	-9.40652900	-1.61372800	-0.57549300

C-7

G= -1429.061075 au

0 2	x	y	z
C	0.95422400	2.00254500	-0.60615400
C	2.96200800	0.66004700	-0.64137800
C	2.23726400	-0.52880200	-0.41916800
C	0.77920800	-0.48874400	-0.31021600
C	0.14533400	0.80148900	-0.46807600
H	4.85324000	1.59253600	-0.95095900
C	4.31243100	0.67094700	-0.77371700
C	2.93943500	-1.76770700	-0.32457300
C	4.28071000	-1.81989400	-0.43086300
C	5.10418100	-0.59455300	-0.70644300
H	2.35579700	-2.66384500	-0.15664600
H	4.82657200	-2.75453100	-0.35101900
O	2.31864700	1.86350100	-0.71652800
O	0.56014000	3.14500100	-0.62510500
O	0.16089900	-1.55093900	-0.10942500
C	-1.27630500	0.92213000	-0.45528100
C	-1.99040100	2.20009600	-0.77604000
H	-1.93535100	2.88939100	0.06857500
H	-1.50175100	2.69048800	-1.61424800
H	-3.03275000	2.00254300	-1.01330000
O	6.16656600	-0.48227600	0.28804500
C	5.85069600	-0.29471500	1.58480100
C	7.08706500	-0.23767400	2.43269100
H	7.76233200	0.52744000	2.04888400

H	6.81265600	-0.01977200	3.46131700
H	7.60280300	-1.19766300	2.37829600
O	4.71942700	-0.19331000	1.98282000
N	-2.00131800	-0.15610200	-0.20228100
H	-1.45040000	-1.01803600	-0.07924500
C	-3.41897500	-0.24638000	-0.07446200
C	-4.09904900	-1.18855300	-0.82944600
C	-4.09508300	0.55064500	0.85189700
C	-5.47909600	-1.33559600	-0.67745500
H	-3.56040600	-1.80400100	-1.53891300
C	-5.46204100	0.41242600	0.99422000
C	-6.16213300	-0.53687500	0.22685100
H	-6.00466200	-2.07190900	-1.27042600
H	-3.56593500	1.26798000	1.46755800
O	-6.12910000	1.18448500	1.88603000
O	-7.49535600	-0.57009700	0.47644800
O	5.79576500	-0.70755200	-1.91703700
H	6.27411100	-1.54672000	-1.92810100
H	-7.06451600	0.94112800	1.85908200
C	-8.28040400	-1.51602200	-0.23977400
H	-8.22861900	-1.32277800	-1.31373700
H	-7.94331600	-2.53325600	-0.02723200
H	-9.30089600	-1.38564900	0.10962000

C-8

G= -1429.057924 au

0 2	x	y	z
C	0.79269600	1.96287700	-0.43693300
C	2.77590400	0.60799200	-0.39897900
C	2.10100300	-0.56283300	-0.25464200
C	0.62801300	-0.53370100	-0.20876300
C	-0.00788000	0.75976100	-0.35059000
C	4.27052500	0.72771800	-0.43952400
C	2.81497700	-1.79058900	-0.16220300
C	4.21890800	-1.79109700	-0.22316000
C	4.90244000	-0.62275300	-0.35850600
H	2.25841900	-2.70969000	-0.04819100
H	4.76974500	-2.72225800	-0.16635200
O	2.17274300	1.80707600	-0.49137900
O	0.41838500	3.11087600	-0.45860900
O	0.00824700	-1.59919200	-0.06612800
C	-1.43143100	0.87656400	-0.37452500
C	-2.13415700	2.16498700	-0.67516500
H	-2.08751700	2.83331800	0.18680500

H	-1.62847900	2.67235400	-1.49329000
H	-3.17364900	1.98020800	-0.93345400
O	6.27692700	-0.58397700	-0.48025100
C	7.05266500	-0.84308200	0.61425000
C	8.50007800	-0.67211300	0.27596900
H	8.68271600	0.37150300	0.01439500
H	9.10932600	-0.95480600	1.12986000
H	8.75014700	-1.28346000	-0.59145800
O	6.59728400	-1.15650400	1.67660800
N	-2.16149600	-0.20696000	-0.17281900
H	-1.61537000	-1.07271400	-0.06079200
C	-3.58286400	-0.29528300	-0.07973900
C	-4.25188000	-1.20519900	-0.88194700
C	-4.27337300	0.47424000	0.85915900
C	-5.63639100	-1.34496200	-0.76672500
H	-3.70208700	-1.80022900	-1.60021400
C	-5.64441200	0.34407000	0.96473400
C	-6.33402100	-0.57120700	0.14812300
H	-6.15388600	-2.05537300	-1.39723300
H	-3.75261200	1.16492500	1.51151700
O	-6.32578800	1.09043200	1.86732300
O	-7.67327700	-0.59958300	0.36351600
H	4.56592900	1.21633500	-1.38017000
O	4.75323000	1.51303400	0.65196800
H	4.65138900	2.44405300	0.42683100
H	-7.26254100	0.85842400	1.80827900
C	-8.44917000	-1.51065200	-0.40594100
H	-8.36665900	-1.28069900	-1.47074300
H	-8.12900200	-2.53861000	-0.22047400
H	-9.47720000	-1.38030300	-0.07933300

C-9

G= -1429.057173 au

0 2	x	y	z
C	0.90809100	2.09887500	0.02954400
C	2.83017100	0.69796300	0.45164300
C	2.18571500	-0.39614600	-0.34013000
C	0.71535700	-0.37049500	-0.39369200
C	0.08955800	0.89152100	-0.06637500
H	4.82321900	1.65710800	0.66809500
C	4.30984000	0.80254900	0.24236000
C	2.92589600	-1.36755100	-0.95652800
C	4.32467400	-1.30163200	-0.97800800
C	4.97805300	-0.17995500	-0.38801700

H	2.41077600	-2.17020300	-1.47051700
H	4.91385400	-2.05708000	-1.48062500
O	2.27047300	1.96190100	0.09286200
O	0.49636200	3.23602000	-0.00157700
O	0.09648600	-1.39840700	-0.71691000
C	-1.31960000	0.99702100	0.07364100
C	-1.98816100	2.22651600	0.61330200
H	-1.35745100	2.68780600	1.36835100
H	-2.12974900	2.96282000	-0.17990500
H	-2.95706400	1.97111700	1.03745800
O	6.35227900	-0.05635800	-0.55234300
C	7.14450100	-0.94390400	0.11194700
C	8.58656100	-0.70645100	-0.20485800
H	8.84094000	0.33233600	0.00781400
H	9.20386700	-1.37746200	0.38563500
H	8.75204500	-0.88144700	-1.26905000
O	6.69619300	-1.78373500	0.84157700
N	-2.07319200	-0.05597900	-0.21485600
H	-1.54638200	-0.88778400	-0.50866500
C	-3.49369200	-0.15733200	-0.15405100
C	-4.30807500	0.72733200	-0.84354500
C	-4.04408300	-1.22077000	0.56496900
C	-5.69442500	0.57155300	-0.80083300
H	-3.87164000	1.52999900	-1.42419000
C	-5.41612900	-1.38172500	0.59721200
C	-6.25050200	-0.47795400	-0.08482100
H	-6.32404000	1.26648200	-1.33997300
H	-3.41162600	-1.91664400	1.10303300
O	-5.95960900	-2.40972400	1.29340200
O	-7.57456600	-0.75177900	0.03499400
O	2.53786000	0.44225400	1.80870300
H	3.00222400	1.09103100	2.35420700
H	-6.92123800	-2.36258500	1.20426200
C	-8.49052700	0.10591900	-0.63499100
H	-8.40550600	1.12739600	-0.25700100
H	-8.31175100	0.09096000	-1.71260400
H	-9.48130500	-0.28538800	-0.42060700

C-10

G= -1429.043820 au

0 2	x	y	z
C	0.86475300	2.17751200	-0.39571300
C	2.89517300	0.94266700	-0.14526300
C	2.29791100	0.12700300	0.94669900

C	0.80099100	-0.09654500	0.64964200
C	0.12569300	0.97245300	-0.03669600
H	4.48541100	1.32206200	-1.50346100
C	4.08315100	0.66111000	-0.74596500
C	3.03380600	-1.15939100	1.15116000
C	4.22195400	-1.42058000	0.55573400
C	4.77037100	-0.50357100	-0.37662700
H	2.58527900	-1.86622900	1.83818200
H	4.75559700	-2.33949200	0.76565200
O	2.23893700	2.09020700	-0.47547800
O	0.40119000	3.26105400	-0.65136800
O	0.26696500	-1.12553200	1.06720600
C	-1.26951900	0.91271400	-0.30252700
C	-1.97927000	1.94758800	-1.12311300
H	-2.21170300	2.82209400	-0.51256800
H	-1.33384100	2.28402600	-1.93034200
H	-2.90240300	1.54024600	-1.52896700
O	5.94229100	-0.78832600	-1.04500000
C	7.08076000	-1.00482400	-0.32157200
C	8.21026400	-1.38697000	-1.22383000
H	8.33665600	-0.62605200	-1.99453200
H	9.12115300	-1.49209300	-0.64142800
H	7.96962000	-2.32882900	-1.71932600
O	7.11912600	-0.88808900	0.87048200
N	-1.96734000	-0.12434800	0.13640800
H	-1.42296600	-0.84362800	0.62147800
C	-3.37521300	-0.33111600	0.01146900
C	-3.83442600	-1.49858700	-0.57549900
C	-4.26790200	0.60824000	0.53245800
C	-5.20758500	-1.73665700	-0.66344300
H	-3.12970900	-2.21969700	-0.97041800
C	-5.62644800	0.37814300	0.43450600
C	-6.10289900	-0.80154500	-0.16691300
H	-5.56207800	-2.64904300	-1.12383200
H	-3.91438200	1.50947500	1.01870400
O	-6.50197900	1.28494600	0.93131100
O	-7.45597600	-0.89919700	-0.18894500
O	2.34352900	0.95197600	2.13659800
H	1.95437000	0.44906700	2.86377000
H	-7.39879800	0.95527500	0.78358700
C	-8.02726700	-2.06784200	-0.76500100
H	-7.74579100	-2.15387100	-1.81705600
H	-7.70943600	-2.95918900	-0.21901900
H	-9.10404600	-1.94880400	-0.68078900

C-1’

G= -1429.054884 au

0 2	x	y	z
C	0.79381500	1.66511700	0.49024600
C	2.95270700	0.75568600	-0.03393500
C	2.48339100	-0.54942200	-0.14064100
C	1.06906600	-0.82747600	0.11224300
C	0.24955100	0.30206900	0.57205100
H	4.64691900	2.08046900	-0.16082300
C	4.29137500	1.06202500	-0.24311600
C	3.38113400	-1.57088700	-0.47949700
C	4.71362500	-1.29259900	-0.69733200
C	5.15000000	0.02947900	-0.57025500
H	3.00222600	-2.58176300	-0.56499500
H	5.41505200	-2.07226300	-0.96195800
O	2.13256000	1.80779400	0.25867300
O	0.15233900	2.67375000	0.60812900
O	0.57481500	-1.94215300	-0.04151100
C	-1.20677700	0.03062900	0.95242800
C	-1.87260900	1.12063200	1.78036300
H	-1.24219400	1.36268000	2.63604100
H	-2.05536800	2.02022200	1.20227500
H	-2.81861800	0.71784500	2.14306900
O	6.45895700	0.36906800	-0.84562900
C	7.46225300	-0.20428200	-0.11231700
C	8.79528900	0.26101600	-0.60317100
H	8.86412200	1.34296400	-0.48064700
H	9.58211200	-0.23138000	-0.03892500
H	8.89039400	0.03731900	-1.66613200
O	7.24826300	-0.95537800	0.79492700
N	-1.85106900	-0.13685000	-0.34872300
H	-1.49287500	-0.97809700	-0.79225400
C	-3.28557100	-0.10217800	-0.38426100
C	-3.92769600	1.06901700	-0.75806200
C	-4.04034500	-1.23230600	-0.04932500
C	-5.32257700	1.13353700	-0.79051400
H	-3.33654800	1.93536100	-1.02866700
C	-5.42171200	-1.17808700	-0.09439600
C	-6.07075800	0.01293500	-0.46128900
H	-5.80946600	2.05473500	-1.08201000
H	-3.55530400	-2.15485100	0.24775400
O	-6.15485600	-2.27741800	0.22199300
O	-7.42911600	-0.07278600	-0.45932400

O	-1.27019500	-1.12248300	1.77803900
H	-0.85385800	-1.85468400	1.30357800
H	-7.09060000	-2.05092500	0.13667900
C	-8.16203700	1.08143600	-0.84706900
H	-9.21189000	0.80678000	-0.78809600
H	-7.95893000	1.91299800	-0.16778800
H	-7.91256600	1.36934300	-1.87132400

C-1’’

G= -1429.059075 au

0 2	x	y	z
C	0.78936900	1.78915300	-0.08088400
C	2.98235800	0.80271100	-0.24606000
C	2.47977700	-0.48892700	-0.30914500
C	1.02199000	-0.70746300	-0.25689500
C	0.18782200	0.46976200	-0.14918300
H	4.73241000	2.06528200	-0.24102900
C	4.35220400	1.05332100	-0.29139600
C	3.37298600	-1.56020100	-0.42532300
C	4.73522700	-1.34044500	-0.47748000
C	5.20234000	-0.02736500	-0.40718300
H	2.96809200	-2.56303500	-0.47717900
H	5.43929600	-2.15718100	-0.57241000
O	2.16766700	1.88295900	-0.13589300
O	0.22518200	2.85362000	0.02228100
O	0.58841100	-1.86899600	-0.30949200
C	-1.24053500	0.35284300	-0.10567000
C	-2.13885900	1.54875900	-0.01140400
H	-1.90269000	2.10763400	0.89423800
H	-1.93734700	2.21729000	-0.84823600
H	-3.18588300	1.27146100	-0.00724200
O	6.56262100	0.22429600	-0.51571000
C	7.35219300	-0.06515600	0.55734500
C	8.78595900	0.23110300	0.25066700
H	8.89191000	1.28969200	0.00977400
H	9.40099100	-0.02281700	1.10934600
H	9.09594000	-0.34397700	-0.62261900
O	6.91060800	-0.49067600	1.58754300
N	-1.77355900	-0.84921800	-0.15486000
H	-1.10323800	-1.62667500	-0.22337100
C	-3.19816200	-1.25774700	-0.12982200
C	-3.93970400	-0.74759000	-1.33087800
C	-3.85499900	-0.91597200	1.16923100
C	-5.15407000	-0.13308600	-1.23838200

H	-3.47578900	-0.93603800	-2.29143900
C	-5.05868600	-0.30682800	1.22116900
C	-5.73806500	0.10520400	0.01912000
H	-5.66373300	0.17973500	-2.14054900
H	-3.34653700	-1.21335200	2.07847200
O	-5.65468700	-0.05220500	2.41094500
O	-6.91849500	0.70164600	0.24152900
O	-3.02453900	-2.67917500	-0.24323700
H	-3.89627200	-3.08323200	-0.16460000
H	-6.49267200	0.40104200	2.24830100
C	-7.66766200	1.17844800	-0.87646400
H	-7.93384000	0.34905200	-1.53394500
H	-8.56579100	1.62665300	-0.46200700
H	-7.09296800	1.92808900	-1.42339800

C-2’’

G= -1429.054966 au

0 2	x	y	z
C	1.14566500	2.07356300	-0.31287100
C	3.17931600	0.78537800	-0.33722600
C	2.50525600	-0.42697300	-0.28978800
C	1.03138400	-0.43964600	-0.26306400
C	0.37018000	0.84581100	-0.32842800
H	5.08535800	1.79308000	-0.41366100
C	4.57041400	0.84204700	-0.37608000
C	3.24547000	-1.61518700	-0.28314400
C	4.62513900	-1.58822700	-0.32550600
C	5.26683700	-0.34948200	-0.37033900
H	2.70761000	-2.55436000	-0.24950300
H	5.21007700	-2.49891800	-0.32643400
O	2.52216300	1.97442300	-0.34004900
O	0.72405800	3.20531200	-0.26623900
O	0.43521700	-1.52577600	-0.20400100
C	-1.05135300	0.93105100	-0.38683300
C	-1.78837100	2.21668700	-0.60615600
H	-1.76617700	2.82684000	0.29897000
H	-1.29481500	2.79470700	-1.38406800
H	-2.81879400	2.01587400	-0.89388900
O	6.64760300	-0.28567600	-0.47809900
C	7.39740700	-0.68123600	0.59030500
C	8.85561400	-0.57529600	0.27464000
H	9.09285700	0.45463200	0.00481600
H	9.43823800	-0.88229500	1.13853900
H	9.08706200	-1.20775600	-0.58338400

O	6.90971100	-1.04735000	1.62207300
N	-1.76884000	-0.17820700	-0.28935600
H	-1.21410200	-1.04081700	-0.21058900
C	-3.19237600	-0.26998200	-0.24905700
C	-3.86053700	-0.96366500	-1.19819800
C	-3.85522600	0.25917000	0.99395500
C	-5.27446500	-1.13810100	-1.14795400
H	-3.31039900	-1.38510800	-2.03186200
C	-5.33734500	0.13883500	0.90032200
C	-5.98975000	-0.56568100	-0.10125200
H	-5.76997500	-1.69104400	-1.93230400
O	-6.01784200	0.72836000	1.89534100
O	-7.34333500	-0.60751700	0.05271100
H	-3.60580600	1.31569900	1.16179800
O	-3.42351100	-0.48014500	2.15113500
H	-2.62303400	-0.07106900	2.49638200
H	-6.96413000	0.57291400	1.75692700
C	-8.10480500	-1.25402600	-0.95937200
H	-9.14498700	-1.15821000	-0.65960400
H	-7.95244100	-0.76869200	-1.92630000
H	-7.83790000	-2.31132400	-1.02704400

C-3’’

G= -1429.064419 au

0 2	x	y	z
C	1.24752300	2.03400400	-0.34971400
C	3.27008800	0.72737700	-0.34555000
C	2.58766600	-0.47159600	-0.19267200
C	1.11479700	-0.46743900	-0.12985600
C	0.46174300	0.81500600	-0.27286100
H	5.18001200	1.70826000	-0.55171500
C	4.65993300	0.76705800	-0.43017800
C	3.31729500	-1.66385100	-0.12054800
C	4.69559100	-1.65349700	-0.20379400
C	5.34551300	-0.42845100	-0.35938300
H	2.77370500	-2.59295500	-0.00368700
H	5.27413600	-2.56723500	-0.15642400
O	2.62224800	1.91944800	-0.41090800
O	0.83822100	3.17103200	-0.35544500
O	0.51306000	-1.54235600	0.02012300
C	-0.96227500	0.90806000	-0.31296800
C	-1.68586000	2.18214900	-0.62602800
H	-1.66192400	2.85025700	0.23689500
H	-1.17950100	2.69840900	-1.43797900

H	-2.71917400	1.97775600	-0.89339700
O	6.72441500	-0.39806400	-0.50924500
C	7.49108600	-0.58647200	0.60276900
C	8.94454500	-0.55380100	0.25112500
H	9.19233100	0.42681200	-0.15794500
H	9.53782900	-0.75062400	1.13952400
H	9.14977000	-1.29935200	-0.51766400
O	7.01810200	-0.74304200	1.69314700
N	-1.67471400	-0.18907200	-0.11657800
H	-1.11497000	-1.04534800	0.00812800
C	-3.09848500	-0.30695500	-0.07215800
C	-3.70910400	-1.17127500	-1.01798500
C	-3.81583400	0.35937400	0.86412100
C	-5.10728300	-1.32136600	-1.03240700
H	-3.09416400	-1.68499900	-1.74466000
C	-5.29888600	0.19413700	0.98695100
C	-5.88104400	-0.66253200	-0.11634600
H	-5.56115900	-1.94598500	-1.78982700
H	-3.34181800	1.01373200	1.58782500
O	-5.87032000	1.48240300	0.97831800
O	-7.21538600	-0.66467300	-0.05379100
O	-5.63923500	-0.46447200	2.19746600
H	-5.28829000	0.05889300	2.92918100
H	-6.83066900	1.37393400	1.00450800
C	-7.93453600	-1.40511900	-1.03785700
H	-8.98695300	-1.25111100	-0.81828600
H	-7.70120400	-1.03144000	-2.03706300
H	-7.68863300	-2.46658400	-0.96667000

C-4’’

G= -1429.063750 au

0 2	x	y	z
C	1.31760700	2.14574300	-0.19325300
C	3.30597300	0.79290300	-0.28513200
C	2.59132600	-0.39698100	-0.25002900
C	1.12030500	-0.36065400	-0.20829500
C	0.49895600	0.94540100	-0.23638900
H	5.24424000	1.73637600	-0.36955000
C	4.69749400	0.80291500	-0.34192400
C	3.28992700	-1.60994200	-0.27175400
C	4.66902200	-1.62856700	-0.32995700
C	5.35205300	-0.41189200	-0.36368900
H	2.72095300	-2.53081300	-0.24770900
H	5.22321000	-2.55805200	-0.35261600

O	2.68831700	2.00268900	-0.25604200
O	0.93345900	3.28682000	-0.09065800
O	0.48711200	-1.42908400	-0.17339800
C	-0.91914100	1.08254000	-0.27692400
C	-1.60511100	2.38753900	-0.54119800
H	-1.65522100	2.99853900	0.36247600
H	-1.03144700	2.95308200	-1.27058400
H	-2.61032200	2.21326600	-0.91831500
O	6.73352600	-0.39990200	-0.48476900
C	7.47445600	-0.77249000	0.59798400
C	8.93358500	-0.74393900	0.27046100
H	9.21019900	0.25445900	-0.07001900
H	9.50930600	-1.01656400	1.15036000
H	9.13286400	-1.44270600	-0.54316100
O	6.97847100	-1.06841200	1.64811200
N	-1.65837800	-0.01919700	-0.16086500
H	-1.09174000	-0.88568500	-0.15209000
C	-3.04483500	-0.16782700	-0.05142500
C	-3.61863900	-1.32583000	-0.65835400
C	-3.85970500	0.73238500	0.67107400
C	-4.93786500	-1.59652500	-0.56331200
H	-2.96887800	-1.98308400	-1.22597500
C	-5.19449000	0.49885300	0.77199900
C	-5.86633700	-0.75224600	0.25586500
H	-5.35907600	-2.48359500	-1.01984200
H	-3.44799800	1.61121500	1.14855200
O	-5.99824000	1.35860100	1.42939300
O	-7.00083700	-0.26790200	-0.43583300
O	-6.28409900	-1.57118200	1.33721200
H	-6.87498400	-1.06388200	1.90939000
C	-7.81749100	-1.24857600	-1.07215400
H	-8.02403500	-2.08053800	-0.39669400
H	-8.74843300	-0.74868600	-1.33011700
H	-7.34074200	-1.61513200	-1.98395100
H	-6.90347500	1.24629300	1.10218800

C-5’’

G= -1429.052688 au

0 2	x	y	z
C	1.27777700	2.08086300	-0.40865800
C	3.27787800	0.74116000	-0.36333600
C	2.57758500	-0.43592800	-0.13734900
C	1.10621400	-0.40327600	-0.05400800
C	0.47307000	0.88144300	-0.25685800

H	5.20142700	1.67527600	-0.64578800
C	4.66682600	0.75126500	-0.46843300
C	3.28779600	-1.63529500	-0.01194100
C	4.66457400	-1.65428900	-0.11474100
C	5.33326400	-0.45069500	-0.34260400
H	2.73040700	-2.54714200	0.16204000
H	5.22688300	-2.57503300	-0.02624200
O	2.64921700	1.93976000	-0.48279900
O	0.88583900	3.22244000	-0.46904600
O	0.48858100	-1.45715900	0.16232200
C	-0.94835800	0.99614100	-0.29047000
C	-1.65760500	2.26400000	-0.65790900
H	-1.61059900	2.97429100	0.16969400
H	-1.15726300	2.73270600	-1.50210500
H	-2.69718000	2.06228100	-0.90437200
O	6.71016400	-0.44985600	-0.51076600
C	7.48946000	-0.63112100	0.59383400
C	8.93773600	-0.63541800	0.22008200
H	9.20118500	0.33531300	-0.20269200
H	9.53995200	-0.83590300	1.10165300
H	9.11496600	-1.39296600	-0.54384300
O	7.03026600	-0.75485500	1.69420200
N	-1.68021600	-0.07626400	-0.03767600
H	-1.14093400	-0.93643300	0.12856200
C	-3.10761900	-0.15590600	0.01181800
C	-3.74251800	-1.02383200	-0.80662800
C	-3.80024600	0.64728200	0.96727800
C	-5.21830300	-1.26195200	-0.70943900
H	-3.18631700	-1.60962700	-1.52900900
C	-5.18708400	0.58369400	1.01719100
C	-5.89140800	-0.27787900	0.19215900
H	-5.66169700	-1.20655100	-1.71255700
H	-3.26216900	1.30715300	1.63497100
O	-5.85223400	1.38777200	1.89066100
O	-7.22965700	-0.27108700	0.34572700
O	-5.50877700	-2.57067400	-0.17129800
H	-5.28066200	-3.22763300	-0.83886200
H	-6.79882200	1.21605300	1.80185900
C	-8.04760600	-0.85396500	-0.67352900
H	-7.81107000	-1.91084900	-0.79415900
H	-9.07168300	-0.73616400	-0.33064500
H	-7.91069700	-0.31939300	-1.61555600

C-6''

G= -1429.061971 au

0 2	x	y	z
C	1.21514600	2.21761100	-0.25772200
C	3.18493600	0.83338000	-0.30459200
C	2.45096200	-0.34404400	-0.25781600
C	0.97910800	-0.28460900	-0.23047100
C	0.37857400	1.03024300	-0.28527700
H	5.13911800	1.74493100	-0.37762700
C	4.57727000	0.82068200	-0.34226400
C	3.13012600	-1.56781600	-0.25000000
C	4.50955900	-1.60935600	-0.28941700
C	5.21239500	-0.40451300	-0.33402500
H	2.54611400	-2.47899700	-0.21741700
H	5.04928300	-2.54761600	-0.28868500
O	2.58521600	2.05240000	-0.30540500
O	0.85145100	3.36790100	-0.18025300
O	0.33135100	-1.34234300	-0.18088600
C	-1.03927900	1.18929500	-0.32394100
C	-1.70230500	2.50417600	-0.59829600
H	-1.73341600	3.12453100	0.29981100
H	-1.12610500	3.05178700	-1.33891900
H	-2.71539000	2.34275700	-0.95969600
O	6.59602800	-0.41857800	-0.43328100
C	7.31181300	-0.76850800	0.67339100
C	8.77639600	-0.78048100	0.36993500
H	9.07965300	0.19932500	-0.00045100
H	9.33205400	-1.03404300	1.26819200
H	8.97380200	-1.51145000	-0.41543600
O	6.79306300	-1.01928800	1.72443700
N	-1.80132400	0.10902300	-0.18288500
H	-1.27332600	-0.77908800	-0.14862300
C	-3.19280700	-0.00870500	-0.06983400
C	-3.72733000	-1.28067400	-0.67496100
C	-3.97772100	0.86213400	0.64305000
C	-5.21385100	-1.40940100	-0.55777100
H	-3.42989700	-1.32355300	-1.72891200
C	-5.34940600	0.63843600	0.75101900
C	-5.95533700	-0.51229100	0.12711200
H	-5.66048000	-2.27402100	-1.03005700
H	-3.55397600	1.71651400	1.15566900
O	-6.11316900	1.48653000	1.46074400
O	-7.29487300	-0.54929000	0.32457700
O	-3.04863500	-2.41603300	-0.10406600
H	-3.35771800	-2.51514500	0.80475800

H	-7.02801900	1.16952500	1.43797600
C	-8.00822100	-1.65390200	-0.21852400
H	-7.90784300	-1.67325500	-1.30611100
H	-7.63920100	-2.59039000	0.20595100
H	-9.04900900	-1.50532100	0.05527200

Cartesian coordinates (A-4OH-adducts)

C-3

G= -1429.047911 au

0 2	x	y	z
C	0.75287800	2.53168200	-0.43747400
C	2.41729200	0.82388400	-0.31411400
C	1.98621100	0.35933100	0.92675100
C	0.84816900	1.00066200	1.60101000
C	-0.02822600	1.86120100	0.69303900
H	3.81493400	0.61305000	-1.93754900
C	3.48628000	0.23902000	-0.97684600
C	2.65876100	-0.71949000	1.51249700
C	3.72204000	-1.32441100	0.87122700
C	4.11987300	-0.83149700	-0.37076800
H	2.31683600	-1.07399400	2.47725300
H	4.24046400	-2.16654500	1.31081200
O	1.84189000	1.90738200	-0.94210800
O	0.39956700	3.56603800	-0.91707200
O	0.51119900	0.75620800	2.74238800
C	-0.99495100	0.86302200	0.08934300
C	-0.74475600	0.23740500	-1.24851000
H	-0.66685100	0.99177300	-2.03591800
H	0.18875700	-0.34393100	-1.25039400
H	-1.54620900	-0.44858800	-1.51526200
O	5.13950400	-1.44695700	-1.07356600
C	6.41082500	-1.35970600	-0.57908300
C	7.36325100	-2.11432300	-1.45014600
H	7.31037900	-1.72486700	-2.46739300
H	8.37121900	-2.01393900	-1.05800400
H	7.07019200	-3.16481600	-1.47763800
O	6.67322900	-0.74479600	0.41469700
N	-1.81381800	0.26876900	1.01137000
H	-1.65891000	0.52226100	1.98256000
C	-3.02069200	-0.40512500	0.73149100
C	-3.45402600	-1.41428900	1.58522100
C	-3.79938200	-0.03611600	-0.37465600

C	-4.66136800	-2.06410500	1.33716400
H	-2.84806700	-1.69580100	2.43812400
C	-4.98728000	-0.70485500	-0.62616800
C	-5.42414500	-1.72562800	0.23384600
H	-5.01238100	-2.85034400	1.99449300
H	-3.47890700	0.78482100	-1.00267200
O	-5.82887200	-0.45250800	-1.66259000
O	-6.59478700	-2.37190200	-0.01697800
H	-6.98168800	-1.99700100	-0.81919500
C	-5.45276000	0.55613500	-2.59137500
H	-5.38035100	1.52766900	-2.09670000
H	-4.49907900	0.30698800	-3.06348700
H	-6.23914800	0.58406900	-3.34085900
O	-0.66639200	2.82842300	1.47938200
H	-1.29512000	3.29594100	0.91432800

C-5

G= -1429.059843 au

0 2	x	y	z
C	0.74473400	1.84349900	-0.80668100
C	2.78289500	0.59460000	-0.51807400
C	2.12466400	-0.57407500	-0.27412600
C	0.67647400	-0.61181500	-0.31208600
C	-0.01402200	0.62192700	-0.61844000
H	4.68052200	1.62701800	-0.72383600
C	4.19174000	0.68458300	-0.51756200
C	2.86879400	-1.85600700	-0.01561400
C	4.34997100	-1.67611700	0.04225300
C	4.93000400	-0.48123600	-0.21916500
H	4.95621200	-2.54017300	0.28524800
O	2.12631400	1.75294500	-0.77307200
O	0.31965300	2.96095700	-0.98163500
O	0.09758400	-1.70082400	-0.08745300
C	-1.43804000	0.66834800	-0.69656700
C	-2.18617900	1.89156400	-1.13096200
H	-2.16919200	2.64734900	-0.34318300
H	-1.69108200	2.33006300	-1.99428800
H	-3.21596100	1.64471000	-1.37532900
O	6.31764900	-0.40216200	-0.24604700
C	6.92925700	0.28951600	0.75645300
C	8.41138400	0.30645000	0.55674800
H	8.63997400	0.81287000	-0.38216000
H	8.88511200	0.82272200	1.38684600
H	8.78128400	-0.71669800	0.48395200

O	6.31862900	0.79702600	1.65506300
N	-2.12627600	-0.42685900	-0.41620100
H	-1.54766300	-1.25318500	-0.20942800
C	-3.54468300	-0.56290100	-0.33406200
C	-4.17130700	-1.55628900	-1.06895800
C	-4.27178000	0.25800200	0.53399100
C	-5.55015200	-1.73042800	-0.95506300
H	-3.59058200	-2.18635100	-1.73109000
C	-5.64284300	0.08908200	0.63225700
C	-6.28641100	-0.91354500	-0.11649700
H	-6.06324400	-2.49813900	-1.52090800
H	-3.75633400	1.00641600	1.12126800
O	-6.47557200	0.81544400	1.41848800
O	-7.62770500	-1.07755300	-0.00977100
H	-7.96989700	-0.41345500	0.60389900
C	-5.91450900	1.88974600	2.16385600
H	-5.18647200	1.51643800	2.88774700
H	-5.44050900	2.61200600	1.49461100
H	-6.74373000	2.35949800	2.68553300
H	2.64315500	-2.54331000	-0.84840400
O	2.45213600	-2.47763800	1.19812100
H	1.50287800	-2.63475800	1.09316500

C-6

G= -1429.052340 au

0 2	x	y	z
C	0.50912000	2.08893700	-0.31965700
C	2.62819900	0.95609900	-0.18223100
C	2.03479700	-0.30947700	-0.05116300
C	0.56240200	-0.42104400	-0.15411600
C	-0.17733800	0.80950300	-0.32602400
H	4.45008600	2.05854200	-0.57873200
C	4.03118400	1.10224700	-0.29097900
C	2.82412100	-1.41722900	0.11148400
C	4.29464300	-1.30973400	0.37107700
C	4.82149600	0.01653400	-0.11949100
H	2.36814600	-2.39694900	0.17272600
O	1.89380000	2.08480900	-0.29569500
O	0.01491700	3.19080100	-0.33056900
O	0.03952000	-1.54323900	-0.09597200
C	-1.59826900	0.79189800	-0.47154800
C	-2.40021100	2.02006400	-0.77833700
H	-2.46270300	2.66041900	0.10335600
H	-1.89719200	2.59836300	-1.55003300

H	-3.40128400	1.75117100	-1.10452500
O	6.13928600	0.15396000	-0.52123500
C	7.17699800	-0.43356100	0.11765000
C	8.45930100	-0.16648300	-0.60222800
H	8.62187000	0.91041700	-0.66201300
H	9.27872400	-0.64451500	-0.07334000
H	8.38795000	-0.55172100	-1.62015400
O	7.05590900	-1.07168400	1.13148900
N	-2.23507000	-0.36349200	-0.37912800
H	-1.62268800	-1.17977200	-0.24461700
C	-3.64631200	-0.58169600	-0.43317300
C	-4.16158500	-1.42638800	-1.40245100
C	-4.47568700	0.00685700	0.52624600
C	-5.53187300	-1.68388300	-1.43259600
H	-3.50126300	-1.87570500	-2.13363500
C	-5.83721000	-0.24563700	0.48529600
C	-6.36862200	-1.09721600	-0.50117000
H	-5.95905300	-2.33887700	-2.18179700
H	-4.04547600	0.64397300	1.28794700
O	-6.76174700	0.25520300	1.34177100
O	-7.70117200	-1.34335500	-0.53087700
H	-8.11969800	-0.84952100	0.18723300
C	-6.31149900	1.14267600	2.35865400
H	-5.60567200	0.63841100	3.02284000
H	-5.84353700	2.02534300	1.91616000
H	-7.19670700	1.43458600	2.91685100
H	4.83191100	-2.12253000	-0.13089300
O	4.44041400	-1.45530200	1.79234500
H	5.39272200	-1.44153100	1.96500500

C-7

G= -1429.060598 au

0 2	x	y	z
C	0.74782000	1.58377800	-1.22073700
C	2.83420500	0.43868800	-0.80968600
C	2.17863300	-0.65834600	-0.21234500
C	0.71853000	-0.68083500	-0.12715400
C	0.01165200	0.43692500	-0.71275700
H	4.67081300	1.33812400	-1.41006600
C	4.18328500	0.49153200	-0.94221300
C	2.95316300	-1.75103400	0.28024400
C	4.29690900	-1.74996400	0.19137200
C	5.04801300	-0.63560900	-0.47937600
H	2.42187800	-2.57765900	0.73460300

H	4.89813200	-2.57032800	0.57021200
O	2.12102800	1.51125600	-1.26654600
O	0.28389500	2.63053200	-1.60826300
O	0.16045000	-1.65393900	0.41305600
C	-1.41333300	0.44723600	-0.77509900
C	-2.18816300	1.48273200	-1.53336200
H	-2.24020500	2.41286700	-0.96418000
H	-1.67683200	1.71179000	-2.46470800
H	-3.19581300	1.12820100	-1.73600700
O	6.10032300	-0.14612700	0.40376900
C	5.77287400	0.41902800	1.58301600
C	7.00277800	0.81211700	2.34676200
H	7.63608100	1.44600700	1.72570400
H	6.71490300	1.33610400	3.25411200
H	7.56982700	-0.08565500	2.59802900
O	4.63735300	0.57191300	1.95090100
N	-2.08208400	-0.53310300	-0.18813900
H	-1.48658700	-1.25867400	0.23585100
C	-3.49686000	-0.68329800	-0.08526200
C	-4.07710200	-1.87138000	-0.50036500
C	-4.26766300	0.33129100	0.49129800
C	-5.45218900	-2.05278800	-0.35887300
H	-3.46299000	-2.64817800	-0.93858000
C	-5.63497700	0.14964400	0.61767400
C	-6.23151400	-1.05107600	0.19001000
H	-5.92860000	-2.97070800	-0.68033200
H	-3.78886700	1.23689200	0.83996400
O	-6.50697300	1.04457600	1.14501800
O	-7.56961100	-1.22259000	0.32253000
H	-7.94337600	-0.42293300	0.71667800
C	-5.99198700	2.30162900	1.56773300
H	-5.27202500	2.16892900	2.37856500
H	-5.52010100	2.82256000	0.73102600
H	-6.84562700	2.87222400	1.92308600
O	5.74628400	-1.09879000	-1.60022900
H	6.27753700	-1.86411700	-1.34416500

C-8

G= -1429.057416 au

0 2	x	y	z
C	0.59817900	1.77760200	-0.80708000
C	2.64965000	0.56148600	-0.51343300
C	2.03497800	-0.62418000	-0.26256200
C	0.56288700	-0.68318000	-0.29763000

C	-0.13778100	0.54694300	-0.60563700
C	4.13380700	0.77253200	-0.48077000
C	2.81155800	-1.78383700	0.01837900
C	4.21399600	-1.70286700	0.02864600
C	4.83690800	-0.51731000	-0.21341400
H	2.30223900	-2.71620100	0.21532500
H	4.81122300	-2.58488000	0.22550000
O	1.98621100	1.69982000	-0.78444900
O	0.16401900	2.88985800	-0.98784500
O	-0.00223600	-1.76460400	-0.07259500
C	-1.56427200	0.57728700	-0.67774600
C	-2.32794600	1.79268800	-1.10691300
H	-2.30556200	2.55090000	-0.32165300
H	-1.84791800	2.23215600	-1.97831000
H	-3.35898900	1.53739400	-1.33631400
O	6.21027100	-0.40284700	-0.28109400
C	6.96108900	-0.58551200	0.84566800
C	8.41038100	-0.36931900	0.54326000
H	8.56320900	0.67666500	0.27179900
H	9.00547000	-0.61745000	1.41765400
H	8.70511200	-0.98293500	-0.30813500
O	6.48541200	-0.86994800	1.90729400
N	-2.24057500	-0.52309500	-0.39389700
H	-1.65352500	-1.34283900	-0.18592100
C	-3.65871900	-0.67014800	-0.31038100
C	-4.28442500	-1.64431200	-1.07060600
C	-4.38452900	0.12422500	0.58281100
C	-5.66242700	-1.82589100	-0.95630700
H	-3.70427200	-2.25351900	-1.75240000
C	-5.75464800	-0.05204200	0.68197100
C	-6.39782500	-1.03543500	-0.09204900
H	-6.17545200	-2.57866000	-1.54186300
H	-3.86859600	0.85864900	1.18725100
O	-6.58679800	0.64950100	1.49106900
O	-7.73819600	-1.20663500	0.01472700
H	-8.07970900	-0.56403900	0.65130000
C	-6.02737700	1.70769800	2.26033200
H	-5.29594800	1.32012000	2.97319600
H	-5.55789900	2.44743500	1.60723900
H	-6.85683300	2.16184200	2.79536000
H	4.46233100	1.17773500	-1.44913700
O	4.49519900	1.69843200	0.54648700
H	4.34718800	2.59211900	0.21840700

C-9

G= -1429.055737 au

0 2	x	y	z
C	0.73550200	1.72882400	-1.11951700
C	2.63916300	0.78416300	0.03441300
C	2.10526900	-0.58628800	-0.23156900
C	0.64799600	-0.68348600	-0.41015500
C	-0.04037200	0.54001500	-0.76657400
H	4.57707000	1.87036900	-0.02676200
C	4.12976800	0.88385500	-0.07166300
C	2.92580100	-1.67925100	-0.28785700
C	4.31756300	-1.53732200	-0.22204600
C	4.88128500	-0.22995500	-0.13965200
H	2.48373200	-2.65805500	-0.42991600
H	4.97271800	-2.39602200	-0.28439700
O	2.09082300	1.71380700	-0.89878100
O	0.30761200	2.73553800	-1.63515900
O	0.09323500	-1.78649400	-0.27271600
C	-1.45999600	0.58326300	-0.82983400
C	-2.22128300	1.82200900	-1.19585600
H	-1.98974400	2.63075100	-0.50168000
H	-1.89754600	2.15468400	-2.18044500
H	-3.29089900	1.63744400	-1.20361400
O	6.26233700	-0.10129300	-0.22263000
C	6.99679900	-0.55224700	0.83273100
C	8.45908700	-0.37942500	0.57179600
H	8.66738600	0.66679200	0.34539900
H	9.02460400	-0.69873700	1.44255900
H	8.74014700	-0.97308900	-0.29938500
O	6.49041600	-1.01591900	1.81613300
N	-2.14864200	-0.51990000	-0.56399700
H	-1.57306600	-1.35580300	-0.40588400
C	-3.56325000	-0.64780200	-0.41263300
C	-4.25193800	-1.54056600	-1.21700200
C	-4.22187700	0.07463400	0.58783400
C	-5.62465100	-1.71181100	-1.03794900
H	-3.72435200	-2.09514500	-1.98290900
C	-5.58727100	-0.09020200	0.75251000
C	-6.29354400	-0.99059300	-0.06622600
H	-6.18549200	-2.40237800	-1.65557400
H	-3.65675800	0.74657200	1.22081600
O	-6.35649500	0.54478000	1.67209300
O	-7.62880300	-1.15250800	0.10512800
H	-7.91741700	-0.57748900	0.82660300

C	-5.71362900	1.45197300	2.55952500
H	-4.95795300	0.93418200	3.15509300
H	-5.25281800	2.27173500	2.00321100
H	-6.49251600	1.84005100	3.20996800
O	2.20920600	1.13907600	1.33163200
H	2.60422300	1.98883100	1.56764200

C-10

G= -1429.044395 au

0 2	x	y	z
C	0.66222800	2.03751700	-0.51341100
C	2.76518400	0.95817700	-0.16643100
C	2.19767300	0.13936800	0.93893600
C	0.72719000	-0.19637700	0.61562600
C	-0.00047200	0.79606600	-0.12901300
H	4.35703800	1.39699900	-1.50457800
C	3.98202300	0.73640200	-0.73294300
C	3.01233200	-1.08706800	1.20235300
C	4.22758000	-1.28895800	0.63986900
C	4.73576700	-0.36814600	-0.31129500
H	2.59528300	-1.79937700	1.90344100
H	4.81435500	-2.16397000	0.89148500
O	2.04105600	2.04713000	-0.54947900
O	0.13067000	3.07450900	-0.82373600
O	0.25338700	-1.24325000	1.06092400
C	-1.37851500	0.62492400	-0.43376500
C	-2.13208600	1.57414000	-1.31660200
H	-2.44012100	2.45458100	-0.74944700
H	-1.48641700	1.92197700	-2.11884700
H	-3.01291300	1.08911400	-1.73056600
O	5.93656500	-0.59580000	-0.94925300
C	7.07180000	-0.72825500	-0.20083200
C	8.23924300	-1.06220400	-1.07354400
H	8.33154200	-0.31673600	-1.86386800
H	9.14387300	-1.09263100	-0.47284300
H	8.06825900	-2.03136700	-1.54489200
O	7.08090300	-0.58288400	0.98863000
N	-2.01424100	-0.44269300	0.02591000
H	-1.43554100	-1.09971200	0.55773400
C	-3.39511300	-0.76529900	-0.14797800
C	-3.73622400	-1.98233800	-0.71513700
C	-4.37831500	0.12070400	0.30295300
C	-5.08089700	-2.32373600	-0.85430100
H	-2.96078100	-2.65750500	-1.05496500

C	-5.71258700	-0.21780700	0.14804400
C	-6.06617400	-1.44917400	-0.43416300
H	-5.37304500	-3.26751400	-1.29791600
H	-4.08687400	1.05113900	0.77239700
O	-6.77107400	0.54237600	0.52350000
O	-7.37392100	-1.77564500	-0.57701600
H	-7.91029100	-1.04894900	-0.23259200
C	-6.50195000	1.81585200	1.09797000
H	-5.94958800	1.70550000	2.03401800
H	-5.93619000	2.43880900	0.40091300
H	-7.47032200	2.26827900	1.29326300
O	2.15689000	1.00236700	2.10173700
H	1.78348200	0.49705600	2.83540700

C-1’

G= -1429.053718 au

0 2	x	y	z
C	-0.81278000	-1.84503000	0.37777800
C	-2.91393000	-0.77685400	-0.07996600
C	-2.35275300	0.49202400	-0.18475800
C	-0.91567800	0.66345800	0.02689600
C	-0.16998800	-0.52558700	0.45964300
H	-4.70548200	-1.97018900	-0.16877400
C	-4.27769400	-0.97975700	-0.25023300
C	-3.18138700	1.58306400	-0.47951700
C	-4.53688600	1.40769700	-0.65607900
C	-5.06600700	0.11959000	-0.53332200
H	-2.73122700	2.56445500	-0.56402800
H	-5.18824500	2.24035100	-0.88593600
O	-2.16302500	-1.89007900	0.17208500
O	-0.24169000	-2.89747100	0.47280000
O	-0.34602700	1.74020700	-0.13506100
C	1.30667900	-0.35952700	0.82325400
C	1.90182000	-1.49780600	1.64010000
H	1.26573800	-1.69708200	2.50262700
H	2.01139200	-2.40522200	1.05584300
H	2.87884400	-1.16645600	1.99236400
O	-6.40701900	-0.11143700	-0.76659400
C	-7.32862900	0.48518000	0.05010000
C	-8.71502800	0.16353000	-0.40668100
H	-8.83801500	-0.91809300	-0.46862700
H	-9.43380400	0.58899400	0.28781700
H	-8.86603400	0.57608500	-1.40545800
O	-7.01501000	1.15671600	0.99051400

N	1.95381100	-0.22980400	-0.48104900
H	1.63235000	0.62350300	-0.92964400
C	3.38630900	-0.31983400	-0.51201500
C	3.98662400	-1.52183300	-0.85672100
C	4.17863700	0.79299700	-0.19818100
C	5.37696600	-1.63308500	-0.87774100
H	3.36498500	-2.37126900	-1.11221100
C	5.56102900	0.68580800	-0.23713500
C	6.16430300	-0.53783400	-0.57189200
H	5.86014200	-2.56570800	-1.14304800
H	3.69980700	1.72464000	0.07455900
O	6.44381300	1.68495300	0.03337500
O	7.51980100	-0.63804300	-0.60403500
H	7.89246700	0.22098600	-0.36534700
C	5.91424900	2.96128400	0.36643300
H	5.30913300	3.35247400	-0.45513600
H	5.31193500	2.90321300	1.27635800
H	6.77042400	3.60934000	0.53384600
O	1.45541900	0.78146300	1.65582200
H	1.08368500	1.54324500	1.19111900

C-1’’

G= -1429.060396 au

0 2	x	y	z
C	0.72098200	1.75853300	-0.41080100
C	2.90653400	0.74608100	-0.35788000
C	2.39330600	-0.53100500	-0.17960300
C	0.93301800	-0.72863600	-0.12290400
C	0.10952400	0.44698500	-0.30210600
H	4.66874500	1.97336300	-0.57176200
C	4.27897100	0.97338800	-0.43278000
C	3.27821600	-1.60995600	-0.07267200
C	4.64280700	-1.41385600	-0.14721500
C	5.12092500	-0.11494400	-0.32678900
H	2.86434700	-2.60130100	0.06309800
H	5.33948000	-2.23889200	-0.07259800
O	2.10047000	1.83429700	-0.45849600
O	0.16243200	2.82972600	-0.45881700
O	0.48762900	-1.87276300	0.05644200
C	-1.31802500	0.33881600	-0.35708600
C	-2.19993100	1.51229100	-0.66125100
H	-2.20445300	2.19032200	0.19435600
H	-1.79035400	2.06943200	-1.50103300
H	-3.21517200	1.20515900	-0.88372900

O	6.48320400	0.10588000	-0.46807800
C	7.27256700	-0.02483100	0.63641100
C	8.70868700	0.20246600	0.28551200
H	8.81850400	1.17825800	-0.18865000
H	9.31552200	0.14792600	1.18485500
H	9.02738800	-0.55623700	-0.43084900
O	6.82998900	-0.28337800	1.71986200
N	-1.86704300	-0.83946900	-0.15111400
H	-1.20864500	-1.60853900	0.03077600
C	-3.29625300	-1.22951900	-0.14967200
C	-3.89902200	-1.13171600	-1.52141400
C	-4.06692100	-0.49805600	0.90761000
C	-5.09819200	-0.53086500	-1.74629400
H	-3.34494500	-1.61221700	-2.31862500
C	-5.25700600	0.08591700	0.63521300
C	-5.79928700	0.08900400	-0.69612000
H	-5.53399100	-0.50680000	-2.73818300
H	-3.63852600	-0.50912200	1.90113500
O	-6.05758300	0.72809300	1.51732000
O	-6.97971700	0.68065300	-0.93362100
H	-7.31177000	1.05524500	-0.10408200
C	-5.61705000	0.80708700	2.86861600
H	-5.50311900	-0.19411600	3.29007600
H	-4.66900800	1.34678100	2.92738000
H	-6.38923800	1.35061300	3.40564700
O	-3.15160200	-2.61123700	0.21296800
H	-4.03714900	-2.98685300	0.27669800

C-2’’

G= -1429.052314 au

0 2	x	y	z
C	0.98007200	1.95176500	-0.61632200
C	3.06379400	0.75997300	-0.42187700
C	2.43758700	-0.46990600	-0.27359400
C	0.96675600	-0.54763400	-0.31824900
C	0.25669100	0.69438300	-0.53895900
H	4.92805800	1.83972100	-0.52684600
C	4.45156400	0.87542800	-0.40741200
C	3.22414800	-1.61548000	-0.10528300
C	4.60183800	-1.53063000	-0.09019900
C	5.19497200	-0.27579700	-0.24308100
H	2.72468600	-2.56972100	0.00644200
H	5.22145300	-2.40941400	0.03242300
O	2.35941300	1.91111600	-0.58061200

O	0.51180300	3.06248800	-0.70229800
O	0.41343900	-1.64938600	-0.18369200
C	-1.16220000	0.70858700	-0.66176700
C	-1.94384100	1.93661100	-1.01581600
H	-1.96591200	2.62823600	-0.17146700
H	-1.45427000	2.46007300	-1.83403800
H	-2.95844400	1.66994000	-1.30576900
O	6.57474800	-0.16082800	-0.30414900
C	7.29988900	-0.44011800	0.81720700
C	8.76322800	-0.29558200	0.54368500
H	8.97397900	0.73733500	0.26212200
H	9.32782900	-0.56389700	1.43206500
H	9.04298600	-0.93458000	-0.29436200
O	6.78924200	-0.74231300	1.85831200
N	-1.83586600	-0.42175000	-0.49827900
H	-1.24800200	-1.24950700	-0.32823400
C	-3.25437300	-0.57354300	-0.51608900
C	-3.85333400	-1.35339800	-1.44827000
C	-3.99039100	0.00467400	0.66235200
C	-5.25640000	-1.56120600	-1.43971100
H	-3.25235000	-1.80630300	-2.22840800
C	-5.46885900	-0.15351100	0.51150000
C	-6.04092600	-0.94142100	-0.46632300
H	-5.73294900	-2.17061000	-2.19557900
O	-6.31652100	0.36722000	1.42384400
O	-7.39098000	-1.11562500	-0.50119900
H	-7.78064000	-0.61893400	0.23015000
C	-5.88376000	1.47932800	2.21199000
H	-5.02676000	1.20301200	2.82672800
H	-5.63642400	2.32525000	1.56707600
H	-6.72797400	1.73757300	2.84523800
H	-3.74027700	1.06757500	0.78083400
O	-3.61742900	-0.66831300	1.88105800
H	-2.75237700	-0.34349800	2.15677500

C-3’’

G= -1429.059511 au

0 2	x	y	z
C	1.05506400	1.93795100	-0.47598000
C	3.14960700	0.75440900	-0.36061800
C	2.53358000	-0.47820800	-0.19445800
C	1.06254500	-0.56009900	-0.18339500
C	0.33995400	0.67647100	-0.38901700
H	5.00547200	1.84118600	-0.52786900

C	4.53666400	0.87468500	-0.39628500
C	3.32931200	-1.62197300	-0.06006400
C	4.70635100	-1.53231600	-0.09568700
C	5.28910700	-0.27473800	-0.26411500
H	2.83730500	-2.57831500	0.06577400
H	5.33354800	-2.40928700	-0.00126400
O	2.43545900	1.90299800	-0.48707700
O	0.58113100	3.04822100	-0.52897600
O	0.51931900	-1.66408100	-0.02144000
C	-1.08433300	0.68314800	-0.47876300
C	-1.86666300	1.90216400	-0.86383100
H	-1.92199600	2.59720700	-0.02373000
H	-1.35428900	2.42246200	-1.66921200
H	-2.87269900	1.62949400	-1.17088700
O	6.66604900	-0.15653500	-0.37224600
C	7.42605900	-0.41029000	0.73182200
C	8.87990400	-0.26475000	0.41201900
H	9.07870400	0.76637300	0.11551400
H	9.47179600	-0.52335400	1.28545300
H	9.13630700	-0.91018700	-0.42846700
O	6.94851800	-0.69389600	1.79373800
N	-1.73657100	-0.44974300	-0.26803200
H	-1.12772300	-1.26565400	-0.10267200
C	-3.14812100	-0.65870000	-0.25300300
C	-3.65644000	-1.68228900	-1.10210600
C	-3.94260100	0.06015900	0.57500800
C	-5.03563300	-1.92043000	-1.15103500
H	-2.97613200	-2.23636200	-1.73503900
C	-5.41588800	-0.19314800	0.65897100
C	-5.88907500	-1.18717700	-0.37625400
H	-5.44663900	-2.65131500	-1.83647200
H	-3.52446900	0.78346400	1.26460600
O	-6.22526300	0.94529500	0.43094100
O	-7.22215500	-1.35338200	-0.45830400
H	-7.65631100	-0.55060300	-0.13204300
C	-5.94054600	2.08988800	1.23187900
H	-5.84922800	1.81574900	2.28442300
H	-5.02518700	2.57964900	0.89052500
H	-6.77991600	2.76950100	1.10356200
O	-5.64770200	-0.68176600	1.97156700
H	-6.58847400	-0.87519800	2.07786800

C-4''

G= -1429.065852 au

0 2	x	y	z
C	1.12531700	2.01710000	-0.42872700
C	3.18445000	0.77410900	-0.34691300
C	2.53359300	-0.44919600	-0.26297300
C	1.06216700	-0.49230400	-0.28536100
C	0.37449800	0.77252100	-0.42351300
H	5.07010700	1.81901300	-0.41506600
C	4.57441300	0.85944400	-0.34688000
C	3.29741500	-1.61954600	-0.17628600
C	4.67672600	-1.56393400	-0.17697900
C	5.29439600	-0.31494500	-0.26234500
H	2.77844500	-2.56791200	-0.11462300
H	5.27978400	-2.46077400	-0.11735100
O	2.50313700	1.94657700	-0.42488800
O	0.67764500	3.13954100	-0.42018800
O	0.48562000	-1.59030800	-0.20686000
C	-1.04685600	0.82881700	-0.51935300
C	-1.78997100	2.07639400	-0.88950900
H	-1.89513500	2.74628100	-0.03355700
H	-1.22373300	2.61912300	-1.64214200
H	-2.77437200	1.82510300	-1.27817000
O	6.67638300	-0.22844800	-0.33063700
C	7.39675100	-0.53569100	0.78616300
C	8.86252200	-0.41329700	0.51465600
H	9.09043700	0.61754500	0.23905500
H	9.42203000	-0.69617000	1.40173600
H	9.13242200	-1.05205800	-0.32684700
O	6.88049900	-0.84379400	1.82287100
N	-1.73156400	-0.30217900	-0.35692400
H	-1.12186300	-1.13545400	-0.27738800
C	-3.11118600	-0.51107400	-0.26625600
C	-3.62161800	-1.72663300	-0.80804900
C	-3.97183600	0.39457900	0.40236000
C	-4.93509900	-2.02818700	-0.73139400
H	-2.93506000	-2.39529800	-1.31528300
C	-5.29962400	0.11150800	0.51240200
C	-5.92325000	-1.16453500	-0.00868300
H	-5.33527500	-2.93573700	-1.16991400
H	-3.56630300	1.29323800	0.84337800
O	-6.22605700	0.89327600	1.07603900
O	-6.97700000	-0.87959900	-0.89984100
H	-7.59103100	-0.28834900	-0.44383600
C	-5.81937200	2.16315300	1.58030000
H	-5.10305500	2.03622600	2.39472100

H	-5.37710400	2.76328600	0.78141100
H	-6.72149900	2.64084700	1.95115600
O	-6.43749700	-1.82891700	1.13441600
H	-6.85031700	-2.64999000	0.83793900

C-5''

G= -1429.054168 au

0 2	x	y	z
C	1.09564100	1.90786100	-0.85538000
C	3.14878300	0.69764700	-0.50732000
C	2.49096700	-0.47252800	-0.15494700
C	1.01768000	-0.50780000	-0.15635500
C	0.33810800	0.70552900	-0.55643200
H	5.04232400	1.68786800	-0.80580400
C	4.53952200	0.77052400	-0.52908800
C	3.24721400	-1.60085200	0.18427200
C	4.62685500	-1.55771300	0.16727900
C	5.25234200	-0.36226400	-0.19152100
H	2.72142200	-2.50801300	0.45446800
H	5.22319600	-2.42397800	0.42308700
O	2.47447100	1.82851800	-0.84114800
O	0.65915200	3.00466800	-1.11403400
O	0.43715000	-1.55571800	0.16631500
C	-1.08561700	0.74846400	-0.63942800
C	-1.83862500	1.93114300	-1.16812400
H	-1.79970800	2.75273100	-0.45071200
H	-1.36158100	2.28842900	-2.07822500
H	-2.87449700	1.66964700	-1.36862100
O	6.63393000	-0.29991800	-0.28574700
C	7.36863800	-0.41992500	0.85741000
C	8.83132800	-0.38679000	0.54687000
H	9.06672100	0.51843100	-0.01338400
H	9.39977900	-0.41937000	1.47194100
H	9.08337200	-1.24390300	-0.07954300
O	6.86654700	-0.53082500	1.94010200
N	-1.77889400	-0.31761900	-0.27598900
H	-1.21108400	-1.12673300	0.00880800
C	-3.20477600	-0.43843200	-0.22822900
C	-3.80525100	-1.38533500	-0.97890500
C	-3.91367400	0.42362900	0.67180300
C	-5.27718900	-1.62771500	-0.90491500
H	-3.22621100	-2.01311000	-1.64483200
C	-5.29503700	0.30958800	0.74154600
C	-5.96281900	-0.63572000	-0.02568300

H	-5.71729600	-1.56124000	-1.91082900
H	-3.36253800	1.12976800	1.27609700
O	-6.11316100	1.06404700	1.52501000
O	-7.29937400	-0.75639800	0.01765200
H	-7.65598200	-0.10358300	0.63848400
C	-5.51927200	2.11797700	2.27335300
H	-4.81224500	1.72067500	3.00530300
H	-5.01201700	2.82131700	1.60825600
H	-6.33510200	2.61920600	2.78716800
O	-5.48726100	-2.96697100	-0.42269800
H	-6.41682400	-3.18777100	-0.55058400

C-6’’

G= -1429.059789 au

0 2	x	y	z
C	0.98883600	2.07996500	-0.44643500
C	3.04574900	0.83360300	-0.35300400
C	2.39213900	-0.38365500	-0.22000700
C	0.91908500	-0.42314800	-0.22125500
C	0.23467500	0.83866300	-0.40219700
H	4.93330800	1.86935000	-0.48096500
C	4.43593000	0.91404200	-0.37536600
C	3.15349400	-1.55298700	-0.10604600
C	4.53299600	-1.50271000	-0.12831000
C	5.15351800	-0.25948600	-0.26306600
H	2.63269500	-2.49708900	-0.00626500
H	5.13369300	-2.39957100	-0.04848800
O	2.36695500	2.00560300	-0.45665000
O	0.54576200	3.20452600	-0.45926000
O	0.34179300	-1.51418500	-0.09210100
C	-1.18693600	0.89714000	-0.50016900
C	-1.92135900	2.13402900	-0.91995600
H	-2.04545500	2.82455700	-0.08303100
H	-1.34304700	2.65877100	-1.67546100
H	-2.89911500	1.86899000	-1.31665400
O	6.53472700	-0.17998500	-0.35427900
C	7.27158100	-0.45975700	0.75886600
C	8.73334100	-0.35048000	0.46121200
H	8.96203400	0.67443100	0.16482300
H	9.30561400	-0.62112000	1.34398700
H	8.98640400	-1.00463000	-0.37357700
O	6.77113400	-0.73749100	1.81181700
N	-1.88224300	-0.21720900	-0.28701600
H	-1.29817400	-1.06074400	-0.15555900

C	-3.26431900	-0.42161000	-0.21374900
C	-3.68611000	-1.78305800	-0.70100200
C	-4.13442500	0.46626400	0.37893800
C	-5.16127000	-1.99800800	-0.61412700
H	-3.34477200	-1.90919900	-1.73510200
C	-5.49161400	0.15943900	0.45721100
C	-5.99204100	-1.09322600	-0.06145500
H	-5.55769400	-2.93053800	-0.99759500
H	-3.76027100	1.38377500	0.81143400
O	-6.44127500	0.93419300	1.00692500
O	-7.32500100	-1.32810600	0.02928600
H	-7.74832800	-0.56246600	0.43962300
C	-6.06637000	2.20965300	1.52397400
H	-5.35528500	2.09225500	2.34389600
H	-5.63317000	2.82519400	0.73252800
H	-6.98251300	2.66353400	1.89013100
O	-2.95113000	-2.80258100	0.00493100
H	-3.29533500	-2.83888100	0.90571200

Cartesian coordinates (Transition states-A-3OH, RAF mechanisam)

C-3

G= -1429.028633 au

0 2	x	y	z
C	0.99087100	2.11145500	-0.25782900
C	2.93866600	0.71822500	-0.31454500
C	2.28414300	-0.37872700	0.23250700
C	0.86823600	-0.28773000	0.60201000
C	0.18879000	1.00591700	0.33059600
H	4.77471900	1.52195100	-1.10386200
C	4.27900100	0.65967400	-0.67786400
C	3.00220900	-1.56780100	0.41900900
C	4.33180600	-1.65581000	0.06194500
C	4.95346800	-0.53033300	-0.48311500
H	2.48512700	-2.41806500	0.84589400
H	4.89084500	-2.57229200	0.19592700
O	2.31196300	1.91472300	-0.51620400
O	0.55709200	3.21558200	-0.45942500
O	0.28095200	-1.23673600	1.11569300
C	-1.19904500	0.94087700	-0.09781900
C	-1.79708600	1.86930100	-1.10899300
H	-2.13122900	2.79560600	-0.63520100
H	-1.06136800	2.13745500	-1.86245400
H	-2.65105800	1.39310400	-1.58748900

O	6.26520300	-0.59161800	-0.91806000
C	7.24849000	-0.85622200	-0.00731400
C	8.57899700	-0.93571100	-0.68529400
H	8.77159500	-0.00325700	-1.21701900
H	9.35353800	-1.11616700	0.05463700
H	8.56058700	-1.74267900	-1.41916500
O	7.02664200	-0.98939500	1.16222000
N	-1.94768800	-0.01384900	0.42920600
H	-1.44948000	-0.67454000	1.03110700
C	-3.32998600	-0.26969700	0.18988200
C	-3.71992200	-1.55172800	-0.16489400
C	-4.27505500	0.74167400	0.37689700
C	-5.07253000	-1.83511600	-0.35983200
H	-2.97599500	-2.32721600	-0.29919700
C	-5.61242600	0.46256900	0.17249600
C	-6.01734300	-0.83266100	-0.20063900
H	-5.37177000	-2.83599300	-0.64021100
H	-3.98243900	1.73535500	0.69271000
O	-6.53784400	1.43627900	0.34643100
O	-7.35727600	-0.96387900	-0.36427400
O	0.12428600	1.58962900	2.04431300
H	-0.20777500	2.49133700	1.93142100
H	-7.41209000	1.06057400	0.17475900
C	-7.86091400	-2.24978800	-0.70649200
H	-7.61809500	-2.97584500	0.07295000
H	-8.93900800	-2.13906200	-0.78456000
H	-7.45265000	-2.58019800	-1.66450000

C-5

G= -1429.024420 au

0 2	x	y	z
C	0.83081300	2.22395700	-0.13812500
C	2.87175100	0.95770100	-0.28027800
C	2.20750800	-0.25020300	-0.31706400
C	0.74620900	-0.28305400	-0.18287300
C	0.06781300	0.98917000	-0.19005800
H	4.77648900	1.97589700	-0.30020500
C	4.26641600	1.02213600	-0.34247200
C	2.95472700	-1.45467500	-0.39707100
C	4.35139400	-1.39550700	-0.53668200
C	4.97774100	-0.16220200	-0.46991100
H	4.93086700	-2.29989700	-0.66639500
O	2.21088900	2.13757900	-0.17736400
O	0.40020000	3.34748300	-0.03578700

O	0.18174200	-1.38659200	-0.07347200
C	-1.35972500	1.05535300	-0.21168100
C	-2.10865000	2.33583100	-0.41662300
H	-2.10143600	2.92704700	0.50123300
H	-1.61084500	2.93137300	-1.17800500
H	-3.13609000	2.13368100	-0.70830600
O	6.35003400	-0.06090600	-0.61662100
C	7.14953900	-0.63073500	0.33492200
C	8.59053500	-0.45536100	-0.02310700
H	8.81434400	0.60873300	-0.10850400
H	9.21330900	-0.91191800	0.74073900
H	8.77952300	-0.91636000	-0.99342200
O	6.70812800	-1.17547100	1.30579500
N	-2.04747800	-0.06752200	-0.10254200
H	-1.47352100	-0.91881900	-0.03746000
C	-3.46809900	-0.21966700	-0.08612600
C	-4.06204000	-1.04245400	-1.02871100
C	-4.22906200	0.40112000	0.90641400
C	-5.44318500	-1.24498400	-0.99995400
H	-3.45678100	-1.52034300	-1.78867400
C	-5.59690700	0.20666600	0.92950200
C	-6.21109200	-0.62141300	-0.02864300
H	-5.90281600	-1.88533800	-1.74054300
H	-3.76646200	1.02523500	1.66159400
O	-6.34748700	0.80548900	1.88537900
O	-7.55579500	-0.72702900	0.11698800
H	2.42840300	-2.36094300	-0.66600300
O	2.79710100	-2.03096700	1.53927700
H	1.83427600	-2.14898500	1.47086200
H	-7.26990000	0.54703200	1.75472900
C	-8.25859800	-1.55253500	-0.80437500
H	-8.14071200	-1.17562500	-1.82293200
H	-7.90306100	-2.58376100	-0.74408500
H	-9.30426000	-1.50591100	-0.51289000

C-6

G= -1429.023727 au

0 2	x	y	z
C	0.70080200	2.21498500	-0.09757700
C	2.77047200	0.98323400	-0.16156800
C	2.11871600	-0.25118400	-0.09237300
C	0.64326800	-0.29651900	-0.06352900
C	-0.04585400	0.97203500	-0.12767300
H	4.63487500	2.03646300	-0.40526300

C	4.15734700	1.07383800	-0.27894400
C	2.87729200	-1.40676200	-0.08859100
C	4.28750600	-1.35712000	-0.06656300
C	4.89374500	-0.08843600	-0.28026600
H	2.37537100	-2.36471100	-0.04082100
O	2.08775100	2.14635600	-0.14633700
O	0.26387100	3.33689400	-0.02413300
O	0.07596300	-1.39699400	-0.00477300
C	-1.47188900	1.02692700	-0.19652600
C	-2.22487400	2.29968900	-0.43602200
H	-2.23940700	2.90743900	0.47066000
H	-1.71615000	2.88483600	-1.19854100
H	-3.24466100	2.08630300	-0.74559400
O	6.22660900	0.05828200	-0.59798000
C	7.22435200	-0.68394900	-0.04547200
C	8.52492400	-0.38535000	-0.71751300
H	8.70290000	0.69001100	-0.71230900
H	9.32640200	-0.90904500	-0.20447900
H	8.46791900	-0.71133300	-1.75760400
O	7.05015400	-1.45584300	0.85713800
N	-2.15612800	-0.09982700	-0.09935700
H	-1.57674600	-0.94653700	-0.01972600
C	-3.57531600	-0.26035500	-0.10117100
C	-4.15080600	-1.10690200	-1.03435500
C	-4.35482200	0.37445100	0.86798800
C	-5.53055200	-1.32051400	-1.01912100
H	-3.53180000	-1.59596300	-1.77590400
C	-5.72124700	0.16971300	0.87715100
C	-6.31618200	-0.68360200	-0.07097400
H	-5.97499200	-1.98082000	-1.75148400
H	-3.90748800	1.01792500	1.61607400
O	-6.48921400	0.78225700	1.81040200
O	-7.66179500	-0.79803800	0.06040200
H	4.85452000	-2.25611400	-0.26277400
O	4.41745200	-1.56995600	1.91996300
H	5.38653000	-1.62226500	1.89439800
H	-7.40766500	0.51330300	1.67327200
C	-8.34516300	-1.65427400	-0.84731300
H	-7.97823200	-2.67935000	-0.75753600
H	-9.39439200	-1.61280500	-0.56808000
H	-8.22072400	-1.30165800	-1.87377400

C-7

G= -1429.024210 au

0 2	x	y	z
C	0.83498000	1.97024300	-0.55432300
C	2.84414500	0.65183300	-0.39782000
C	2.14520800	-0.51487600	-0.09771300
C	0.66979600	-0.48821500	-0.05547200
C	0.03468400	0.77601900	-0.34069100
H	4.76741400	1.57927500	-0.67311200
C	4.22673000	0.66655600	-0.45853700
C	2.85387100	-1.69960300	0.16416800
C	4.22519500	-1.72097600	0.10934500
C	4.91751600	-0.54401200	-0.27667500
H	2.29000800	-2.58747400	0.41962700
H	4.79654900	-2.61653800	0.31738700
O	2.20754500	1.83236800	-0.61532300
O	0.43790700	3.10338100	-0.68255000
O	0.05973500	-1.53642800	0.20568700
C	-1.39031100	0.88210800	-0.38592000
C	-2.09958700	2.12878300	-0.81783300
H	-2.05061000	2.88266400	-0.02983900
H	-1.59887900	2.55023700	-1.68624400
H	-3.13921600	1.91522900	-1.05159300
O	6.29388000	-0.49381300	-0.11048800
C	6.74448800	-0.22399000	1.14876300
C	8.23952400	-0.20739600	1.17820600
H	8.61753900	-1.17784300	0.85425400
H	8.60699400	0.54555200	0.47991000
H	8.58005600	0.01344500	2.18576600
O	6.00388200	-0.03366600	2.07231600
N	-2.11574000	-0.18011100	-0.08127600
H	-1.56849400	-1.02628000	0.12766700
C	-3.53932300	-0.26789300	-0.00634800
C	-4.19318600	-1.22592700	-0.76329200
C	-4.24568200	0.55889900	0.86976200
C	-5.57970300	-1.35920500	-0.66492800
H	-3.62981200	-1.86331900	-1.43311000
C	-5.61843600	0.43369600	0.95906500
C	-6.29315600	-0.53090400	0.18781300
H	-6.08630000	-2.10767100	-1.25919400
H	-3.73627900	1.29203400	1.48359100
O	-6.31646600	1.23477000	1.79979000
O	-7.63615200	-0.54583400	0.37868500
O	5.14255300	-1.00491400	-2.22820500
H	4.22458400	-1.14852900	-2.50895800
H	-7.25269900	1.00242800	1.73388900

C	-8.39973900	-1.49514500	-0.35627800
H	-8.09458000	-2.51260700	-0.10121100
H	-9.43502500	-1.33704300	-0.06683000
H	-8.28620800	-1.32967000	-1.43013700

C-8

G= -1429.026553 au

0 2	x	y	z
C	0.77370300	2.01578800	-0.51323100
C	2.79687500	0.71854300	-0.42088300
C	2.12160100	-0.47608400	-0.25371800
C	0.64770800	-0.47910800	-0.22575800
C	-0.00683600	0.79873500	-0.40196300
C	4.20684600	0.77530600	-0.40787400
C	2.86056200	-1.65930000	-0.13747100
C	4.24846700	-1.65569600	-0.19013900
C	4.90298900	-0.44561800	-0.35028600
H	2.31896800	-2.58919900	-0.01558500
H	4.80948600	-2.57691600	-0.12048800
O	2.15917000	1.89907300	-0.55090700
O	0.37237600	3.15186200	-0.56994900
O	0.04979500	-1.55351700	-0.06626500
C	-1.43275900	0.89096500	-0.43174900
C	-2.15825600	2.16018800	-0.75893900
H	-2.11492100	2.85123800	0.08496200
H	-1.66695800	2.65381000	-1.59411400
H	-3.19651700	1.95293400	-1.00450300
O	6.26501700	-0.34944900	-0.48207600
C	7.09493800	-0.95366500	0.42855600
C	8.52204900	-0.74229300	0.03745300
H	8.72309900	0.32678400	-0.03941400
H	9.17294600	-1.19817000	0.77788800
H	8.69440500	-1.18705400	-0.94377400
O	6.68760800	-1.54458800	1.38481900
N	-2.14460400	-0.20029900	-0.20853700
H	-1.58662900	-1.05527800	-0.07804300
C	-3.56438900	-0.30734100	-0.10529200
C	-4.22696200	-1.23398400	-0.89329800
C	-4.25764400	0.46285800	0.83098700
C	-5.60844000	-1.39166600	-0.76487900
H	-3.67475200	-1.82872000	-1.60992900
C	-5.62566200	0.31441300	0.94990400
C	-6.30897600	-0.61899000	0.14865500
H	-6.12136500	-2.11539500	-1.38384600

H	-3.74102100	1.16762900	1.47145900
O	-6.30990700	1.06037100	1.85056000
O	-7.64540900	-0.66518000	0.37712700
H	4.71573800	1.67240000	-0.73380800
O	4.46531000	1.29896200	1.56006100
H	4.00852100	2.15154800	1.48516200
H	-7.24354300	0.81341400	1.80304300
C	-8.41464300	-1.59688700	-0.37423500
H	-8.07434400	-2.61728800	-0.18300700
H	-9.44069300	-1.48117200	-0.03618800
H	-8.34888800	-1.37574900	-1.44206400

C-9

G= -1429.023705 au

0 2	x	y	z
C	0.95877500	2.05204300	-0.00807100
C	2.91754400	0.70363800	-0.26937200
C	2.17885400	-0.41110800	-0.74458400
C	0.70636000	-0.35367600	-0.68965900
C	0.12764600	0.87566600	-0.20361400
H	4.88802900	1.58526500	-0.06795800
C	4.33163300	0.70238600	-0.35599700
C	2.85057000	-1.55350700	-1.14170100
C	4.24107200	-1.60223800	-1.11079000
C	4.95374000	-0.47283200	-0.70458700
H	2.27222000	-2.41125500	-1.46090100
H	4.78217200	-2.49751400	-1.38943200
O	2.32787100	1.91635700	-0.14884700
O	0.58722100	3.17697700	0.22216800
O	0.05233600	-1.35288400	-1.02579400
C	-1.27487900	0.97317400	0.04615800
C	-1.89682800	2.15012100	0.73408900
H	-1.23981700	2.50403700	1.52422800
H	-2.02223400	2.97533300	0.03043500
H	-2.86705100	1.87837700	1.14303700
O	6.33715000	-0.54580200	-0.65821800
C	6.88625800	-0.63419100	0.58985100
C	8.37624300	-0.70740700	0.51106400
H	8.75441600	0.18883800	0.01752900
H	8.78998500	-0.79087700	1.51185700
H	8.66390400	-1.56938800	-0.09210000
O	6.21367000	-0.65010200	1.58413400
N	-2.05192900	-0.04211700	-0.29096000
H	-1.55210200	-0.84990300	-0.68696700

C	-3.47062300	-0.13138200	-0.15958000
C	-4.30262000	0.78607200	-0.78080800
C	-3.99789300	-1.20646200	0.55791300
C	-5.68724800	0.65045100	-0.66966000
H	-3.88180300	1.60028000	-1.35735900
C	-5.36890200	-1.34555600	0.65978400
C	-6.22212400	-0.40954600	0.04723800
H	-6.33305500	1.37098400	-1.15320000
H	-3.34850200	-1.92711900	1.04001400
O	-5.89279800	-2.38294800	1.35624200
O	-7.54174300	-0.66450700	0.23252600
O	2.88542800	0.21478800	1.65527600
H	3.76470200	-0.17159100	1.80709100
H	-6.85681400	-2.31590400	1.32344600
C	-8.47855500	0.22218000	-0.36767100
H	-8.35681400	1.23310200	0.02815000
H	-8.35866200	0.22907700	-1.45344100
H	-9.46229900	-0.15891900	-0.10764300

C-10

G= -1429.026509 au

0 2	x	y	z
C	0.97383500	1.83980700	-0.41017500
C	2.94810000	0.47435200	-0.25239500
C	2.21491800	-0.59778600	0.31333400
C	0.72713800	-0.57758700	0.19865900
C	0.13618600	0.67170600	-0.21000300
H	4.84644900	1.23899400	-0.88443200
C	4.30849400	0.38678800	-0.49357300
C	2.88870800	-1.83903800	0.48346100
C	4.23634600	-1.93894600	0.25725100
C	4.93829400	-0.81773400	-0.22018100
H	2.30654600	-2.68739800	0.81921000
H	4.77377000	-2.86478600	0.41505700
O	2.34808900	1.65401700	-0.50291000
O	0.61916300	2.98583500	-0.52745400
O	0.09718000	-1.60780300	0.45401400
C	-1.27470000	0.79814300	-0.36513800
C	-1.92244200	2.03241000	-0.91591800
H	-1.98135200	2.80490300	-0.14669500
H	-1.31684100	2.43511500	-1.72386100
H	-2.92372800	1.80658400	-1.27472100
O	6.27153500	-1.00943000	-0.49055500
C	7.20939700	-0.10941300	-0.05289000

C	8.56670300	-0.50573800	-0.53661700
H	8.79285100	-1.51635900	-0.19488000
H	8.57151300	-0.51129700	-1.62743600
H	9.30474100	0.19621400	-0.15912500
O	6.93128100	0.83388800	0.62810500
N	-2.04717900	-0.23321000	-0.06349200
H	-1.54392900	-1.07814200	0.23084600
C	-3.47441700	-0.28254700	-0.08709500
C	-4.10131400	-1.26989500	-0.82921900
C	-4.21640200	0.61342100	0.68541800
C	-5.49439700	-1.36578100	-0.81844400
H	-3.51214800	-1.96013700	-1.41978800
C	-5.59497100	0.52501000	0.68744000
C	-6.24151800	-0.47110800	-0.06785100
H	-5.97975600	-2.13790000	-1.40000700
H	-3.73029800	1.37009800	1.28944300
O	-6.32616600	1.39025000	1.43085000
O	-7.59425200	-0.44678400	0.03399200
O	2.10884700	0.16286400	2.12928800
H	2.94494600	-0.17959000	2.48030300
H	-7.26137300	1.17138700	1.32028800
C	-8.33082300	-1.42660600	-0.68834400
H	-8.15347400	-1.32511900	-1.76155400
H	-8.05785000	-2.43162900	-0.35849200
H	-9.37804500	-1.23792300	-0.46866500

C-1’

G= -1429.025887 au

0 2	x	y	z
C	1.03650400	2.01547000	-0.48292000
C	3.05709900	0.71336800	-0.39837000
C	2.37948700	-0.49165400	-0.27836400
C	0.90987300	-0.50887000	-0.25624400
C	0.23812100	0.78455600	-0.38092000
H	4.96276600	1.71371100	-0.51932300
C	4.44706100	0.76715200	-0.42512400
C	3.11749800	-1.67916300	-0.18825600
C	4.49641900	-1.65564600	-0.21687700
C	5.14171800	-0.42244700	-0.33326100
H	2.57896000	-2.61434200	-0.10026300
H	5.07848600	-2.56590400	-0.15475100
O	2.40298300	1.90299800	-0.49415500
O	0.60676300	3.13603400	-0.54828400
O	0.30624900	-1.57750900	-0.13320800

C	-1.22267500	0.89224800	-0.41155500
C	-1.91461700	2.16142800	-0.83352900
H	-1.79740500	2.94090900	-0.08778800
H	-1.45458600	2.50889800	-1.75885000
H	-2.96811400	1.96932400	-1.01465300
O	6.52125100	-0.36599900	-0.42859200
C	7.26114200	-0.72688500	0.66119300
C	8.72173100	-0.64798800	0.35074400
H	8.97208700	0.36930400	0.04710300
H	9.29577800	-0.93165300	1.22821900
H	8.95013200	-1.31226800	-0.48366500
O	6.76157900	-1.04584200	1.70218500
N	-1.93131300	-0.23812000	-0.47542400
H	-1.39485100	-1.10048900	-0.38364500
C	-3.34173400	-0.34401500	-0.29345300
C	-4.05491600	-1.23270700	-1.08181100
C	-3.97871100	0.38579600	0.71372500
C	-5.42660200	-1.39801800	-0.87721300
H	-3.54827100	-1.79400900	-1.85703000
C	-5.33907300	0.23539900	0.90282000
C	-6.07118500	-0.66335300	0.10594400
H	-5.97748400	-2.09407200	-1.49546100
H	-3.41665200	1.06108200	1.34892800
O	-5.96805600	0.94520600	1.87223500
O	-7.39466700	-0.71697100	0.40922000
O	-0.78447300	1.28883800	1.45746800
H	-0.70642800	0.40822600	1.85644000
H	-6.90228300	0.69603400	1.87315500
C	-8.20637100	-1.61850400	-0.33272000
H	-8.20381300	-1.35436900	-1.39297600
H	-7.85567500	-2.64528900	-0.20421300
H	-9.21111700	-1.51930400	0.06894400

C-1’’

G= -1429.030584 au

0 2	x	y	z
C	1.05446200	2.06957500	-0.05101900
C	3.13071900	0.85944400	-0.22841900
C	2.49596000	-0.36881900	-0.34532200
C	1.02263800	-0.43417800	-0.31510500
C	0.31857400	0.82150600	-0.15313500
H	5.00251600	1.92904800	-0.15262900
C	4.51952800	0.96504600	-0.24557400
C	3.27396200	-1.52357500	-0.48757400

C	4.65236700	-1.44790600	-0.51183100
C	5.25400000	-0.19448400	-0.38774600
H	2.76701700	-2.47586800	-0.58076200
H	5.26661400	-2.33215600	-0.62384700
O	2.43428700	2.01746500	-0.09260200
O	0.60133900	3.18364400	0.06942700
O	0.46688300	-1.53638600	-0.42411400
C	-1.10692800	0.85376900	-0.08881900
C	-1.88703700	2.12061100	0.09029300
H	-1.56829800	2.62126800	1.00398900
H	-1.66746300	2.80285300	-0.73061900
H	-2.95519400	1.92634600	0.13057400
O	6.63387300	-0.07962400	-0.46819700
C	7.37725100	-0.54010400	0.57848000
C	8.83725100	-0.37914700	0.29621200
H	9.05054300	0.66436500	0.06231500
H	9.41253900	-0.69925700	1.16028300
H	9.10161100	-0.97876400	-0.57614100
O	6.88265600	-0.99892700	1.56914900
N	-1.77586000	-0.28130200	-0.19013900
H	-1.21946500	-1.13524400	-0.31488600
C	-3.20034000	-0.43018100	-0.12580900
C	-3.97522100	-0.19271900	-1.27874400
C	-3.81793800	-0.46709300	1.14473200
C	-5.35526400	-0.10471600	-1.18566300
H	-3.47728600	-0.10066100	-2.23537800
C	-5.18293000	-0.38205800	1.23588100
C	-5.96551500	-0.19994300	0.06230400
H	-5.94491500	0.04759900	-2.07890700
H	-3.21845300	-0.58539700	2.03876600
O	-5.79450300	-0.45472300	2.43720200
O	-7.28213800	-0.12639400	0.30020700
O	-2.94560400	-2.45502500	-0.59703800
H	-3.84724400	-2.77119000	-0.43909700
H	-6.74793100	-0.35459100	2.30969800
C	-8.16445500	0.07884100	-0.80380500
H	-8.08197000	-0.74751700	-1.51197800
H	-9.16440800	0.10915600	-0.38153700
H	-7.93804700	1.02568100	-1.29715900

C-2’’

G= -1429.029440 au

0 2	x	y	z
C	1.16556100	2.12353200	-0.23278400

C	3.18513400	0.81493600	-0.30206100
C	2.49848100	-0.39114000	-0.28574400
C	1.02538200	-0.38916600	-0.25628400
C	0.37624100	0.90457300	-0.28330000
H	5.10186300	1.80371200	-0.35681800
C	4.57655700	0.85776400	-0.34400200
C	3.22580900	-1.58700000	-0.31306800
C	4.60541400	-1.57362300	-0.35870200
C	5.26029300	-0.34089200	-0.37227700
H	2.67804100	-2.52092000	-0.30340800
H	5.18004200	-2.49038300	-0.38526600
O	2.54033600	2.01042600	-0.26913200
O	0.75583500	3.25711300	-0.14881900
O	0.41789600	-1.47053900	-0.22865500
C	-1.04366800	1.00563100	-0.34177800
C	-1.76327100	2.30561400	-0.53493200
H	-1.74027900	2.89491300	0.38352100
H	-1.25263100	2.89238300	-1.29498800
H	-2.79438700	2.13186000	-0.83343700
O	6.64091300	-0.28727000	-0.48339800
C	7.39280200	-0.74057200	0.56085200
C	8.84985600	-0.64294900	0.23758600
H	9.09691400	0.38862100	-0.01599400
H	9.43493100	-0.97299700	1.09125100
H	9.06801600	-1.26306600	-0.63306100
O	6.90743700	-1.14522900	1.57917500
N	-1.76351900	-0.10511500	-0.28018100
H	-1.20770000	-0.97155800	-0.23599000
C	-3.18012300	-0.21688800	-0.25561500
C	-3.81157700	-1.04654700	-1.15057200
C	-3.91281300	0.41749700	0.78077000
C	-5.20745200	-1.21546300	-1.09709800
H	-3.23631600	-1.56402300	-1.90773300
C	-5.31096100	0.31079100	0.76974500
C	-5.95434500	-0.53931900	-0.15078700
H	-5.68638700	-1.86971200	-1.81295600
O	-6.02337300	0.98939700	1.67743200
O	-7.29920700	-0.58839200	0.00154100
H	-3.45642000	1.17486300	1.40310000
O	-3.56230500	-0.89992200	2.34308100
H	-3.82852900	-1.71462200	1.88922500
H	-6.95965300	0.76654300	1.56899400
C	-8.03786300	-1.40235000	-0.90361100
H	-7.72874400	-2.44659600	-0.81870700

H	-9.08025900	-1.30178200	-0.61487800
H	-7.90086300	-1.05267600	-1.92941500

C-3’’

G= -1429.032272 au

0 2	x	y	z
C	1.26897100	2.15817200	-0.15881200
C	3.26727600	0.81861400	-0.26190100
C	2.56084300	-0.37611400	-0.26020600
C	1.08836900	-0.35013300	-0.22534800
C	0.45958200	0.95338800	-0.22689300
H	5.20016800	1.77603100	-0.30952400
C	4.65912200	0.83891600	-0.30830900
C	3.26787800	-1.58339900	-0.30638700
C	4.64737100	-1.59226100	-0.35586500
C	5.32263200	-0.37047100	-0.35481400
H	2.70477300	-2.50818600	-0.30779400
H	5.20658700	-2.51797600	-0.39646600
O	2.64177300	2.02349000	-0.20877900
O	0.87952500	3.29669700	-0.04957600
O	0.46382600	-1.42262400	-0.21338500
C	-0.96039100	1.07738900	-0.27343000
C	-1.65841100	2.39228200	-0.43818100
H	-1.62880300	2.95639300	0.49587400
H	-1.13586200	2.98911800	-1.18159000
H	-2.69199900	2.24304600	-0.73897200
O	6.70378600	-0.33878100	-0.46874400
C	7.45002100	-0.81544000	0.56924300
C	8.90773800	-0.74310500	0.24236900
H	9.48923900	-1.07752100	1.09678200
H	9.11310700	-1.37358100	-0.62406500
H	9.17158600	0.28206000	-0.01959600
O	6.95990100	-1.22052600	1.58510900
N	-1.69283300	-0.02536200	-0.22171300
H	-1.14225700	-0.89715000	-0.19680900
C	-3.11245800	-0.13494800	-0.20139900
C	-3.72598300	-0.95806800	-1.15546600
C	-3.86084300	0.50190000	0.76747200
C	-5.10371100	-1.12860600	-1.16282100
H	-3.11553700	-1.45113100	-1.90186500
C	-5.25133600	0.29931100	0.80999900
C	-5.88087400	-0.47905000	-0.20866600
H	-5.56362400	-1.74768100	-1.92044700
H	-3.40436600	1.12519100	1.52631500

O	-5.99127600	1.06545300	1.62473100
O	-7.21365200	-0.50369800	-0.12184000
O	-5.30456800	-1.44177300	2.03218300
H	-4.68385500	-2.05356400	1.60864400
H	-6.90630900	0.75219100	1.59656000
C	-7.93100900	-1.37840100	-0.99309000
H	-7.78835200	-1.07887100	-2.03286700
H	-7.60091300	-2.40859000	-0.84554700
H	-8.97698800	-1.27800000	-0.71921800

C-4’’

G= -1429.032310 au

0 2	x	y	z
C	1.30339100	2.18911700	-0.12629400
C	3.28765200	0.83011000	-0.23806100
C	2.56897200	-0.35729000	-0.25664100
C	1.09728900	-0.31714200	-0.23076000
C	0.48095800	0.99338500	-0.21730500
H	5.23022900	1.76822400	-0.26079400
C	4.67973600	0.83675000	-0.27565300
C	3.26403800	-1.57104100	-0.31515400
C	4.64346200	-1.59345400	-0.35654800
C	5.33143100	-0.37862400	-0.33440000
H	2.69181100	-2.49005400	-0.33278400
H	5.19279200	-2.52448400	-0.40679600
O	2.67420900	2.04085100	-0.17232300
O	0.92422200	3.32883800	0.00108500
O	0.46099500	-1.38244700	-0.23994600
C	-0.93514700	1.13379700	-0.27185100
C	-1.61577400	2.45769900	-0.43967000
H	-1.60465400	3.01608800	0.49819400
H	-1.06813200	3.05415800	-1.16500600
H	-2.64213900	2.32211100	-0.76999000
O	6.71291300	-0.35735800	-0.44120600
C	7.45203900	-0.88866400	0.57547800
C	8.91138200	-0.81259900	0.25686200
H	9.48185700	-1.27962700	1.05464300
H	9.10357900	-1.31215000	-0.69310800
H	9.20092000	0.23382400	0.15001500
O	6.95596100	-1.33217200	1.57207000
N	-1.67954400	0.03511700	-0.23964200
H	-1.13265800	-0.84075100	-0.23994200
C	-3.09316300	-0.07338200	-0.20716100
C	-3.69862800	-0.98901700	-1.07775700

C	-3.84549900	0.65028400	0.71297400
C	-5.06791300	-1.16320500	-1.05595700
H	-3.08789800	-1.54209400	-1.78002200
C	-5.21944300	0.48005700	0.74370700
C	-5.85022300	-0.45657500	-0.12252700
H	-5.53808400	-1.85814600	-1.73864400
H	-3.38270600	1.33223200	1.41479400
O	-5.96503000	1.18213100	1.60572800
O	-7.19216500	-0.39052400	-0.11486500
O	-5.74811700	-1.92702100	1.48268800
H	-4.84239000	-2.26210500	1.40004500
H	-6.88841000	0.90600900	1.50966900
C	-7.90465100	-1.45838400	-0.73909800
H	-7.54084300	-2.41243800	-0.35441500
H	-8.94905900	-1.31609600	-0.47557400
H	-7.79077800	-1.40983500	-1.82375700

C-5’’

G= -1429.029238 au

0 2	x	y	z
C	1.29208400	2.15646200	-0.28520600
C	3.27762200	0.79419100	-0.31164500
C	2.55954600	-0.39169800	-0.24223600
C	1.08730700	-0.34977600	-0.20335400
C	0.47126800	0.95780600	-0.27716600
H	5.21922300	1.72921000	-0.41373000
C	4.66966700	0.79851300	-0.36003200
C	3.25425600	-1.60666000	-0.22311200
C	4.63356300	-1.63135900	-0.27399900
C	5.32059400	-0.41824100	-0.34084600
H	2.68238200	-2.52473700	-0.17305100
H	5.18494400	-2.56272500	-0.26548600
O	2.66349900	2.00621500	-0.32748200
O	0.91322100	3.30325600	-0.24620000
O	0.45297900	-1.41337700	-0.12699500
C	-0.94732600	1.09344900	-0.31408700
C	-1.63731700	2.40277200	-0.54766700
H	-1.59095600	3.02252200	0.34978900
H	-1.12131700	2.94936500	-1.33340100
H	-2.67611600	2.24303300	-0.82549900
O	6.70265600	-0.41340000	-0.45462100
C	7.43698600	-0.77243900	0.63721200
C	8.89753400	-0.75831400	0.31516400
H	9.09518800	-1.47076500	-0.48691700

H	9.18221900	0.23287700	-0.03941100
H	9.46798000	-1.02135000	1.20137700
O	6.93512600	-1.04779000	1.69013800
N	-1.69262200	0.00575400	-0.18836000
H	-1.15752200	-0.87203400	-0.12176400
C	-3.11379800	-0.07059700	-0.12227900
C	-3.77889000	-0.90980900	-0.97961900
C	-3.80630800	0.65932300	0.86784500
C	-5.18073700	-1.08169600	-0.85114400
H	-3.24030800	-1.46293400	-1.73794500
C	-5.17595800	0.56750300	0.95405300
C	-5.88222200	-0.27610000	0.06974800
H	-5.71732200	-1.58185600	-1.64369500
H	-3.27038700	1.28833400	1.56836900
O	-5.84837900	1.27834600	1.88840000
O	-7.20661600	-0.28406700	0.24688500
O	-5.40061400	-2.78381700	0.25708200
H	-4.71633600	-2.58552700	0.91415700
H	-6.78988800	1.07020900	1.82003700
C	-8.00491500	-1.11693400	-0.59926000
H	-7.93943700	-0.77148500	-1.63272700
H	-7.67320400	-2.15187000	-0.51574100
H	-9.02349000	-1.01185300	-0.23771200

C-6’’

G= -1429.033061 au

0 2	x	y	z
C	1.18588900	2.19401500	-0.27304700
C	3.17104200	0.83113500	-0.30979600
C	2.45233200	-0.35544900	-0.26653300
C	0.97879900	-0.31509900	-0.23821500
C	0.36282500	0.99579500	-0.29200200
H	5.11326300	1.76787900	-0.37989100
C	4.56334300	0.83644100	-0.34665600
C	3.14708200	-1.57041100	-0.26205500
C	4.52696800	-1.59436900	-0.30160200
C	5.21423900	-0.38045800	-0.34227200
H	2.57466400	-2.48901300	-0.23200600
H	5.07853500	-2.52568200	-0.30394500
O	2.55722800	2.04344600	-0.30980600
O	0.80896600	3.34048200	-0.21387100
O	0.34464400	-1.37784700	-0.18748600
C	-1.05329900	1.13630200	-0.33362100
C	-1.73376100	2.45342300	-0.55619000

H	-1.69269500	3.06360200	0.34791300
H	-1.20690600	3.00545300	-1.33062300
H	-2.77019100	2.30394800	-0.84674300
O	6.59754200	-0.37454500	-0.44289300
C	7.32039700	-0.72823400	0.65816700
C	8.78470800	-0.70648800	0.35402200
H	9.06946600	0.28950200	0.01294500
H	9.34545500	-0.97526700	1.24467100
H	8.99531500	-1.40981000	-0.45269800
O	6.80713400	-1.00420900	1.70546900
N	-1.80766000	0.05034000	-0.23165200
H	-1.30388400	-0.84729500	-0.17571000
C	-3.21749700	-0.03590700	-0.14241200
C	-3.85706800	-1.01019500	-0.92968300
C	-3.93016600	0.70929000	0.78799300
C	-5.25359100	-1.17662900	-0.82717200
H	-3.32189700	-1.46727800	-1.74926100
C	-5.29889200	0.52265700	0.89670900
C	-5.96619400	-0.43050900	0.08397200
H	-5.74499300	-1.89862600	-1.46486000
H	-3.43876400	1.41384600	1.44705600
O	-5.99888400	1.23543500	1.79240600
O	-7.29618300	-0.49317900	0.31297600
O	-2.92795500	-2.57957600	0.23248600
H	-3.54281800	-2.51443400	0.97907200
H	-6.92816500	0.96674100	1.74697600
C	-8.05448400	-1.43564300	-0.43972900
H	-7.99815100	-1.20443800	-1.50567700
H	-7.69112300	-2.44914200	-0.25591100
H	-9.07944800	-1.34040800	-0.09325400

Cartesian coordinates (Transition states-A-4OH, RAF mechanisam)

C-3

G= -1429.029157 au

0 2	x	y	z
C	0.79366200	1.92988500	-0.33112500
C	2.84421400	0.68933900	-0.34755000
C	2.25804800	-0.46568700	0.15574800
C	0.82791000	-0.49096000	0.47690300
C	0.06266100	0.75990700	0.22822800
H	4.63960600	1.65249400	-1.04706500
C	4.19857200	0.74527500	-0.65546400

C	3.06134100	-1.59699500	0.35566400
C	4.40739700	-1.57053800	0.05526800
C	4.95755100	-0.38988900	-0.44793500
H	2.59755800	-2.49275600	0.74934400
H	5.03569800	-2.43957600	0.20110800
O	2.13181400	1.83497900	-0.55805500
O	0.28512300	3.00182600	-0.53147100
O	0.29883400	-1.49741200	0.94286600
C	-1.31349700	0.60824900	-0.22007900
C	-1.97119600	1.52407400	-1.20546200
H	-2.36157400	2.41540200	-0.70810200
H	-1.25564000	1.85853300	-1.95179500
H	-2.79449800	1.00805900	-1.69632500
O	6.29036500	-0.34530000	-0.81872000
C	7.23840400	-0.44247700	0.15945500
C	8.60963200	-0.40990300	-0.43600100
H	8.75631100	0.54476400	-0.94341800
H	9.34994100	-0.53399000	0.34919000
H	8.70449100	-1.20232000	-1.17896700
O	6.95940600	-0.52746800	1.32137700
N	-1.99794600	-0.41285300	0.26996500
H	-1.45885700	-1.06127300	0.84966700
C	-3.36379200	-0.73986200	0.02184700
C	-3.68261200	-2.01799000	-0.40927200
C	-4.36161100	0.20621300	0.27766800
C	-5.01838600	-2.36072800	-0.61155600
H	-2.89656200	-2.73970600	-0.59393200
C	-5.68580700	-0.13843300	0.06407300
C	-6.01650300	-1.43030600	-0.38523900
H	-5.29368700	-3.35108000	-0.95215700
H	-4.09157700	1.18457800	0.65213700
O	-6.75583100	0.67071600	0.26099800
O	-7.31445000	-1.76099500	-0.58918200
H	-7.86320600	-0.99180200	-0.38420000
C	-6.50949700	1.99948600	0.70625400
H	-6.01487600	1.99169700	1.68042900
H	-5.89676100	2.53914700	-0.01999800
H	-7.48345800	2.47324500	0.79231500
O	-0.05154100	1.29971300	1.94132900
H	-0.45599100	2.17413600	1.85033100

C-5

G= -1429.023237 au

0 2	x	y	z

C	0.64934000	2.09901800	-0.42098600
C	2.75185800	0.92799000	-0.38058900
C	2.14665400	-0.31122700	-0.38366400
C	0.68350800	-0.40805300	-0.32667200
C	-0.05185300	0.82724500	-0.43475000
H	4.60574500	2.03550200	-0.35465200
C	4.14323300	1.05701600	-0.36822900
C	2.95105300	-1.48062800	-0.35027900
C	4.34944800	-1.36101200	-0.41119000
C	4.91366400	-0.09677200	-0.38327900
H	4.97750200	-2.24127100	-0.45131000
O	2.03254300	2.07768300	-0.38595600
O	0.16425100	3.20468400	-0.41050200
O	0.16516500	-1.53090600	-0.18841300
C	-1.47929600	0.82189200	-0.51499700
C	-2.27859600	2.05288200	-0.81250800
H	-2.31747300	2.69779800	0.06756300
H	-1.78834000	2.62328500	-1.59793500
H	-3.28891300	1.78883900	-1.11391500
O	6.28703900	0.06010000	-0.45532000
C	7.04225000	-0.37261700	0.59856700
C	8.49597100	-0.16139700	0.32006900
H	8.67447100	0.89030100	0.09386400
H	9.07986200	-0.46800600	1.18322000
H	8.78099200	-0.74569800	-0.55612200
O	6.55809400	-0.84455200	1.58722100
N	-2.11824300	-0.32566200	-0.37232800
H	-1.50861200	-1.14440500	-0.24136100
C	-3.53138000	-0.54025400	-0.39278800
C	-4.06425300	-1.41190400	-1.32815800
C	-4.34214800	0.07875900	0.56316100
C	-5.43519100	-1.66654500	-1.32789400
H	-3.41740000	-1.88435900	-2.05673400
C	-5.70469100	-0.17177700	0.55223800
C	-6.25457300	-1.05072400	-0.39995700
H	-5.87591900	-2.34218000	-2.05048400
H	-3.89837400	0.73669400	1.29900900
O	-6.61401200	0.35587800	1.40844200
O	-7.58757400	-1.29436100	-0.39937300
H	-7.99252100	-0.78024500	0.31221500
C	-6.14614400	1.26796100	2.39522300
H	-5.43104000	0.77915400	3.06100000
H	-5.68341400	2.13820400	1.92355200
H	-7.02214300	1.57584200	2.95935100

H	2.48378400	-2.42528500	-0.59520700
O	2.70241600	-1.95652700	1.60377400
H	1.75299000	-2.12963700	1.48534200

C-6

G= -1429.024805 au

0 2	x	y	z
C	0.51576500	2.09263500	-0.34073900
C	2.64370300	0.96437200	-0.25119100
C	2.04847800	-0.29700700	-0.15446400
C	0.57800700	-0.41532300	-0.20138300
C	-0.16794900	0.81374300	-0.34732300
H	4.46723900	2.09650500	-0.44884800
C	4.02880100	1.11846600	-0.30076000
C	2.85977500	-1.41116700	-0.04922400
C	4.26245100	-1.28924400	0.04478900
C	4.81842700	-0.00407000	-0.20299800
H	2.40265400	-2.38976700	0.02266000
O	1.90574700	2.09083400	-0.32962600
O	0.02408300	3.19394900	-0.33685600
O	0.06215100	-1.54007300	-0.12894400
C	-1.59128000	0.79400400	-0.47303800
C	-2.39641200	2.02046600	-0.77654400
H	-2.45837000	2.65939400	0.10636800
H	-1.89681300	2.60064900	-1.54899400
H	-3.39781200	1.74963800	-1.10022900
O	6.15836900	0.19479600	-0.45464700
C	7.15748400	-0.47832700	0.17789700
C	8.47796400	-0.13679900	-0.43199300
H	8.62304700	0.94356800	-0.40571700
H	9.27049900	-0.64130600	0.11302500
H	8.47919700	-0.44948100	-1.47744500
O	6.96990500	-1.22635400	1.09769700
N	-2.22359200	-0.36186100	-0.36688400
H	-1.60817000	-1.17640900	-0.23652800
C	-3.63410100	-0.58655300	-0.41379400
C	-4.14576900	-1.45251500	-1.36620400
C	-4.46540600	0.01693700	0.53452800
C	-5.51470300	-1.71703400	-1.39059500
H	-3.48388900	-1.91278300	-2.08911500
C	-5.82574800	-0.24256500	0.49879800
C	-6.35368800	-1.11606600	-0.47042300
H	-5.93897400	-2.38854700	-2.12674600
H	-4.03825400	0.67083500	1.28364500

O	-6.75250700	0.27089800	1.34512600
O	-7.68492400	-1.36896600	-0.49437000
H	-8.10560600	-0.86210200	0.21331100
C	-6.30872800	1.18743900	2.33877800
H	-5.59969500	0.70582900	3.01622000
H	-5.84656500	2.06160700	1.87382700
H	-7.19622300	1.48755100	2.88895100
H	4.88332800	-2.16646500	-0.07010700
O	4.29491600	-1.39220800	2.04398600
H	5.26522300	-1.40704300	2.07027800

C-7

G= -1429.022437 au

0 2	x	y	z
C	0.64868700	1.68498100	-0.99096800
C	2.72161400	0.55530100	-0.51462200
C	2.07979600	-0.56807600	-0.00093000
C	0.60473500	-0.61958700	0.01265700
C	-0.08926900	0.51233900	-0.55338300
H	4.59812600	1.52465700	-0.93363700
C	4.10288800	0.64038900	-0.55368700
C	2.84512500	-1.63655300	0.49733000
C	4.21613100	-1.58952000	0.46470700
C	4.85336400	-0.47062200	-0.13170700
H	2.32435600	-2.48939300	0.91322500
H	4.83068700	-2.39421900	0.84735200
O	2.02842700	1.62985000	-0.97384300
O	0.19268200	2.73702400	-1.37032200
O	0.04508800	-1.62160300	0.48391600
C	-1.51455800	0.51127900	-0.66585100
C	-2.27279500	1.57635100	-1.39767000
H	-2.31877700	2.48841600	-0.79954600
H	-1.74811300	1.82975200	-2.31565800
H	-3.28132300	1.23907900	-1.62245500
O	6.22062600	-0.31319900	0.04093700
C	6.62563700	0.23056600	1.22459900
C	8.11677200	0.33625700	1.26891200
H	8.55316300	-0.65650400	1.15184100
H	8.45926800	0.95356800	0.43752400
H	8.42190600	0.77363000	2.21525000
O	5.85438800	0.56299400	2.08032600
N	-2.19114700	-0.49995800	-0.14977100
H	-1.60620200	-1.23985800	0.26259300
C	-3.60897000	-0.66569800	-0.10594100

C	-4.16688400	-1.81735800	-0.63648200
C	-4.40334500	0.29615400	0.52574100
C	-5.54474000	-2.01491600	-0.55528900
H	-3.53372300	-2.55286200	-1.11667400
C	-5.77286500	0.09955300	0.59210600
C	-6.34742100	-1.06483900	0.04910000
H	-6.00430200	-2.90503800	-0.96654500
H	-3.94172200	1.17282900	0.96066500
O	-6.66747700	0.94575200	1.15994000
O	-7.68760700	-1.25182600	0.12461600
H	-8.07882200	-0.48742100	0.56868500
C	-6.17453700	2.16125700	1.71150400
H	-5.48487500	1.95793600	2.53407300
H	-5.67448800	2.75519000	0.94253400
H	-7.04370800	2.69645500	2.08385300
O	5.15104300	-1.26173000	-1.96282800
H	4.25302500	-1.52814300	-2.21737100

C-8

G= -1429.026649 au

0 2	x	y	z
C	0.59108200	1.79002000	-0.91713400
C	2.67224900	0.63352400	-0.57126200
C	2.05247600	-0.56594700	-0.27456200
C	0.58176000	-0.65161400	-0.31369100
C	-0.13068600	0.55770000	-0.66530400
C	4.07464200	0.77439600	-0.50046300
C	2.84506900	-1.67741300	0.03669700
C	4.23157000	-1.59840300	0.05015400
C	4.82949600	-0.38414700	-0.24316300
H	2.34640300	-2.61281900	0.25847800
H	4.83545900	-2.46634800	0.27471000
O	1.98170600	1.74571700	-0.89154900
O	0.13728200	2.88368300	-1.14693600
O	0.03351700	-1.73285500	-0.05363800
C	-1.55838400	0.56957400	-0.73312700
C	-2.33978900	1.76161600	-1.19434500
H	-2.30485300	2.55148200	-0.44174500
H	-1.88210500	2.16718100	-2.09393500
H	-3.37356400	1.49105900	-1.39067700
O	6.19007000	-0.22534300	-0.31774900
C	6.99332000	-0.64379600	0.71321900
C	8.42896000	-0.40289100	0.37402400
H	8.57781700	0.65378800	0.14952800

H	9.05371700	-0.70462400	1.20981800
H	8.68790800	-0.97513200	-0.51803100
O	6.55964300	-1.11927200	1.72101500
N	-2.22086500	-0.52873300	-0.41231300
H	-1.62661800	-1.33580300	-0.17983100
C	-3.63794300	-0.68539300	-0.31668700
C	-4.26425400	-1.66523300	-1.06862200
C	-4.36004000	0.10955000	0.57886300
C	-5.64051400	-1.85289400	-0.94282000
H	-3.68625800	-2.27388800	-1.75269200
C	-5.72832900	-0.07361800	0.69023300
C	-6.37270900	-1.06297800	-0.07539400
H	-6.15466200	-2.61012200	-1.52162300
H	-3.84242700	0.84988100	1.17480500
O	-6.55756500	0.62625500	1.50357500
O	-7.71121800	-1.24036300	0.04262900
H	-8.05087900	-0.59764200	0.68007600
C	-5.99636500	1.68590500	2.26957300
H	-5.25826300	1.30024200	2.97659300
H	-5.53453900	2.42796800	1.61373500
H	-6.82353000	2.13647600	2.81113300
H	4.55297500	1.64884300	-0.92069900
O	4.20047700	1.55773500	1.39247400
H	3.72849100	2.37839600	1.18082600

C-9

G= -1429.023695 au

0 2	x	y	z
C	0.93372000	2.15404000	0.19805300
C	2.82810500	0.74858800	-0.19620400
C	2.04481500	-0.26535700	-0.80727800
C	0.57626200	-0.13655800	-0.77159500
C	0.05040100	1.05242600	-0.14561900
H	4.83554700	1.50012000	0.12983800
C	4.24186500	0.68503400	-0.26407400
C	2.66766400	-1.38884000	-1.32050100
C	4.05341300	-1.51293000	-1.27405900
C	4.81252300	-0.47422800	-0.73410800
H	2.05536700	-2.17385100	-1.74565500
H	4.55408900	-2.39847900	-1.64423400
O	2.29653900	1.96841000	0.05378800
O	0.61294300	3.26118900	0.55587400
O	-0.12042700	-1.05028300	-1.24013800
C	-1.35013000	1.19172400	0.09686600

C	-1.91981400	2.29406200	0.93690000
H	-1.25629500	2.49670300	1.77363100
H	-1.99207700	3.21610100	0.35699800
H	-2.90815400	2.02007300	1.29820100
O	6.18939000	-0.62195000	-0.67593400
C	6.71006600	-0.86066100	0.56465600
C	8.19512200	-1.00784800	0.49835300
H	8.62836000	-0.11435300	0.04776200
H	8.59101200	-1.15928700	1.49842200
H	8.44254800	-1.85856500	-0.13823700
O	6.01961900	-0.93887100	1.54348500
N	-2.17173200	0.27526700	-0.38648200
H	-1.70424700	-0.49801000	-0.87860300
C	-3.59545500	0.24628400	-0.29891700
C	-4.36253300	1.30778500	-0.75214800
C	-4.19432600	-0.91722900	0.19362500
C	-5.75244600	1.22552400	-0.69218100
H	-3.88560400	2.19013900	-1.15979300
C	-5.57684600	-0.99759500	0.23782500
C	-6.36124900	0.08431700	-0.20197100
H	-6.37328700	2.04307200	-1.03701200
H	-3.57182100	-1.73281400	0.53729500
O	-6.29494900	-2.05737500	0.68512900
O	-7.71311400	0.00171100	-0.15107700
H	-7.95419100	-0.86163100	0.21078900
C	-5.57981200	-3.19963800	1.14179100
H	-4.96352000	-3.61207000	0.33948200
H	-4.95414000	-2.94255000	1.99965500
H	-6.33164800	-3.92544200	1.43924300
O	2.74312700	0.05095600	1.65980600
H	3.59710500	-0.39970700	1.77365500

C-10

G= -1429.027002 au

0 2	x	y	z
C	0.66867500	1.91456900	-0.60795100
C	2.78210800	0.78566100	-0.40740200
C	2.17278700	-0.34371300	0.19096900
C	0.69420500	-0.49885800	0.06333000
C	-0.03153600	0.66370200	-0.38356200
H	4.60360900	1.72792300	-1.05476900
C	4.14740500	0.83993400	-0.63806200
C	2.98373100	-1.49344400	0.40888700
C	4.33492600	-1.45309900	0.19052500

C	4.90619300	-0.27414900	-0.32589000
H	2.49793500	-2.39086600	0.76962200
H	4.96104500	-2.31412100	0.38221400
O	2.05630100	1.88117100	-0.69676500
O	0.18852300	3.01084700	-0.75196900
O	0.18363600	-1.58806900	0.33934500
C	-1.44491600	0.62235900	-0.55873700
C	-2.22057300	1.76114500	-1.14857000
H	-2.37060400	2.54360700	-0.40209500
H	-1.65671700	2.20609300	-1.96470600
H	-3.18718700	1.41608400	-1.50715900
O	6.24575500	-0.21993700	-0.63473600
C	7.17269000	-0.49644300	0.33597000
C	8.55073400	-0.46954200	-0.24118800
H	8.72433000	0.49353900	-0.72234800
H	9.27833000	-0.63641600	0.54782800
H	8.63532200	-1.24529500	-1.00375400
O	6.86777100	-0.70990000	1.47331600
N	-2.09911700	-0.48314300	-0.23954000
H	-1.50804500	-1.25774200	0.08283500
C	-3.51031700	-0.69852000	-0.29469800
C	-4.00134000	-1.75950100	-1.03753400
C	-4.36765900	0.12204700	0.44475700
C	-5.37390600	-2.00423600	-1.06318100
H	-3.32129000	-2.38688400	-1.60002100
C	-5.73119600	-0.11902800	0.40543500
C	-6.23776100	-1.18969600	-0.35452400
H	-5.78250700	-2.82502900	-1.63950800
H	-3.95756700	0.92562500	1.04222500
O	-6.68055900	0.59335100	1.06144800
O	-7.57252000	-1.42280800	-0.38364200
H	-8.01157400	-0.75923300	0.16522500
C	-6.25312500	1.69486800	1.85416200
H	-5.58899800	1.35718400	2.65319100
H	-5.74557000	2.43851400	1.23494600
H	-7.15459100	2.12478300	2.28185000
O	1.96146700	0.46820900	1.97686600
H	2.82815000	0.23697200	2.34390300

C-1’

G= -1429.024534 au

0 2	x	y	z
C	0.83820200	1.80027700	-0.86228600
C	2.92139000	0.64628800	-0.52249100

C	2.30451700	-0.55966100	-0.22014900
C	0.83854800	-0.65944300	-0.23764200
C	0.10388600	0.55608600	-0.58861600
H	4.77577000	1.71945600	-0.76339200
C	4.30662000	0.77397900	-0.52541600
C	3.10060800	-1.67110100	0.08668200
C	4.47653900	-1.57368200	0.08790100
C	5.05957000	-0.34254600	-0.22016600
H	2.60896200	-2.60785500	0.31679400
H	5.10272800	-2.42552500	0.31897100
O	2.20886400	1.76456800	-0.82899500
O	0.35164000	2.87105800	-1.10924300
O	0.28687300	-1.72727300	0.03909200
C	-1.35829400	0.57010500	-0.68162900
C	-2.10068900	1.72074800	-1.30944200
H	-2.04401300	2.61135400	-0.69214000
H	-1.63236300	1.94583000	-2.26809300
H	-3.13815200	1.44749300	-1.47923800
O	6.43613500	-0.22299900	-0.29653400
C	7.16652900	-0.37060700	0.84842800
C	8.62871300	-0.26513300	0.55380300
H	8.83216100	0.68066600	0.05085700
H	9.19113100	-0.33076500	1.48077300
H	8.91737000	-1.07281800	-0.12042600
O	6.65829500	-0.55182500	1.91809100
N	-2.00323300	-0.59698300	-0.60134100
H	-1.42927300	-1.40264000	-0.35459100
C	-3.41489400	-0.74588700	-0.45042000
C	-4.07258400	-1.73438200	-1.16407500
C	-4.10461100	0.06486600	0.45728100
C	-5.44284900	-1.92111500	-0.98049100
H	-3.52208400	-2.35294800	-1.86190500
C	-5.46880800	-0.11266900	0.61947600
C	-6.14248800	-1.11525300	-0.10123900
H	-5.97832200	-2.68789600	-1.52679600
H	-3.55913900	0.81598000	1.01501000
O	-6.26685800	0.60815900	1.44774000
O	-7.47825200	-1.28738900	0.06865600
H	-7.79128100	-0.63256100	0.70710100
C	-5.67227200	1.67609900	2.17534800
H	-4.91214900	1.29747600	2.86276600
H	-5.22797700	2.40381300	1.49183400
H	-6.47696600	2.14214500	2.73751800
O	-1.02228500	1.26380000	1.11840900

H	-0.89485100	0.46107200	1.64747700

C-1”

G= -1429.030388 au

0 2	x	y	z
C	0.94169500	2.02594400	-0.30141100
C	3.02824000	0.82228600	-0.29221500
C	2.40407700	-0.41610600	-0.24505900
C	0.93101600	-0.49048400	-0.23687800
C	0.21713500	0.76741300	-0.31427000
H	4.89130300	1.90772900	-0.35794100
C	4.41618000	0.93627900	-0.31778700
C	3.19238800	-1.57273100	-0.22184700
C	4.57020800	-1.48915400	-0.24970300
C	5.16087700	-0.22551000	-0.29774900
H	2.69314000	-2.53297400	-0.18689600
H	5.19205200	-2.37505700	-0.23838800
O	2.32221000	1.98240800	-0.30970900
O	0.47717700	3.14142600	-0.27692600
O	0.38324300	-1.60048900	-0.17879400
C	-1.20704200	0.79337600	-0.39176300
C	-1.99107200	2.05808700	-0.57140200
H	-1.91792400	2.66540200	0.33224300
H	-1.55943300	2.64694500	-1.37832900
H	-3.03594400	1.84659200	-0.78319400
O	6.53931300	-0.10833800	-0.39412500
C	7.29084700	-0.43363500	0.69616000
C	8.74736300	-0.27373200	0.39563100
H	9.33191900	-0.55881500	1.26573200
H	9.01243500	-0.89378300	-0.46142700
H	8.94730500	0.76575100	0.13144700
O	6.80513300	-0.78425400	1.73430100
N	-1.87117100	-0.34738500	-0.32450900
H	-1.31061100	-1.20165300	-0.22364500
C	-3.29489500	-0.49848000	-0.38109200
C	-3.92543500	-0.65955000	-1.62993800
C	-4.05516200	-0.16506400	0.76417900
C	-5.30469700	-0.59421400	-1.72255700
H	-3.31580700	-0.85378800	-2.50302500
C	-5.42435500	-0.10142700	0.66440200
C	-6.05862300	-0.31967500	-0.58830100
H	-5.81283500	-0.74367300	-2.66640900
H	-3.54113500	0.00782600	1.70021800
O	-6.28920700	0.16760700	1.66567500

O	-7.39370300	-0.25167800	-0.67119600
H	-7.75371000	-0.03997800	0.20273100
C	-5.74758300	0.42363900	2.95874100
H	-5.19270600	-0.44644700	3.31668000
H	-5.09654100	1.30020400	2.93109500
H	-6.59778100	0.61410400	3.60726700
O	-3.00142600	-2.58744600	-0.14849500
H	-3.92443000	-2.87415700	-0.08527600

C-2”

G= -1429.029464 au

0 2	x	y	z
C	0.99147900	1.99834900	-0.53227600
C	3.06545400	0.78248000	-0.39243200
C	2.42983700	-0.44811400	-0.30268100
C	0.95865000	-0.51173400	-0.34924900
C	0.25779300	0.74425200	-0.51351400
H	4.93827100	1.85126400	-0.44616900
C	4.45409900	0.88629500	-0.37183700
C	3.20712100	-1.60664500	-0.18746100
C	4.58540200	-1.53310000	-0.16759600
C	5.18811200	-0.27723700	-0.26083600
H	2.69964400	-2.56088000	-0.12063700
H	5.19844600	-2.42137800	-0.08678600
O	2.37022300	1.94495700	-0.49791800
O	0.53217600	3.11534700	-0.56591100
O	0.39695200	-1.61455000	-0.26352500
C	-1.16130600	0.77478900	-0.63642600
C	-1.92889000	2.02374900	-0.94688600
H	-1.95330200	2.68160700	-0.07612900
H	-1.42271000	2.57275600	-1.73778900
H	-2.94384100	1.78375400	-1.25482600
O	6.56927700	-0.17241700	-0.31295600
C	7.28345200	-0.47952200	0.80799800
C	8.75004300	-0.34193800	0.54863400
H	8.97011300	0.69116200	0.27522700
H	9.30521300	-0.62010500	1.43990200
H	9.03274200	-0.97768600	-0.29100900
O	6.76195000	-0.79866400	1.83867200
N	-1.83571800	-0.35989600	-0.52189400
H	-1.24775700	-1.19580400	-0.38850200
C	-3.24828300	-0.52563600	-0.53660500
C	-3.82621800	-1.40373600	-1.42030800
C	-4.02709000	0.12204900	0.45972500

C	-5.21616700	-1.61124100	-1.39548900
H	-3.21263500	-1.92644600	-2.14307100
C	-5.42567900	-0.03541100	0.41799800
C	-6.01124700	-0.93477100	-0.49415100
H	-5.68140700	-2.30017800	-2.08947700
O	-6.28702100	0.56324400	1.24942500
O	-7.35169800	-1.12564100	-0.48523600
H	-7.73932900	-0.58498200	0.21589400
C	-5.77523800	1.47895200	2.22155700
H	-5.04200300	0.97552100	2.85226700
H	-5.32903600	2.34343800	1.72554900
H	-6.63257000	1.79241200	2.80986700
H	-3.58669900	0.92177600	1.03761600
O	-3.72392300	-1.06320800	2.10590600
H	-3.95454300	-1.91048100	1.69468100

C-3”

G= -1429.031058 au

0 2	x	y	z
C	1.09670500	2.03447900	-0.42748300
C	3.15357500	0.78640400	-0.33357900
C	2.50057800	-0.43560000	-0.25225500
C	1.02805400	-0.47579200	-0.27746400
C	0.34485500	0.79101300	-0.42740700
H	5.04179900	1.82713500	-0.40024400
C	4.54382100	0.86866300	-0.33351300
C	3.26147000	-1.60768300	-0.16742700
C	4.64093900	-1.55536400	-0.16892000
C	5.26124400	-0.30757000	-0.25243800
H	2.74024600	-2.55492900	-0.10739200
H	5.24185200	-2.45379800	-0.11183000
O	2.47480400	1.96070500	-0.40886300
O	0.65322800	3.15833500	-0.42989700
O	0.45054400	-1.57086700	-0.19012600
C	-1.07471100	0.84367500	-0.55107100
C	-1.81662700	2.10336900	-0.87794100
H	-1.86824500	2.75126500	-0.00069800
H	-1.27532000	2.65469800	-1.64283800
H	-2.82264800	1.87721500	-1.22142000
O	6.64310100	-0.22369600	-0.32375700
C	7.36683500	-0.54493200	0.78691100
C	8.83192800	-0.42861500	0.50924000
H	9.09525600	-1.07095200	-0.33172900
H	9.06336600	0.60030700	0.22972100

H	9.39400900	-0.71224600	1.39444400
O	6.85373700	-0.85996100	1.82308500
N	-1.76187100	-0.28229500	-0.42486600
H	-1.17592700	-1.12117400	-0.29061000
C	-3.17469100	-0.45514800	-0.46241800
C	-3.70142500	-1.40635900	-1.35039300
C	-3.99669400	0.25502600	0.38969200
C	-5.06459500	-1.64249500	-1.39674400
H	-3.03098400	-1.94864400	-2.00573800
C	-5.38312000	-0.00143100	0.37972800
C	-5.91542800	-0.93421000	-0.55750500
H	-5.48675700	-2.36202500	-2.08639900
H	-3.58004400	0.96720400	1.08962300
O	-6.31234000	0.75778900	0.98275600
O	-7.23651000	-1.12471000	-0.61182000
H	-7.65856000	-0.56579700	0.05762800
C	-5.89741300	1.53096000	2.10900700
H	-5.37612500	0.88767200	2.81959000
H	-5.25736400	2.35603400	1.79005600
H	-6.80730800	1.92607500	2.55206400
O	-5.44026200	-1.54167100	1.84983200
H	-4.62635900	-2.03970000	1.68201700

C-4”

G= -1429.031686 au

0 2	x	y	z
C	1.11612700	2.05196200	-0.39975900
C	3.16432400	0.78969700	-0.32230500
C	2.50400200	-0.42879000	-0.24611200
C	1.03158500	-0.45979300	-0.26437600
C	0.35527000	0.81293200	-0.40383800
H	5.05867600	1.81900100	-0.38947700
C	4.55492100	0.86325000	-0.32743900
C	3.25790400	-1.60629400	-0.17278200
C	4.63758300	-1.56249100	-0.17995000
C	5.26528100	-0.31799000	-0.25736500
H	2.73107000	-2.55067800	-0.11705200
H	5.23320700	-2.46494100	-0.13148700
O	2.49298200	1.96912600	-0.38702700
O	0.67851100	3.17778400	-0.39214500
O	0.44675700	-1.55116500	-0.18238600
C	-1.06224000	0.87651900	-0.52365900
C	-1.79493000	2.13988200	-0.85772400
H	-1.85665300	2.78964300	0.01747900

H	-1.24071000	2.68828400	-1.61536600
H	-2.79634800	1.91768400	-1.21687900
O	6.64718200	-0.24189400	-0.33314800
C	7.37293800	-0.57596800	0.77255500
C	8.83765400	-0.46579900	0.49061600
H	9.07336800	0.56275500	0.21342400
H	9.40114100	-0.75514300	1.37307400
H	9.09503700	-1.10692500	-0.35315600
O	6.86149200	-0.89636600	1.80785800
N	-1.75599800	-0.24825500	-0.39919900
H	-1.16932900	-1.09080200	-0.28509700
C	-3.16301700	-0.42539700	-0.41767200
C	-3.68095000	-1.45646900	-1.20988700
C	-3.99139300	0.36004200	0.38438200
C	-5.03973600	-1.69618000	-1.22199300
H	-3.01231300	-2.04796100	-1.82259800
C	-5.35931100	0.12944600	0.36682700
C	-5.89686400	-0.93838200	-0.40891900
H	-5.47112300	-2.47448500	-1.83853500
H	-3.56587200	1.12881500	1.01442700
O	-6.28259400	0.82513200	1.04146800
O	-7.22504800	-1.06124300	-0.55267900
H	-7.66039000	-0.52800100	0.12705700
C	-5.84005200	1.82448700	1.95889200
H	-5.19953700	1.37788000	2.72218500
H	-5.30560700	2.61649000	1.43044700
H	-6.73923700	2.22733100	2.41544900
O	-5.75588800	-2.16217700	1.39962200
H	-4.80512500	-2.33272800	1.48104800

C-5”

G= -1429.027384 au

0 2	x	y	z
C	1.10511400	1.98730200	-0.65163800
C	3.14887000	0.73128900	-0.44074200
C	2.48291600	-0.47131700	-0.24928700
C	1.00969100	-0.49994000	-0.28031700
C	0.33904800	0.75821500	-0.53071000
H	5.04719900	1.74477600	-0.58662000
C	4.53989100	0.80106600	-0.43466700
C	3.23110200	-1.63626400	-0.04360700
C	4.61110800	-1.59603900	-0.03414300
C	5.24451900	-0.36811900	-0.23217100
H	2.70060000	-2.56866200	0.10323500

H	5.20277200	-2.48927300	0.11985400
O	2.48244200	1.89921200	-0.63399400
O	0.67496300	3.11128500	-0.75897000
O	0.42158700	-1.57840000	-0.10762300
C	-1.08178300	0.82020900	-0.62461800
C	-1.82055400	2.06371100	-1.01722900
H	-1.82131600	2.78145400	-0.19466000
H	-1.30882800	2.54271500	-1.84865700
H	-2.84451200	1.82960000	-1.29711200
O	6.62872300	-0.30820200	-0.29173400
C	7.32649300	-0.46407900	0.86990500
C	8.79868100	-0.42178600	0.60950100
H	9.07229200	-1.26198300	-0.03058700
H	9.05120000	0.49807200	0.08162400
H	9.33586200	-0.47933100	1.55190400
O	6.78809500	-0.60989700	1.93086100
N	-1.78348100	-0.28167700	-0.40312000
H	-1.21500200	-1.12193500	-0.22498700
C	-3.20273600	-0.40697400	-0.35420900
C	-3.82234200	-1.34583800	-1.13529800
C	-3.93265900	0.40099000	0.54988900
C	-5.21955400	-1.54480400	-1.01027100
H	-3.24948800	-1.95542700	-1.82178900
C	-5.30239200	0.27913100	0.61356400
C	-5.95950800	-0.66994200	-0.19851300
H	-5.75206800	-2.13909100	-1.74024900
H	-3.40251600	1.10342200	1.17936800
O	-6.13731500	0.99712900	1.39856000
O	-7.29287600	-0.79033900	-0.15224600
H	-7.64477800	-0.15924000	0.49327800
C	-5.57047200	2.04014100	2.18590000
H	-4.87172300	1.63010400	2.91835900
H	-5.06133300	2.76438900	1.54540900
H	-6.40227600	2.51684900	2.69644000
O	-5.27405100	-3.08438600	0.34855000
H	-6.23684400	-3.09762400	0.46177700

C-6”

G= -1429.031699 au

0 2	x	y	z
C	0.97651500	2.06116600	-0.52488000
C	3.03616700	0.82214800	-0.38249200
C	2.38612300	-0.39497000	-0.23459100
C	0.91255800	-0.44106100	-0.24975800

C	0.22499200	0.81826500	-0.45576800
H	4.92086500	1.86327700	-0.50823400
C	4.42610900	0.90802700	-0.39067600
C	3.15048500	-1.55880400	-0.09084300
C	4.52989000	-1.50352800	-0.09882100
C	5.14710900	-0.26032000	-0.24922200
H	2.63232200	-2.50304000	0.02062900
H	5.13259300	-2.39659200	0.00411500
O	2.35443700	1.98981300	-0.51654600
O	0.53196800	3.18339500	-0.57696700
O	0.33918900	-1.52974800	-0.10823100
C	-1.19326700	0.86819700	-0.56355700
C	-1.93547800	2.11163800	-0.95229900
H	-1.98596200	2.80662400	-0.11194500
H	-1.39682500	2.62174500	-1.74705700
H	-2.94163400	1.86783800	-1.28302900
O	6.52839500	-0.17552000	-0.32818200
C	7.25809700	-0.45201700	0.79078100
C	8.72151600	-0.33822400	0.50338000
H	8.94905600	0.68755900	0.20906900
H	9.28846400	-0.60741800	1.39000900
H	8.98237900	-0.99113800	-0.32996200
O	6.75089300	-0.73001400	1.84033000
N	-1.88858600	-0.24568600	-0.37369900
H	-1.33556300	-1.09940200	-0.20464300
C	-3.29235200	-0.41284100	-0.33722200
C	-3.82858200	-1.52106100	-1.02120200
C	-4.09680200	0.39649200	0.45846200
C	-5.21749200	-1.75246300	-0.96935300
H	-3.22454600	-2.03261100	-1.75641300
C	-5.46022900	0.13895000	0.51816200
C	-6.02345100	-0.94763300	-0.20351200
H	-5.65880600	-2.57004400	-1.52495000
H	-3.65541700	1.19486300	1.03890100
O	-6.35433300	0.83652100	1.23605300
O	-7.35317700	-1.17362000	-0.13161000
H	-7.75605000	-0.50132900	0.43454600
C	-5.89420800	1.95985300	1.98696200
H	-5.17747200	1.63932300	2.74544100
H	-5.44056600	2.69795900	1.32254500
H	-6.77547700	2.37964700	2.46259700
O	-2.89304000	-2.85301400	0.35789600
H	-3.53809600	-2.73084600	1.07102000
