# Supplementary figures and images for: Mechanism of Antiradical Activity of Newly Synthesized 4,7-Dihydroxycoumarin Derivatives-Experimental and Kinetic DFT Study
Source: Int J Mol Sci. 2021 Dec 9;22(24):13273. doi: 10.3390/ijms222413273 (PMC8709309; doi:10.3390/ijms222413273)

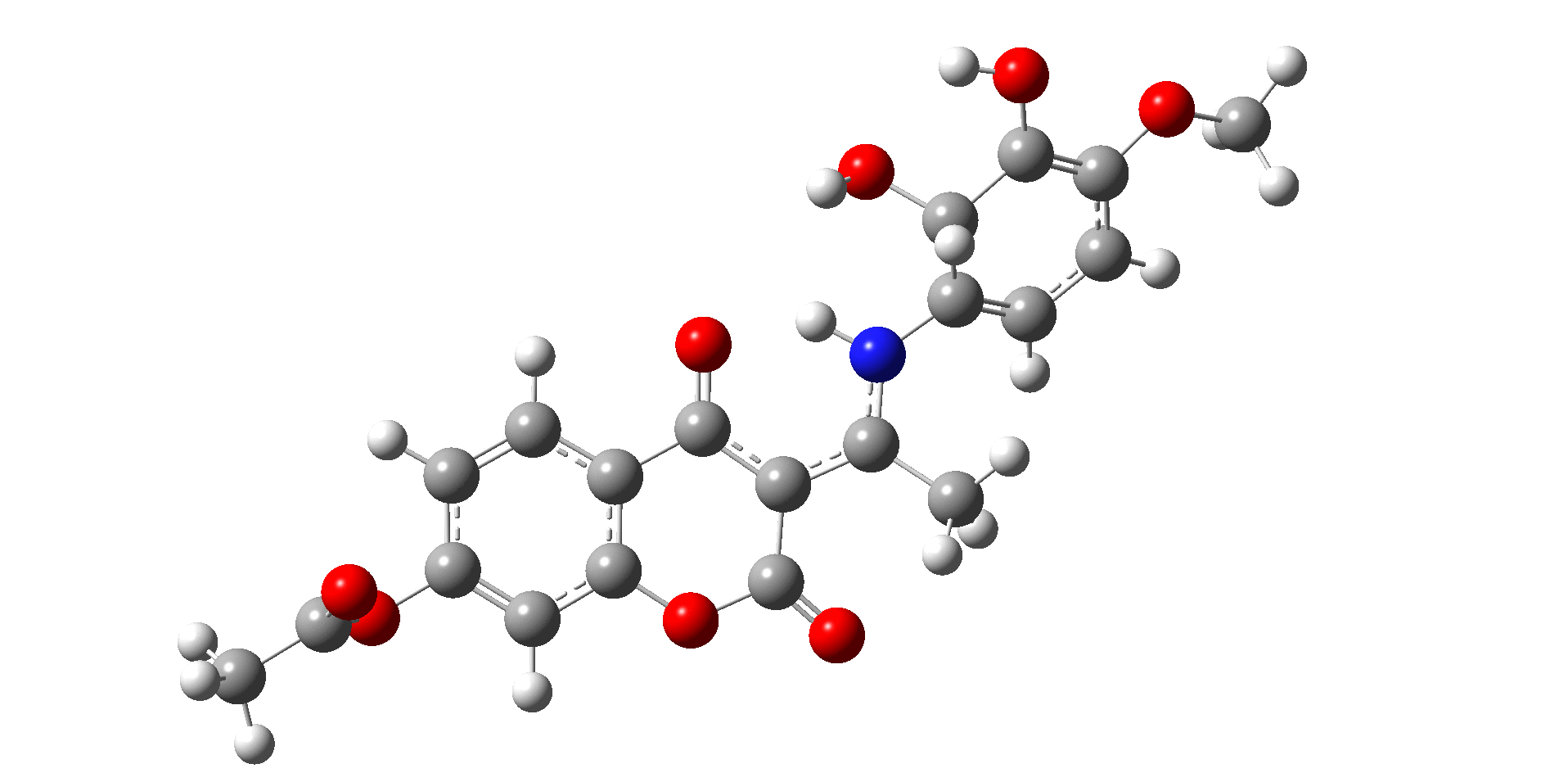

Supplement: Supplementary file 1 [file ijms-22-13273-s001.zip › Video S1.Animation of the IRC calculation for transition state at C2í»í» position (A-3OH) involved in the iHAA mechanism .gif]

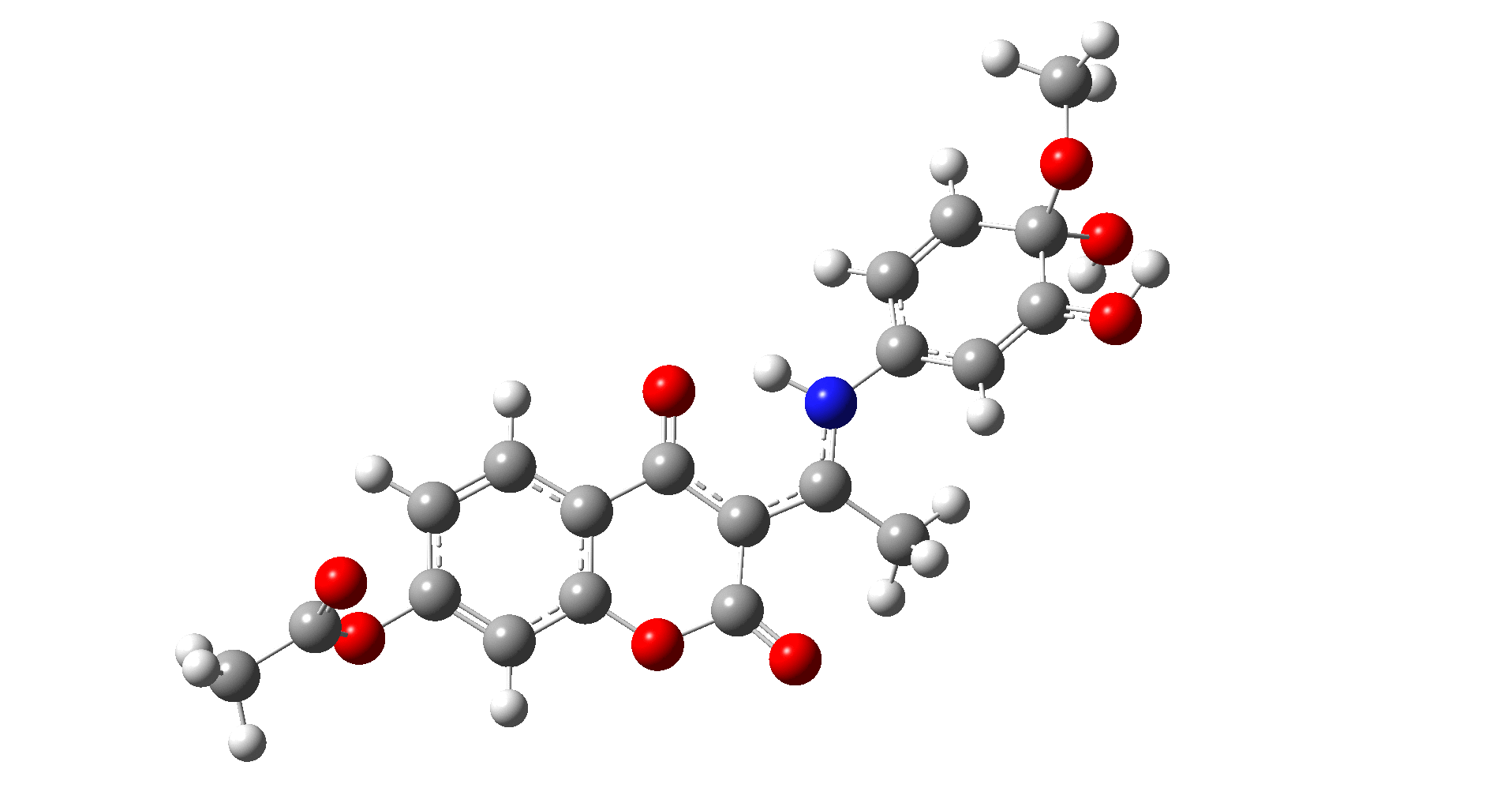

Supplement: Supplementary file 1 [file ijms-22-13273-s001.zip › Video S2.Animation of the IRC calculation for transition state at C4í»í» position (A-3OH) involved in the iHAA mechanism .gif]

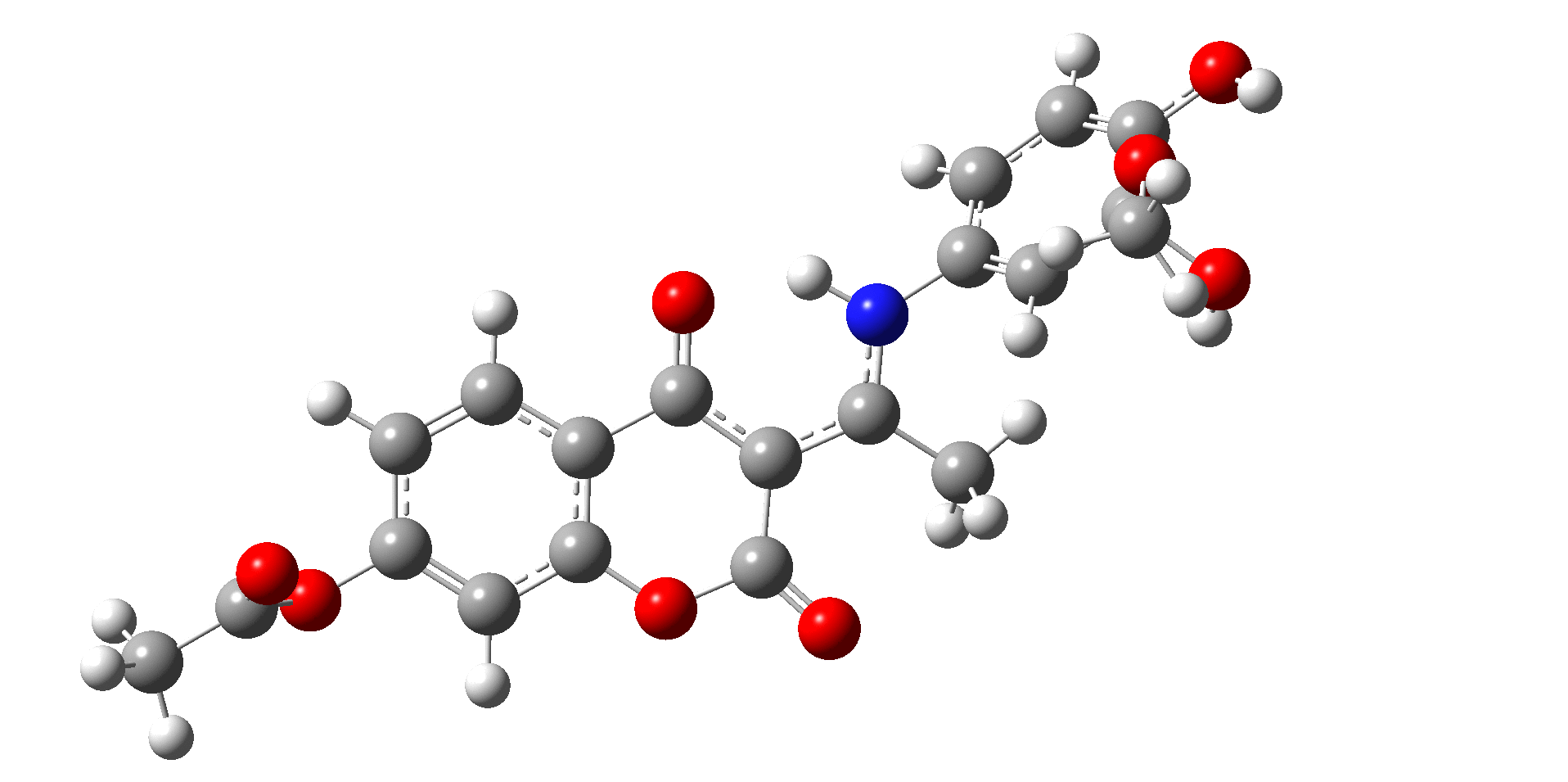

Supplement: Supplementary file 1 [file ijms-22-13273-s001.zip › Video S3.Animation of the IRC calculation for transition state at C3í»í» position (A-4OH) involved in the iHAA mechanism .gif]

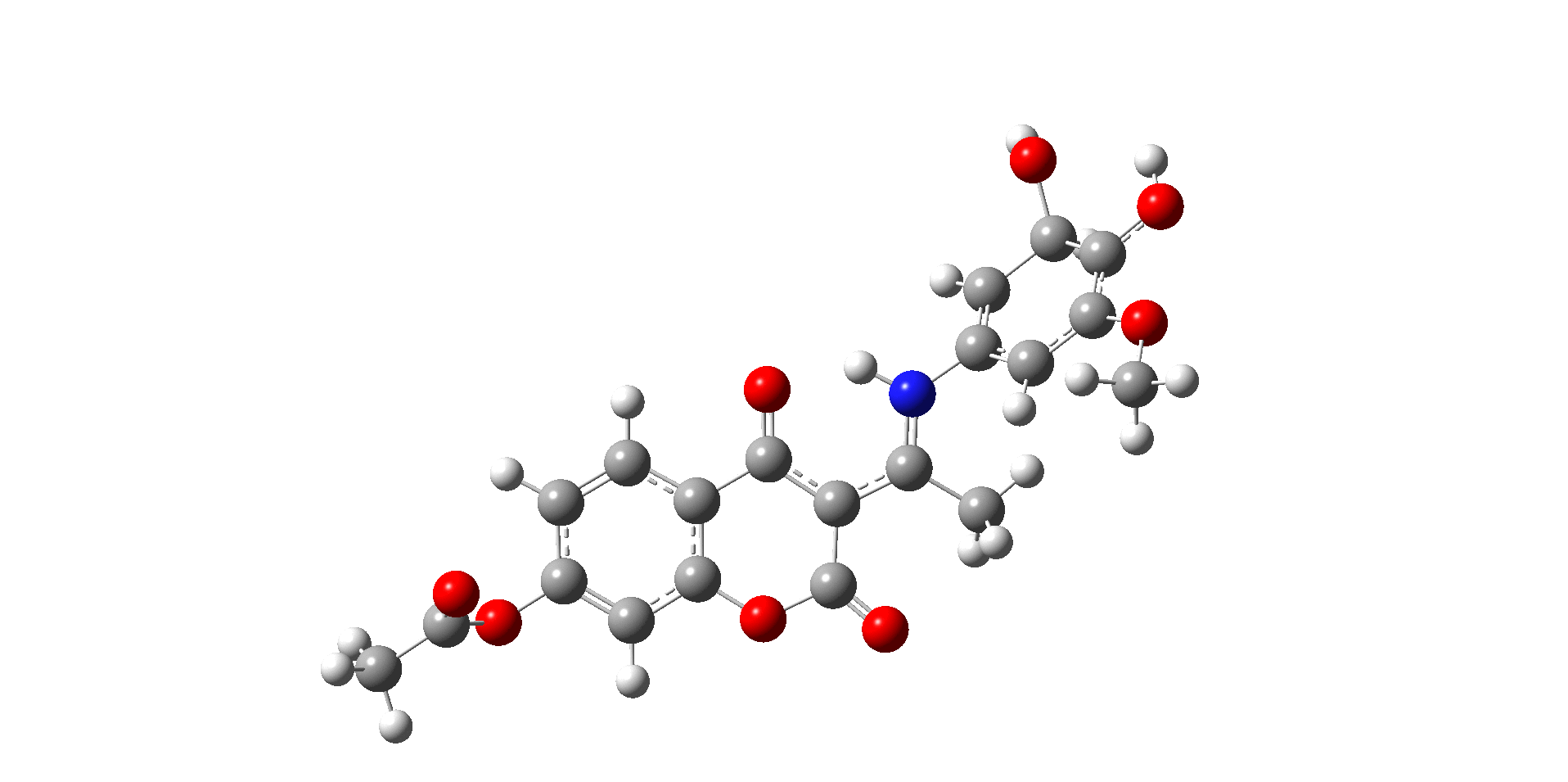

Supplement: Supplementary file 1 [file ijms-22-13273-s001.zip › Video S3.Animation of the IRC calculation for transition state at C5í»í» position (A-4OH) involved in the iHAA mechanism.gif]
